# A General Asymmetric Synthesis of (*R*)-Matsutakeol and Flavored Analogs

**DOI:** 10.3390/molecules22030364

**Published:** 2017-02-27

**Authors:** Jia Liu, Honglian Li, Chao Zheng, Shichao Lu, Xianru Guo, Xinming Yin, Risong Na, Bin Yu, Min Wang

**Affiliations:** 1 Collaborative Innovation Center of Henan Grain Crops, National Key Laboratory of Wheat and Maize Crop Science, College of Plant Protection, Henan Agricultural University, Wenhua Road No. 95, Zhengzhou 450002, China; ljia198346@163.com (J.L.); honglianli@sina.com (H.L.); 15517151076@163.com (S.L.); guoxianru@126.com (X.G.); xinmingyin@hotmail.com (X.Y.); 2 Key Laboratory of Tropical Medicinal Plant Chemistry of Hainan Province, School of Chemistry and Chemical Engineering, Hainan Normal University, Haikou 57115, China; zhengchaoustc@163.com; 3 School of Pharmaceutical Sciences, Zhengzhou University, Zhengzhou 450001, China; 4 School of Sciences, China Agricultural University, Beijing 100193, China; wangmincau@outlook.com

**Keywords:** (*R*)-matsutakeol, general method, asymmetric catalysis, mushrooms, flavored analogs

## Abstract

An efficient and practical synthetic route toward chiral matsutakeol and analogs was developed by asymmetric addition of terminal alkyne to aldehydes. (*R*)-matsutakeol and other flavored substances were feasibly synthesized from various alkylaldehydes in high yield (up to 49.5%, in three steps) and excellent enantiomeric excess (up to >99%). The protocols may serve as an alternative asymmetric synthetic method for active small-molecule library of natural fatty acid metabolites and analogs. These chiral allyl alcohols are prepared for food analysis and screening insect attractants.

## 1. Introduction

**Figure 1:**
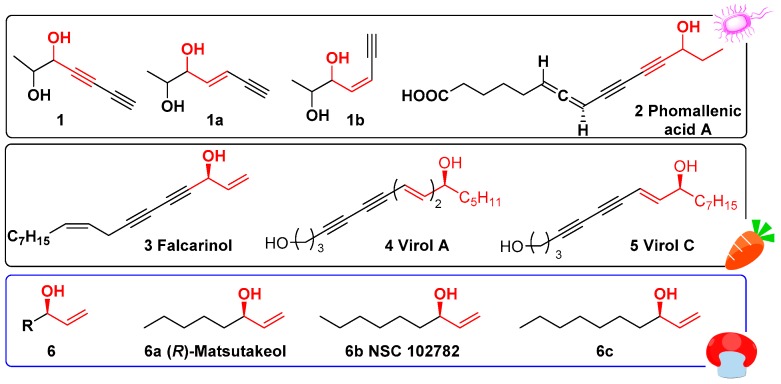
Natural unsaturated alcohols derived from fungi and plant.

Many natural unsaturated alcohols **1**–**6** (Figure 1) are very important fatty acid metabolites derived from fungi [1] and plants [2]. These substances have been found in nutrient, pharmaceutical, and agricultural use, as their structural diversity and variety of biological activities. For examples, the chiral allyl alcohols **6a**–**c** (Figure 1) are a class of important flavor substances and dietary supplements which are widely used in the food industry [3,4,5]. (*R*)-matsutakeol **6a** isolated from matsutake [6], has been found to possess antitumor properties [3]. Effects of inhibition on fungal spore germination and mushroom and plant development have also been discovered [7]. Recently, (*R*)-matsutakeol **6a** and its analogs are widely used in insecticidal compositions as effective attractants for some harmful hematophagous insects [8,9]. In particular, the enantioselectivity and chiral configuration of the compounds directly determined biological activities such as smell and taste [10]. 

Inspired by potential applications above, great attention has been paid to preparation of compounds **6** with high enantioselectivity and structural diversity [11,12,13,14,15,16,17,18,19]. To take (*R*)-matsutakeol **6a** as an example, in 1987, **6a** and its enantiomer were obtained by Helmchen through the asymmetric retro Diels-Alder reaction [11]. In the same year, Takano prepared the (*R*)-matsutakeol by using the optically active starting material of (*R*)-epichlorohydrin [12]. In 1988, Kitamura obtained (*R*)-matsutakeol (21% *ee*) via kinetic resolution of racemic allylic alcohols [13]. Oppolzer developed an enantioseletive synthesis of (*R*)-matsutakeol (96% *ee*) by catalytic asymmetric addition of divinylzinc to hexanal [14]. In 2005, a chiral resolution of racemic matsutakeol was conducted using (*S*)-(+)-2-methoxy-2-(1-naphthyl) propionic acid (MαNP acid) by Kusuda [15]. In addition, various enzymatic kinetic resolution and enzyme-catalyzed reactions have also been used in preparation of chiral matsutakeol. In 2010, Bisogno developed an efficient oxidoreductase-catalyzed system to obtain (*R*)-matsutakeol in 86% *ee* [16]. Recently, Rej prepared (*S*)-matsutakeol as an important intermediate, by ME-DKR (metal enzyme combined dynamic kinetic resolution) of (±)-oct-1-en-3-ol [17,18]. In 2016, Lee developed a synthetic strategy towards (*S*)-matsutakeol in 10 steps from inexpensive, natural (*2S*,*3S*)-d-tartaric acid with 32% yield [19].

**Scheme 1:**
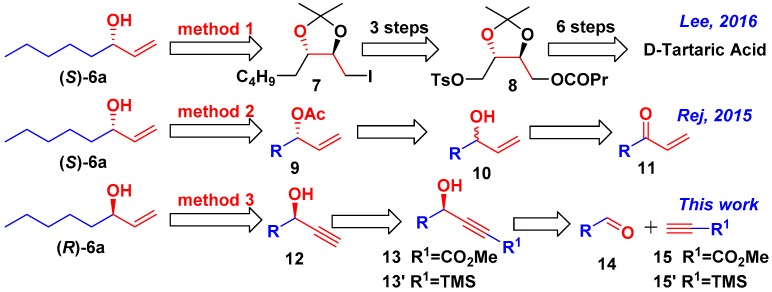
Retro-synthetic analysis of matsutakeol and its natural analogs reported in the latest literature.

**Scheme 2:**
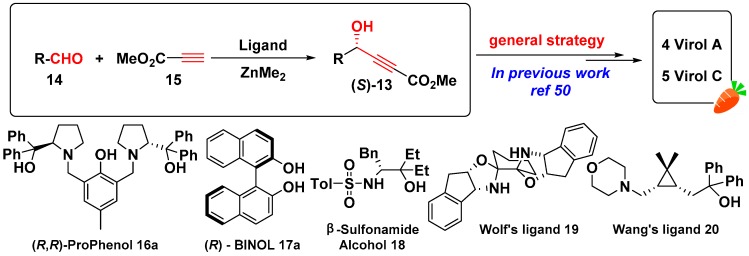
Zn-catalyzed asymmetric direct addition of terminal alkynes to aldehydes.

In summary, matsutakeol could be prepared by chiral pool synthesis [19], enzymatic kinetic resolution [17,18], and asymmetric synthesis [14] (Scheme 1). Although, several synthetic routes have been developed, there is still a lack of efficient method for obtaining a highly enantioselective allyl alcohols library in screening for flavors or attractants. Compared to asymmetric synthesis (Scheme 1, method 3), the structural diversity of the reaction products is limited by chiral sources (Scheme 1, method 1) [19], and the effect of enzymatic reaction (Scheme 1, method 2) is constrained by stability and activity of enzymes [17,18]. By retro-synthetic analysis, the chiral olefinic alcohol **6** could be formed from propargyl alcohol **12**, and chiral propargyl alcohol **12** can be synthesized from the corresponding methyl propiolate **13** (Scheme 1, method 3). Highly enantioselective methyl propiolate (*S*)-**13** is obtained by Zn-catalyzed asymmetric addition of terminal alkynes **15** to various aldehydes **14**. This reaction has been widely studied (Scheme 2) [20,21,22,23,24,25,26,27,28,29,30,31,32,33,34,35,36,37,38,39,40,41,42,43,44,45,46,47,48,49,50], in which numerous chiral ligands are applied, such as (*R*,*R*)-ProPhenol **16a** [24,39], (*R*)-BINOL **17a**, and its derivatives [28,30], β-Sulfonamide Alcohol **18** [31,32], Wolf’s ligand **19** [41], and Wang’s ligand **20** (our previous group research) [33]. Among the numerous catalysts, (*R*,*R*)-ProPhenol **16a** and (*R*)-BINOL **17a** are readily available and well-established [20,21,22,23,24,25,26,27,28,29,30,31,32,33,34,35,36,37,38,39,40,41,42,43,44,45,46,47,48,49,50].

## 2. Results and Discussion

Herein we report the asymmetric synthesis of (*R*)-matsutakeol and its natural analogs. In our previous work (Scheme 2) [50], the reaction conditions were optimized for the stereoselective addition of methyl propiolate to aliphatic aldehydes, and the highest enantiomeric excess values (97%–99% *ee*) of (*S*)-alkynol product **13** were afforded by (*R,R*)-ProPhenol/Zn complex. It is developed in a general strategy toward the total synthesis of C17 polyacetylenes such as virol A and virol C [50]. 

On the basis of previous work [24,25,39,50], we expanded substrate scope of the (*S*,*S*)-ProPhenol- catalyzed direct asymmetric addition, affording the corresponding (*R*)-configured propargyl alcohols **13** in moderated yields (63%–73%) and with excellent enantioselectivities (97%–99% *ee*) (Table 1). Thus, we developed a general method with two steps to obtain highly enantioselective allyl alcohol flavors.

**Scheme 3:**
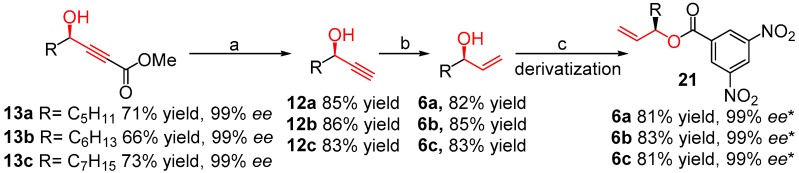
Total synthesis of (*R*)-matsutakeol and its natural analogs **6a**–**c** by using (*S*,*S*)-ProPhenol (Scheme 1, Method 3). *Reagents and conditions:* (a) (i) LiOH, THF, 0~25 °C, 1 h; (ii) CuCl, CH_3_CN,13 h; (b) Ni(OAc)_2_, NaBH_4_, EDA, EtOH, 0~25 °C; (c) 3,5-Dinitrobenzoyl chloride, Et_3_N, 0~25 °C, overnight; * The *ee* value is determined by HPLC on a chiral column after derivatization.

As shown in Scheme 3, treatment of compounds **13a**–**c** with LiOH in THF gave the corresponding carboxylic acid intermediates [38], which were then subjected to the CuCl-catalyzed decarboxylation directly, producing the chiral propargyl alcohols **12a**–**c** in good yields (83%–86%) and without loss of enantio-selectivity (99% *ee*). Reduction of compounds **12a**–**c** with NaBH_4_ in the presence of Ni(OAc)_2_ gave (*R*)-matsutakeol **6a** and its natural analogs **6b** and **6c** in good yields (82%–85%) [51]. However, the *ee* values of (*R*)-matsutakeol and analogs could not be directly resolved by HPLC on a chiral column conveniently. Therefore, derivatization of compounds **6a**–**c** through introducing 3,5-dinitrobenzoyl moiety to the natural molecules, directly afforded compounds **21a**–**c** in nearly quantitative yields (93%–95%) and 99% *ee*.

The BINOL-ZnEt_2_-Ti (IV) complex catalyzed asymmetric addition of trimethylsilylacetylene to the aliphatic aldehydes was well developed by Pu’s group [20,22,34]. It is also a practical approach for providing chiral acetylene alcohols. In the optimization procedure, to increase the catalyst loading of (*R*,*R*)-ProPhenol **16b** (entry 1 and 2) and to reduce the temperature (entry 2 and 3) could slightly improve the *ee* value, of product **13a’** (Table 2, entry 2, 72% yield, 78% *ee*). Compared to addition catalyzed by (*R*,*R*)-ProPhenol **16b**, almost no product was detected at −10 °C (Table 2, entry 4). The reaction yields and optical yields were increased on increasing the amount of BINOL (Table 2, entry 5–7). When the amount of BINOL was increased to 60%, there was no obvious improvement in yield and *ee* value (entry 7, 67% yield, 81 % *ee*). Compared to the reaction catalyzed by (*R*,*R*)-ProPhenol **16b** (entry 2, 72% yield, 78% *ee* and entry 3, 74% yield, 76% *ee*), the asymmetric addition of trimethylsilylacetylene by using BINOL as chiral ligand could be smoothly carried out at room temperature, affording higher *ee* value and almost the same yield (entry 6, 71% yield, 80% *ee*).

**Scheme 4:**
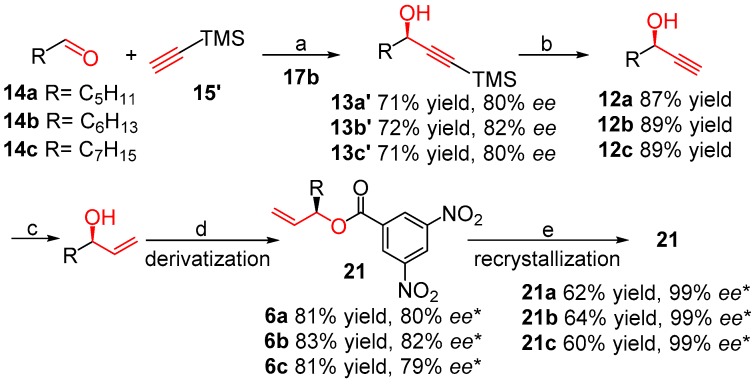
Total synthesis of (*R*)-matsutakeol and its natural analogs **6a**–**c** by using (*S*)-BINOL (Scheme 1, Method 3). *Reagents and conditions*: (a) (i) ZnEt_2_, THF, N_2_, rt; (ii) Ti(*^i^*PrO)_4_, THF, N_2_, rt; (b) K_2_CO_3_, MeOH, 0~25 °C; (c) Ni(OAc)_2_, NaBH_4_, EDA, EtOH, 0~25 °C; (d) 3,5-Dinitrobenzoyl chloride, Et_3_N, 0~25 °C, overnight; (e) Recrystallization from diethylether/*n*-hexane (1/5) at 0~25 °C, * The *ee* value is determined by HPLC on a chiral column after derivatization.

Herein, we attempted to synthesize the above natural products **6a**–**c** on gram scale, by employing the (*S*)-BINOL **17b** as the catalyst. As shown in Scheme 4, (*S*)-BINOL efficiently catalyzed the asymmetric addition of trimethylsilylacetylene with the corresponding aliphatic aldehydes **14a**–**c**, affording compounds **13a’**–**c’** in moderate yields (71%–72%) and enantioselectivities (80%–82% *ee*), slightly lower than those of compounds **13a**–**c**. K_2_CO_3_-promoted deprotection of trimethyl group of compounds **13a’**–**c’** under mild conditions gave compounds **12a**–**c** in good yields (87%–89%) [52]. Following the same procedures as shown in Scheme 3, compounds **6a**–**c** were obtained in moderate yields (81%–83%) and nearly without loss of enantioselectivities (79%–82% *ee*, the *ee* values were measured by HPLC after derived by 3,5-dinitrobenzoyl chloride). The enantiomeric purity of **21a**–**c** could be improved to over 99% *ee* by slow recrystallization from diethylether/*n*-hexane (1:5) at a low temperature. Subsequently, the esters **21a**–**c** could be quantitatively hydrolyzed to the corresponding chiral alcohols **6a**–**c** followed in our previous group research [22].

## 3. Materials and Methods

All reactions were performed under an argon atmosphere. Solvents were dried according to standard procedures and distilled before use. All reagents were purchased commercially and used without further purification, unless stated otherwise. ^1^H- and ^13^C-NMR spectra were recorded at 300 and 75 MHz, respectively. High-resolution mass spectra were recorded on an agilent instrument by the TOF MS technique. Enantiomeric excesses (*ee*) were determined by chiral HPLC analyses using a chiral column (Chiralpak OD-H, AD-H, OJ-H), and elution with isopropanol-hexane. The optical rotations were measured on PERKIN ELMER 341 Polarimeter. ^1^H-, ^13^C-NMR spectra and HPLC chromatography of the chiral products are in the Supplementary Materials.

### 3.1. General Procedure of Asymmetric Addition of Methyl Propiolate to Aliphatic Aldehydes (Table 1)

To a stirred solution of methyl propiolate (84 mg, 1 mmol), (*S*,*S*)-ProPhenol (128 mg, 0.2 mmol), triphenylphosphine oxide (111 mg, 0.4 mmol) in toluene (1 mL), dimethylzinc (2.5 mL, 1.2 M in toluene, 3 mmol) was added slowly at −10 °C. After stirring for 1.5 h at −10 °C, aldehyde (1.5 mmol) in toluene (3 mL) was added via syringe at a slow rate in 24 h at −10 °C, and quenched with water (10 mL). The mixture was filtered through a celite pad. The aqueous phase was extracted with ether. The combined organic phases were washed with saturated brine solution, dried over anhydrous Na_2_SO_4_, and concentrated under reduced pressure. The residue was purified by silica gel chromatography to get the product [25,36].

#### 3.1.1. Synthesis of (*R*)-Methyl-4-hydroxynon-2-ynoate: (*R*)**-13a**

Following the general procedure, the residue was purified by silica gel chromatography (hexanes/ethyl acetate 8:1) to offer (*R*)-**13a** (131 mg, 71% yield, 99% *ee*) as colorless oil. [α]${}_{D}^{25}$ = + 5.7 (*c* 1.05, CH_2_Cl_2_), ^1^H-NMR (300 MHz, CDCl_3_) δ (ppm): 4.57–4.44 (m, 1H), 3.76 (s, 3H), 2.66 (d, *J* = 4.7 Hz, 1H), 1.80–1.67 (m, 2H), 1.52–1.38 (m, 2H), 1.35–1.25 (m, 4H), 0.88 (t, *J* = 6.7 Hz, 3H). ^13^C-NMR (75 MHz, CDCl_3_) δ (ppm): 153.57, 88.19, 75.78, 61.71, 52.40, 36.46, 30.95, 24.23, 22.08, 13.54. HRMS ESI [M + Na]^+^ calcd for C_10_H_16_NaO_3_^+^ 207.0992, found 207.0992. Enantiomeric excess was determined by HPLC with a Chiralcel OD-H column (98:2 *n*-hexanes:isopropanol, 1.0 mL/min, 210 nm), major (*R*)-enantiomer t_r_ = 14.93 min, minor (*S*)-enantiomer t_r_ = 16.73 min.

#### 3.1.2. Synthesis of (*R*)-Methyl-4-hydroxydec-2-ynoate: (*R*)-**13b**

Following the general procedure, the residue was purified by silica gel chromatography (hexanes/ethyl acetate 8:1) to offer (*R*)-**13b** (131 mg, 66% yield, 99% *ee*) as colorless oil. [α]${}_{D}^{25}$ = + 5.8 (*c* 1.05, CH_2_Cl_2_), ^1^H-NMR (300 MHz, CDCl_3_) δ (ppm): 4.57–4.44 (m, 1H), 3.77 (s, 3H), 2.36 (d, *J* = 5.7 Hz, 1H), 1.84–1.71 (m, 2H), 1.51–1.24 (m, 8H), 0.87 (t, *J* = 6.7 Hz, 3H). ^13^C-NMR (75 MHz, CDCl_3_) δ (ppm): 153.51, 88.07, 75.85, 61.76, 52.40, 36.53, 31.25, 28.44, 24.51, 22.16, 13.62. HRMS ESI [M + Na]^+^ calcd for C_11_H_18_NaO_3_^+^ 221.1148, found 221.1150. Enantiomeric excess was determined by HPLC with a Chiralcel OD-H column (99:1 *n*-hexanes:isopropanol, 0.7 mL/min, 210 nm), major (*R*)-enantiomer t_r_ = 31.48 min, minor (*S*)-enantiomer t_r_ = 37.63 min.

#### 3.1.3. Synthesis of (*R*)-Methyl-4-hydroxyundec-2-ynoate: (*R*)-**13c**

Following the general procedure, the residue was purified by silica gel chromatography (hexanes/ethyl acetate 8:1) to offer (*R*)-**13c** (155 mg, 73% yield, 99% *ee*) as colorless oil. [α]${}_{D}^{25}$ = +6.7 (*c* 1.05, CH_2_Cl_2_), ^1^H-NMR (300 MHz, CDCl_3_) δ (ppm): 4.57–4.44 (m, 1H), 3.76 (s, 3H), 2.67 (d, *J* = 5.8 Hz, 1H), 1.76–1.70 (m, 2H), 1.46–1.42 (m, 2H), 1.35–1.23 (m, 8H), 0.92–0.82 (m, 3H). ^13^C-NMR (75 MHz, CDCl_3_) δ (ppm): 153.58, 88.22, 75.77, 61.70, 52.41, 36.50, 31.36, 28.75, 28.72, 24.56, 22.23, 13.66. HRMS ESI [M + Na]^+^ calcd for C_12_H_20_NaO_3_^+^ 235.1305, found 235.1305. Enantiomeric excess was determined by HPLC with a Chiralcel OD-H column (99:1 *n*-hexanes:isopropanol, 1.0 mL/min, 210 nm), major (*R*)-enantiomer t_r_ = 13.97 min, minor (*S*)-enantiomer t_r_ = 15.47 min.

#### 3.1.4. Synthesis of (*R*)-Methyl-4-hydroxypent-2-ynoate: (*R*)-**13d**

Following the general procedure, the residue was purified by silica gel chromatography (hexanes/ethyl acetate 8:1) to offer (*R*)-**13d** (81 mg, 63% yield, 99% *ee*) as colorless oil. [α]${}_{D}^{25}$ = +3.4 (*c* 1.05, CH_2_Cl_2_), ^1^H-NMR (300 MHz, CDCl_3_) δ (ppm): 4.57–4.44 (m, 1H), 3.80 (s, 3H), 2.97 (d, *J* = 5.6 Hz, 1H), 1.53 (d, *J* = 6.7 Hz, 3H). ^13^C-NMR (75 MHz, CDCl_3_) δ (ppm): 153.61, 88.72, 74.99, 57.53, 52.48, 22.85. HRMS ESI [M + Na]^+^ calcd for C_6_H_8_NaO_3_^+^ 151.0366, found 151.0365. Enantiomeric excess was determined by HPLC with a Chiralcel OD-H column (98:2 *n*-hexanes:isopropanol, 1.0 mL/min, 220 nm), major (*R*)-enantiomer t_r_ = 23.06 min, minor (*S*)-enantiomer t_r_ = 26.75 min.

#### 3.1.5. Synthesis of Methyl (*R*)-Methyl-4-hydroxyhex-2-ynoate: (*R*)-**13e**

Following the general procedure, the residue was purified by silica gel chromatography (hexanes/ethyl acetate 8:1) to offer (*R*)-**13e** (101 mg, 71% yield, 99% *ee*) as colorless oil. [α]${}_{D}^{25}$ = +8.2 (*c* 1.05, CH_2_Cl_2_), ^1^H-NMR (300 MHz, CDCl_3_) δ (ppm): 4.57–4.44 (m, 1H), 3.79 (s, 3H), 3.09 (d, *J* = 5.8 Hz, 1H), 1.87–1.75 (m, 2H), 1.04 (t, *J* = 7.4 Hz, 3H). ^13^C-NMR (75 MHz, CDCl_3_) δ (ppm): 153.64, 88.07, 75.76, 62.84, 52.45, 29.64, 8.88. HRMS ESI [M + Na]^+^ calcd for C_7_H_10_NaO_3_^+^ 165.0522, found 165.0522. Enantiomeric excess was determined by HPLC with a Chiralcel OD-H column (98:2 *n*-hexanes:isopropanol, 1.0 mL/min, 220 nm), major (*R*)-enantiomer t_r_ = 20.98 min, minor (*S*)-enantiomer t_r_ = 23.86 min.

#### 3.1.6. Synthesis of (*R*)-Methyl-4-hydroxyhept-2-ynoate: (*R*)-**13f**

Following the general procedure, the residue was purified by silica gel chromatography (hexanes/ethyl acetate 8:1) to offer (*R*)-**13f** (105 mg, 67% yield, 99% *ee*) as colorless oil. [α]${}_{D}^{25}$ = +5.4 (*c* 1.05, CH_2_Cl_2_), ^1^H-NMR (300 MHz, CDCl_3_) δ (ppm): 4.57–4.44 (m, 1H), 3.79 (s, 3H), 3.02 (d, *J* = 5.8 Hz, 1H), 1.83–1.66 (m, 2H), 1.58–1.41 (m, 2H), 0.96 (t, *J* = 7.3 Hz, 3H). ^13^C-NMR (75 MHz, CDCl_3_) δ (ppm): 153.64, 88.29, 75.69, 61.40, 52.45, 38.47, 17.88, 13.22. HRMS ESI [M + Na]^+^ calcd for C_8_H_12_NaO_3_^+^ 179.0679, found 179.0681. Enantiomeric excess was determined by HPLC with a Chiralcel OD-H column (98:2 *n*-hexanes:isopropanol, 1.0 mL/min, 220 nm), major (*R*)-enantiomer t_r_ = 24.14 min, minor (*S*)-enantiomer t_r_ = 25.45 min.

#### 3.1.7. Synthesis of (*R*)-Methyl-4-hydroxyoct-2-ynoate: (*R*)-**13g**

Following the general procedure, the residue was purified by silica gel chromatography (hexanes/ethyl acetate 8:1) to offer (*R*)-**13g** (116 mg, 68% yield, 99% *ee*) as colorless oil. [α]${}_{D}^{25}$ = +2.3 (*c* 1.05, CH_2_Cl_2_), ^1^H-NMR (300 MHz, CDCl_3_) δ (ppm): 4.55–4.44 (m, 1H), 3.77 (s, 3H), 2.39 (d, *J* = 5.8 Hz, 1H), 1.82–1.68 (m, 2H), 1.48–1.31 (m, 4H), 0.91 (t, *J* = 7.1 Hz, 3H). ^13^C-NMR (75 MHz, CDCl_3_) δ (ppm): 153.51, 88.06, 75.85, 61.75, 52.40, 36.23, 26.66, 21.88, 13.48. HRMS ESI [M + Na]^+^ calcd for C_9_H_14_NaO_3_^+^ 193.0835, found 193.0835. Enantiomeric excess was determined by HPLC with a Chiralcel OJ-H column (95:5 *n*-hexanes:isopropanol, 1.0 mL/min, 220 nm), major (*R*)-enantiomer t_r_ = 7.45 min, minor (*S*)-enantiomer t_r_ = 8.02 min.

#### 3.1.8. Synthesis of (*R*)-Methyl-4-hydroxy-5-methylhex-2-ynoate: (*R*)-**13h**

Following the general procedure, the residue was purified by silica gel chromatography (hexanes/ethyl acetate 8:1) to offer (*R*)-**13h** (111 mg, 71% yield, 99% *ee*) as colorless oil. [α]${}_{D}^{25}$ = +7.2 (*c* 1.05, CH_2_Cl_2_), ^1^H-NMR (300 MHz, CDCl_3_) δ (ppm): 4.27 (t, *J* = 5.9 Hz, 1H), 3.76 (s, 3H), 3.11 (d, *J* = 5.9 Hz, 1H), 1.97–1.90 (m, 1H), 1.00 (dd, *J* = 6.8, 4.4 Hz, 6H). ^13^C-NMR (75 MHz, CDCl_3_) δ (ppm): 153.64, 87.36, 67.05, 52.44, 33.79, 17.58, 17.09. HRMS ESI [M + Na]^+^ calcd for C_8_H_12_NaO_3_^+^ 179.0679, found 179.0680. Enantiomeric excess was determined by HPLC with a Chiralcel OJ-H column (95:5 *n*-hexanes:isopropanol, 1.0 mL/min, 220 nm), major (*R*)-enantiomer t_r_ = 8.66 min, minor (*S*)-enantiomer t_r_ = 10.71 min.

#### 3.1.9. Synthesis of (*R*)-Methyl-4-hydroxyoct-7-en-2-ynoate: (*R*)-**13i**

Following the general procedure, the residue was purified by silica gel chromatography (hexanes/ethyl acetate 8:1) to offer (*R*)-**13i** (114 mg, 68% yield, 97% *ee*) as colorless oil. [α]${}_{D}^{25}$ = −10.1 (*c* 1.05, CH_2_Cl_2_), ^1^H-NMR (300 MHz, CDCl_3_) δ (ppm): 5.89–5.75 (m, 1H), 5.12–4.96 (m, 2H), 4.52 (q, *J* = 6.4 Hz, 1H), 3.79 (s, 3H), 2.64 (d, *J* = 5.5 Hz, 1H), 2.26 (dd, *J* = 14.4, 6.8 Hz, 2H), 1.92–1.81 (m, 2H). ^13^C-NMR (75 MHz, CDCl_3_) δ (ppm): 153.53, 136.64, 115.46, 87.75, 76.03, 61.08, 52.48, 35.48, 28.73. HRMS ESI [M + Na]^+^ calcd for C_9_H_12_NaO_3_^+^ 191.0679, found 191.0679. Enantiomeric excess was determined by HPLC with a Chiralcel OJ-H column (95:5 *n*-hexanes:isopropanol, 1.0 mL/min, 220 nm), major (*R*)-enantiomer t_r_ = 9.01 min, minor (*S*)-enantiomer t_r_ = 9.75 min.

### 3.2. General Procedure for the Synthesis of Chiral Alkynols ***12*** from Propargyl Alcohols ***13***

A solution of the chiral alkynol (5 mmol) and THF (60 mL) were cooled to 0 °C, 1 M aq LiOH (25 mmol, 5 eq) was added at a slow rate. The solution was warmed to rt and stirred for an additional 1 h before it was quenched with 1M aq NaHSO_4_ (50 mL). The aqueous phase was extracted by ethyl acetate. The combined organic phases were dried over anhydrous Na_2_SO_4_, and concentrated under reduced pressure. The residue was dissolved in acetonitrile (12 mL), CuCl (0.5940 g, 6 mmol, 1.2 eq) was added in one portion to the mixture. The mixture was allowed to warm to r.t. and stirred for another 13 h. The aqueous phase was extracted by ether. The combined organic phases were dried over anhydrous Na_2_SO_4_, and concentrated under reduced pressure. The residue was purified by silica gel chromatography to get the product.

#### 3.2.1. Synthesis of (*R*)-Oct-1-yn-3-ol: (*R*)-**12a**

Following the general procedure, the residue was purified by silica gel chromatography (hexanes/ethyl acetate 15:1) to offer (*R*)-**12a** (536 mg, 85% yield) as colorless oil. [α]${}_{D}^{25}$ = +18.5 (*c* 1.0, ethyl ether), lit. [53] [α]${}_{D}^{25}$ = +19.3 (*c* 1.0, ethyl ether), ^1^H-NMR (300 MHz, CDCl_3_) δ (ppm): 4.33–4.27 (m, 1H), 3.10 (s, 1H), 2.40 (d, *J* = 2.1 Hz, 1H), 1.69–1.60 (m, 2H), 1.46–1.33 (m, 2H), 1.33–1.11 (m, 4H), 0.83 (t, *J* = 7.0 Hz, 3H). ^13^C-NMR (75 MHz, CDCl_3_) δ (ppm): 84.82, 72.31, 61.71, 37.15, 31.03, 24.33, 22.11, 13.53. HRMS ESI [M + Na]^+^ calcd for C_8_H_14_NaO^+^ 149.0937, found 149.0938.

#### 3.2.2. Synthesis of (*R*)-Non-1-yn-3-ol: (*R*)-**12b**

Following the general procedure, the residue was purified by silica gel chromatography (hexanes/ethyl acetate 15:1) to offer (*R*)-**12b** (603 mg, 86% yield) as colorless oil. [α]${}_{D}^{25}$ = +5.3 (*c* 2.0, CHCl_3_), lit. [54] [α]${}_{D}^{25}$ = +4.6 (*c* 1.92, CHCl_3_), ^1^H-NMR (300 MHz, CDCl_3_) δ (ppm): 4.37 (t, *J* = 5.9 Hz, 1H), 2.47 (d, *J* = 5.9 Hz, 1H), 2.38 (s, 1H), 1.75–1.68 (m, 2H), 1.52–1.41 (m, 2H), 1.38–1.27 (m, 6H), 0.89 (t, *J* = 6.7 Hz, 3H). ^13^C-NMR (75 MHz, CDCl_3_) δ (ppm): 84.78, 72.38, 61.90, 37.28, 31.35, 28.53, 24.62, 22.19, 13.66. HRMS ESI [M + Na]^+^ calcd for C_9_H_16_NaO^+^ Exact Mass: 163.1093, found 163.1093.

#### 3.2.3. Synthesis of (*R*)-Dec-1-yn-3-ol: (*R*)-**12c**

Following the general procedure, the residue was purified by silica gel chromatography (hexanes/ethyl acetate 15:1) to offer (*R*)-**12c** (640 mg, 83% yield) as colorless oil. [α]${}_{D}^{25}$ = +6.2 (*c* 1.1, CHCl_3_), lit. [55] [α]${}_{D}^{25}$ = +4.2 (*c* 1.1, CHCl_3_), ^1^H-NMR (300 MHz, CDCl_3_) δ (ppm): 4.35 (dd, *J* = 6.5, 4.9 Hz, 1H), 2.44 (d, *J* = 2.1 Hz, 1H), 2.33 (s, 1H), 1.78–1.60 (m, 2H), 1.50–1.38 (m, 2H), 1.32–1.26 (m, 8H), 0.86 (t, *J* = 6.8 Hz, 3H). ^13^C-NMR (75 MHz, CDCl_3_) δ (ppm): 84.74, 72.41, 61.97, 37.31, 31.40, 29.34, 28.83, 24.66, 22.26, 13.69. HRMS ESI [M + Na]^+^ calcd for C_10_H_18_NaO^+^ 177.1250, found 177.1250.

### 3.3. General Procedure for the Selective Reduction of the Chiral Alkynols ***12***

To a stirred solution of nickel acetate tetrahydrate (352 mg, 2 mmol) in ethanol (5 mL) under hydrogen, sodium borohydride (76 mg, 2 mmol) in ethanol (2 mL) was added at 0 °C. After stirring for 1 h at 25 °C, ethylenediamine (481 mg, 8 mmol) was added. The reaction mixture was stirred for another 10 min before chiral alkynol (2 mmol) in ethanol (2 mL) was added slowly to the reaction mixture at 0 °C. The reaction was allowed to proceed at 25 °C under hydrogen for 6 h at 25 °C. The mixture was filtered through a celite pad, diluted with ether, and concentrated under reduced pressure. The residue was purified by silica gel chromatography to get the product.

#### 3.3.1. Synthesis of (*R*)-Oct-1-en-3-ol: (*R*)-**6a**

Following the general procedure, the residue was purified by silica gel chromatography (hexanes/ethyl acetate 10:1) to offer (*R*)-**6a** (210 mg, 82% yield) as colorless oil. [α]${}_{D}^{25}$ = −10.8 (*c* 1.1, CHCl_3_), lit. [56] [α]${}_{D}^{25}$ = −10.0 (*c* 1.67, CHCl_3_), ^1^H-NMR (300 MHz, CDCl_3_) δ (ppm): 5.95–5.83 (m, 1H), 5.27–5.21 (m, 1H), 5.14–5.10 (m, 1H), 4.12 (d, *J* = 4.8 Hz, 1H), 1.61 (d, *J* = 2.8 Hz, 1H), 1.58–1.48 (m, 2H), 1.44–1.25 (m, 6H), 0.91 (t, *J* = 6.7 Hz, 3H). ^13^C-NMR (75 MHz, CDCl_3_) δ (ppm): 141.02, 114.12, 72.92, 36.68, 31.40, 24.64, 22.23, 13.64. HRMS ESI [M + Na]^+^ calcd for C_8_H_16_NaO^+^ 151.1093, found 151.1093.

#### 3.3.2. Synthesis of (*R*)-Non-1-en-3-ol: (*R*)-**6b**

Following the general procedure, the residue was purified by silica gel chromatography (hexanes/ethyl acetate 10:1) to offer (*R*)-**6b** (242 mg, 85% yield) as colorless oil. [α]${}_{D}^{25}$ = −11.5 (*c* 1.1, ethanol), lit. [57] [α]${}_{D}^{25}$ = −13.4 (*c* 1.12, ethanol), ^1^H-NMR (300 MHz, CDCl_3_) δ (ppm): 5.94–5.83 (m, 1H), 5.27–5.20 (m, 1H), 5.14–5.09 (m, 1H), 4.12 (d, *J* = 5.9 Hz, 1H), 1.67 (d, *J* = 3.5 Hz, 1H), 1.60–1.49 (m, 2H), 1.40–1.29 (m, 8H), 0.90 (t, *J* = 6.7 Hz, 3H). ^13^C-NMR (75 MHz, CDCl_3_) δ (ppm): 141.03, 114.10, 72.91, 36.72, 31.43, 28.86, 24.93, 22.22, 13.68. HRMS ESI [M + Na]^+^ calcd for C_9_H_18_NaO^+^ 165.1250, found 165.1250.

#### 3.3.3. Synthesis of (*R*)-Dec-1-en-3-ol: (*R*)-**6c**

Following the general procedure, the residue was purified by silica gel chromatography (hexanes/ethyl acetate 10:1) to offer (*R*)-**6c** (259 mg, 83% yield) as colorless oil. [α]${}_{D}^{25}$ = −16.7 (*c* 1.1, CHCl_3_), lit. [58] [α]${}_{D}^{25}$ = −18.1 (*c* 1.22, CHCl_3_), ^1^H-NMR (300 MHz, CDCl_3_) δ (ppm): 5.92–5.81 (m, 1H), 5.32–5.17 (m, 1H), 5.16–5.06 (m, 1H), 4.15–4.05 (m, 1H), 1.91 (d, *J* = 3.7 Hz, 1H), 1.58–1.46 (m, 2H), 1.40–1.22 (m, 10H), 0.89 (t, *J* = 6.7 Hz, 3H). ^13^C-NMR (75 MHz, CDCl_3_) δ (ppm): 141.04, 114.04, 72.86, 36.70, 31.45, 29.16, 28.88, 24.97, 22.27, 13.68. HRMS ESI [M + Na]^+^ calcd for C_10_H_20_NaO^+^ 179.1406, found 179.1408.

### 3.4. General Procedure for the Esterification Reaction and Determination of Enantiomeric Excess by HPLC

Triethylamine (152 mg, 1.5 mmol) and 3,5-dinitrobenzoyl chloride (277 mg, 1.2 mmol) were added to a stirred solution of the chiral alcohol (1 mmol) in CH_2_Cl_2_ (6 mL) at −5 °C. The mixture was stirred for 5 h at 25 °C before water (2 mL) was poured into the mixture at 0 °C. The aqueous phase was extracted with ether, and combined organic phases were washed with saturated brine solution, dried over anhydrous Na_2_SO_4_, and concentrated under reduced pressure. The residue was purified by silica gel chromatography to get the product.

#### 3.4.1. Synthesis of (*R*)-Oct-1-en-3-yl-3,5-dinitrobenzoate: (*R*)-**21a**

Following the general procedure, the residue was purified by silica gel chromatography (hexanes/ethyl acetate 8:1) to offer (*R*)-**21a** (306 mg, 95% yield, 99% *ee*) as white solid. [α]${}_{D}^{25}$ = −16.2 (*c* 1.1, CH_2_Cl_2_), ^1^H-NMR (300 MHz, CDCl_3_) δ (ppm): 9.22 (t, *J* = 2.1 Hz, 1H), 9.16 (d, *J* = 2.1 Hz, 2H), 5.92 (m 1H), 5.56 (q, *J* = 6.9 Hz, 1H), 5.36 (m, 2H), 1.96–1.72 (m, 2H), 1.48–1.26 (m, 6H), 0.89 (t, *J* = 6.9 Hz, 3H). ^13^C-NMR (75 MHz, CDCl_3_) δ (ppm): 161.45, 148.34, 135.09, 134.00, 129.02, 121.92, 118.05, 77.82, 33.69, 31.09, 24.40, 22.10, 13.57. HRMS ESI [M + Na]^+^ calcd for C_15_H_18_N_2_NaO_6_^+^ 345.1057, found 345.1057. Enantiomeric excess was determined by HPLC with a Chiralcel OD-H column (99:1 *n*-hexanes: isopropanol, 1.0 mL/min, 210 nm), major (*R*)-enantiomer t_r_ = 17.74 min, minor (*S*)-enantiomer t_r_ = 14.46 min.

#### 3.4.2. Synthesis of (*R*)-Non-1-en-3-yl-3,5-dinitrobenzoate: (*R*)-**21b**

Following the general procedure, the residue was purified by silica gel chromatography (hexanes/ethyl acetate 8:1) to offer (*R*)-**21b** (312 mg, 93% yield, 99% *ee*) as colorless oil. [α]${}_{D}^{25}$ = −18.3 (*c* 1.1, CH_2_Cl_2_), ^1^H-NMR (300 MHz, CDCl_3_) δ (ppm): 9.21 (t, *J* = 2.1 Hz, 1H), 9.15 (d, *J* = 2.1 Hz, 2H), 6.01–5.84 (m, 1H), 5.56 (d, *J* = 6.6 Hz, 1H), 5.34 (dd, *J* = 26.1, 13.8 Hz, 2H), 1.95–1.70 (m, 2H), 1.45–1.26 (m, 8H), 0.87 (t, *J* = 6.7 Hz, 3H). ^13^C-NMR (75 MHz, CDCl_3_) δ (ppm): 161.45, 148.33, 135.11, 133.99, 129.02, 121.92, 118.01, 77.81, 33.73, 31.27, 28.59, 24.70, 22.17, 13.63. HRMS ESI [M + Na]^+^ calcd for C_16_H_20_N_2_NaO_6_^+^ 359.1214, found 359.1214. Enantiomeric excess was determined by HPLC with a Chiralcel OD-H column (98:2 *n*-hexanes:isopropanol, 1.0 mL/min, 210 nm), major (*R*)-enantiomer t_r_ = 15.27 min, minor (*S*)-enantiomer t_r_ = 12.67 min. 

#### 3.4.3. Synthesis of (*R*)-Dec-1-en-3-yl-3,5-dinitrobenzoate: (*R*)-**21c**

Following the general procedure, the residue was purified by silica gel chromatography (hexanes/ethyl acetate 8:1) to offer (*R*)-**6c** (332 mg, 95% yield, 99% *ee*) as white solid. [α]${}_{D}^{25}$ = −21.2 (*c* 1.05, CH_2_Cl_2_), ^1^H-NMR (300 MHz, CDCl_3_) δ (ppm): 9.23 (t, *J* = 2.1 Hz, 1H), 9.16 (d, *J* = 2.1 Hz, 2H), 5.98–5.87 (m, 1H), 5.57 (q, *J* = 6.8 Hz, 1H), 5.35 (dd, *J* = 25.1, 13.8 Hz, 2H), 1.99–1.71 (m, 2H), 1.40–1.28 (m, 10H), 0.88 (t, *J* = 6.6 Hz, 3H). ^13^C-NMR (75 MHz, CDCl_3_) δ (ppm): 161.45, 148.35, 135.09, 134.02, 129.03, 121.92, 118.08, 77.85, 33.74, 31.37, 28.90, 28.75, 24.76, 22.24, 13.68. HRMS ESI [M + Na]^+^ calcd for C_17_H_22_N_2_NaO_6_^+^ 373.1370, found 373.1370. Enantiomeric excess was determined by HPLC with a Chiralcel OD-H column (98:2 *n*-hexanes:isopropanol, 1.0 mL/min, 210 nm), major (*R*)-enantiomer t_r_ = 16.89 min, minor (*S*)-enantiomer t_r_ = 13.50 min.

### 3.5. General Procedure of Asymmetric Addition of Ethynyltrimethylsilane to Aliphatic Aldehydes

To a stirred solution of ethynyltrimethylsilane (3922 mg, 40 mmol), (*S*)-BINOL (1144 mg, 4 mmol), HMPA (3584 mg, 20 mmol) in methylene chloride (120 mL), diethylzinc (40 mL, 40 mmol) was added slowly at 0 °C. After stirring for 16 h at 25 °C, Titanium(IV) isopropoxide (2842 mg, 10 mmol) was added and the stirring was continued for another 1 h at 25 °C. Then an aldehyde (10 mmol) was added and the reaction was allowed to proceed at 25 °C for another 6 h before being quenched with water (20 mL). The mixture was filtered through a celite pad. The aqueous phase was extracted with ether. The combined organic phases were washed with saturated brine, dried over anhydrous Na_2_SO_4_, and concentrated under reduced pressure. The residue was purified by silica gel chromatography to get the product [34,40].

#### 3.5.1. Synthesis of (*R*)-1-(Trimethylsilyl)oct-1-yn-3-ol: (*R*)-**13a’**

Following the general procedure, the residue was purified by silica gel chromatography (hexanes/ethyl acetate 20:1) to offer (*R*)-**13a’** (1408 mg, 71% yield, 80% *ee*) as colorless oil. [α]${}_{D}^{25}$ = +8.2 (*c* 1.1, CHCl_3_), lit. [56] [α]${}_{D}^{25}$ = +13.6 (*c* 1.1, CHCl_3_), ^1^H-NMR (300 MHz, CDCl_3_) δ (ppm): 4.38 (dd, *J* = 12.2, 6.4 Hz, 1H), 1.80 (d, *J* = 5.5 Hz, 1H), 1.76–1.68 (m, 2H), 1.47 (d, *J* = 7.3 Hz, 2H), 1.35 (dd, *J* = 7.1, 3.5 Hz, 4H), 0.92 (t, *J* = 6.7 Hz, 3H), 0.20 (s, 9H). ^13^C-NMR (75 MHz, CDCl_3_) δ (ppm): 106.61, 88.96, 62.61, 37.35, 31.04, 24.41, 22.16, 13.59, −0.47. HRMS ESI [M + Na]^+^ calcd for C_11_H_22_NaOSi^+^ 221.1332, found 221.1332. Enantiomeric excess was determined by HPLC with a Chiralcel AD-H column (99:1 *n*-hexanes:isopropanol, 1.0 mL/min, 210 nm), major (*R*)-enantiomer t_r_ = 14.72 min, minor (*S*)-enantiomer t_r_ = 13.52 min.

#### 3.5.2. Synthesis of (*R*)-1-(Trimethylsilyl)non-1-yn-3-ol: (*R*)-**13b’**

Following the general procedure, the residue was purified by silica gel chromatography (hexanes/ethyl acetate 20:1) to offer (*R*)-**13b’** (1529 mg, 72% yield, 82% *ee*) as colorless oil. [α]${}_{D}^{25}$ = +2.3 (*c* 1.1, CHCl_3_), ^1^H-NMR (300 MHz, CDCl_3_) δ (ppm): 4.38 (dd, *J* = 12.3, 6.5 Hz, 1H), 1.78–1.68 (m, 3H), 1.46 (d, *J* = 6.5 Hz, 2H), 1.38–1.27 (m, 6H), 0.92 (t, *J* = 6.7 Hz, 3H), 0.20 (s, 9H). ^13^C-NMR (75 MHz, CDCl_3_) δ (ppm): 106.68, 88.89, 62.54, 37.36, 31.34, 28.52, 24.70, 22.17, 13.67, −0.48. HRMS ESI [M + Na]^+^ calcd for C_12_H_24_NaOSi^+^ 235.1489, 235.1490. Enantiomeric excess was determined by HPLC with a Chiralcel AD-H column (98:2 *n*-hexanes:isopropanol, 1.0 mL/min, 210 nm), major (*R*)-enantiomer t_r_ = 13.68 min, minor (*S*)-enantiomer t_r_ = 12.42 min.

#### 3.5.3. Synthesis of (*R*)-1-(Trimethylsilyl)dec-1-yn-3-ol: (*R*)-**13c’**

Following the general procedure, the residue was purified by silica gel chromatography (hexanes/ethyl acetate 20:1) to offer (*R*)-**13c’** (1608 mg, 71% yield, 80% *ee*) as colorless oil. [α]${}_{D}^{25}$ = +3.2 (*c* 1.1, CHCl_3_). ^1^H-NMR (300 MHz, CDCl_3_) δ (ppm): 4.36 (dd, *J* = 12.3, 6.5 Hz, 1H), 2.00 (m, 1H), 1.83–1.61 (m, 2H), 1.53–1.40 (m, 2H), 1.40–1.31 (m, 8H), 0.90 (t, *J* = 6.7 Hz, 3H), 0.18 (m, 9H). ^13^C-NMR (75 MHz, CDCl_3_) δ (ppm): 106.70, 88.87, 62.54, 37.36, 31.39, 28.81, 28.79, 24.74, 22.26, 13.70, −0.48. HRMS ESI [M + Na]^+^ calcd for C_13_H_26_NaOSi^+^ 249.1645, found 249.1645. Enantiomeric excess was determined by HPLC with a Chiralcel AD-H column (99:1 *n*-hexanes:isopropanol, 0.9 mL/min, 210 nm), major (*R*)-enantiomer t_r_ = 18.90 min, minor (*S*)-enantiomer t_r_ = 16.94 min.

### 3.6. General Procedure for the Synthesis of chIral Alkynols ***12*** from Propargyl Alcohols ***13*****’**

To a stirred solution of chiral alkynol (10 mmol) in methanol (20 mL), potassium carbonate (2764 mg, 20 mmol) was added slowly at 0 °C. After stirring for 20 h at 25 °C, water (20 mL) was added slowly at 0 °C. The reaction mixture was concentrated under reduced pressure. The aqueous phase was extracted with ether. The combined organic phases were washed with saturated brine solution, dried over anhydrous Na_2_SO_4_, concentrated under reduced pressure. The residue was purified by silica gel chromatography to get the product.

#### 3.6.1. Synthesis of (*R*)-Oct-1-yn-3-ol: (*R*)-**12a**

Following the general procedure, the residue was purified by silica gel chromatography (hexanes/ethyl acetate 15:1) to offer (*R*)-**12a** (1098 mg, 87% yield) as colorless oil. [α]${}_{D}^{25}$ = +16.2 (*c* 1.0, ethyl ether), lit. [53] [α]${}_{D}^{25}$ = +19.3 (*c* 1.0, ethyl ether). HRMS ESI [M + Na]^+^ calcd for C_8_H_14_NaO^+^ 149.0937, found 149.0937.

#### 3.6.2. Synthesis of (*R*)-Non-1-yn-3-ol: (*R*)-**12b**

Following the general procedure, the residue was purified by silica gel chromatography (hexanes/ethyl acetate 15:1) to offer (*R*)-**12b** (1248 mg, 89% yield) as colorless oil. [α]${}_{D}^{25}$ = +4.1 (*c* 2.0, CHCl_3_), lit. [54] [α]${}_{D}^{25}$ = +4.6 (*c* 1.92, CHCl_3_). HRMS ESI [M + Na]^+^ calcd for C_9_H_16_NaO^+^ Exact mass: 163.1093, found 163.1093.

#### 3.6.3. Synthesis of (*R*)-Dec-1-yn-3-ol: (*R*)-**12c**

Following the general procedure, the residue was purified by silica gel chromatography (hexanes/ethyl acetate 15:1) to offer (*R*)-**12c** (1373 mg, 89% yield) as colorless oil. [α]${}_{D}^{25}$ = +5.1 (*c* 1.1, CHCl_3_), lit. [55] [α]${}_{D}^{25}$ = +4.2 (*c* 1.1, CHCl_3_). HRMS ESI [M + Na]^+^ calcd. for C_10_H_18_NaO^+^ 177.1250, found 177.1250.

### 3.7. General Procedure for the Selective Reduction of the Chiral Alkynols ***12***

To a stirred solution of nickel acetate tetrahydrate (1408 mg, 8 mmol) in ethanol (20 mL) under hydrogen, sodium borohydride (303 mg, 8 mmol) in ethanol (8 mL) was added at 0 °C. After stirring for 1 h at 25 °C, ethylenediamine (481 mg, 8 mmol) was added. The reaction mixture was stirred for another 10 min before chiral alkynol (8 mmol) in ethanol (8 mL) was added slowly to the reaction mixture at 0 °C. The reaction was allowed to proceed at 25 °C under hydrogen for 6 h at 25 °C. The mixture was filtered through a celite pad, diluted with ether, and concentrated under reduced pressure. The residue was purified by silica gel chromatography to get the product.

#### 3.7.1. Synthesis of (*R*)-Oct-1-en-3-ol: (*R*)-**6a**

Following the general procedure, the residue was purified by silica gel chromatography (hexanes/ethyl acetate 10:1) to offer (*R*)-**6a** (831 mg, 81% yield) as colorless oil. [α]${}_{D}^{25}$ = −7.3 (*c* 1.1, CHCl_3_), lit. [56] [α]${}_{D}^{25}$ = −10.0 (*c* 1.67, CHCl_3_). HRMS ESI [M + Na]^+^ calcd. for C_8_H_16_NaO^+^ 151.1093, found 151.1093.

#### 3.7.2. Synthesis of (*R*)-Non-1-en-3-ol: (*R*)-**6b**

Following the general procedure, the residue was purified by silica gel chromatography (hexanes/ethyl acetate 10:1) to offer (*R*)-**6b** (944 mg, 83% yield) as colorless oil. [α]${}_{D}^{25}$ = −10.5 (*c* 1.1, ethanol), lit. [57] [α]${}_{D}^{25}$ = −13.4 (*c* 1.12, ethanol). HRMS ESI [M + Na]^+^ calcd. for C_9_H_18_NaO^+^ 165.1250, found 165.1250.

#### 3.7.3. Synthesis of (*R*)-Dec-1-en-3-ol: (*R*)-**6c**

Following the general procedure, the residue was purified by silica gel chromatography (hexanes/ethyl acetate 10:1) to offer (*R*)-**6c** (1013 mg, 81% yield) as colorless oil. [α]${}_{D}^{25}$ = −14.2 (*c* 1.1, CHCl_3_), lit. [58] [α]${}_{D}^{25}$ = −18.1 (*c* 1.22, CHCl_3_). HRMS ESI [M + Na]^+^ calcd. for C_10_H_20_NaO^+^ 179.1406, found 179.1406.

### 3.8. General Procedure for the Esterification Reaction and Determination of Enantiomeric Excess by HPLC

Triethylamine (909 mg, 9 mmol) and 3,5-dinitrobenzoyl chloride (1660 mg, 7.2 mmol) were added to a stirred solution of the chiral alcohol (6 mmol) in CH_2_Cl_2_ (40 mL) at −5 °C. The mixture was stirred for 5 h at 25 °C before water (10 mL) was poured into the mixture at 0 °C. The aqueous phase was extracted with ether and combined organic phases were washed with saturated brine solution, dried over anhydrous Na_2_SO_4_, and concentrated under reduced pressure. The residue was purified by silica gel chromatography to get the product.

#### 3.8.1. Synthesis of (*R*)-Oct-1-en-3-yl-3,5-dinitrobenzoate: (*R*)-**21a**

Following the general procedure, the residue was purified by silica gel chromatography (hexanes/ethyl acetate 8:1) to offer (*R*)-**21a** (1837 mg, 95% yield, 80% *ee*) as white solid. [α]${}_{D}^{25}$ = −14.2 (*c* 1.1, CH_2_Cl_2_). Enantiomeric excess was determined by HPLC with a Chiralcel OD-H column (99:1 *n*-hexanes:isopropanol, 1.0 mL/min, 210 nm), major (*R*)-enantiomer t_r_ = 17.80 min, minor (*S*)-enantiomer t_r_ = 14.65 min.

#### 3.8.2. Synthesis of (*R*)-Non-1-en-3-yl-3,5-dinitrobenzoate: (*R*)-**21b**

Following the general procedure, the residue was purified by silica gel chromatography (hexanes/ethyl acetate 8:1) to offer (*R*)-**21b** (1987 mg, 98% yield, 82% *ee*) as colorless oil. [α]${}_{D}^{25}$ = −15.2 (*c* 1.1, CH_2_Cl_2_). Enantiomeric excess was determined by HPLC with a Chiralcel OD-H column (98:2 *n*-hexanes:isopropanol, 1.0 mL/min, 210 nm), major (*R*)-enantiomer t_r_ = 15.76 min, minor (*S*)-enantiomer t_r_ = 12.93 min.

#### 3.8.3. Synthesis of (*R*)-Dec-1-en-3-yl-3,5-dinitrobenzoate: (*R*)-**21c**

Following the general procedure, the residue was purified by silica gel chromatography (hexanes/ethyl acetate 8:1) to offer (*R*)-**21c** (2018 mg, 96% yield, 79% *ee*) as white solid. [α]${}_{D}^{25}$ = −18.2 (*c* 1.05, CH_2_Cl_2_). Enantiomeric excess was determined by HPLC with a Chiralcel OD-H column (98:2 *n*-hexanes:isopropanol, 1.0 mL/min, 210 nm), major (*R*)-enantiomer t_r_ = 16.58 min, minor (*S*)-enantiomer t_r_ = 13.66 min.

### 3.9. General Procedure for the Recrystallization to Improve Optical Purity

Dinitrobenzoates (5 mmol) were dissolved in diethyl ether at room temperature, then n-hexane was added slowly to the mixture until a white precipitate occurred. A small portion of diethyl ether was added and the white precipitate was dissolved. The mixture was cooled to −30 °C and stayed for 48 h to get a white crystal of the dinitrobenzoate.

#### 3.9.1. Recrystallization of (*R*)-Oct-1-en-3-yl-3,5-dinitrobenzoate: (*R*)-**21a**

Following the general procedure, the residue was purified by silica gel chromatography (hexanes/ethyl acetate 8:1) to offer (*R*)-**21a** (999 mg, 62% yield, 99% *ee*) as white solid. [α]${}_{D}^{25}$ = −16.1 (*c* 1.1, CH_2_Cl_2_). HRMS ESI [M + Na]^+^ calcd for C_15_H_18_N_2_NaO_6_^+^ 345.1057, found 345.1057. Enantiomeric excess was determined by HPLC with a Chiralcel OD-H column (99:1 *n*-hexanes:isopropanol, 1.0 mL/min, 210 nm), major (*R*)-enantiomer t_r_ = 18.20 min, minor (*S*)-enantiomer t_r_ = 14.46 min.

#### 3.9.2. Recrystallization of (*R*)-Non-1-en-3-yl-3,5-dinitrobenzoate: (*R*)-**21b**

Following the general procedure, the residue was purified by silica gel chromatography (hexanes/ethyl acetate 8:1) to offer (*R*)-**21b** (1076 mg, 64% yield, 99% *ee*) as colorless oil. [α]${}_{D}^{25}$ = −18.7 (*c* 1.1, CH_2_Cl_2_). HRMS ESI [M + Na]^+^ calcd for C_16_H_20_N_2_NaO_6_^+^ 359.1214, found 359.1214. Enantiomeric excess was determined by HPLC with a Chiralcel OD-H column (98:2 *n*-hexanes:isopropanol, 1.0 mL/min, 210 nm), major (*R*)-enantiomer t_r_ = 15.60 min, minor (*S*)-enantiomer t_r_ = 12.67 min.

#### 3.9.3. Recrystallization of (*R*)-Dec-1-en-3-yl-3,5-dinitrobenzoate: (*R*)-**21c**

Following the general procedure, the residue was purified by silica gel chromatography (hexanes/ethyl acetate 8:1) to offer (R)-**6c** (1051 mg, 60% yield, 99% ee) as white solid. [α]${]}_{D}^{25}$ = −21.5 (c 1.05, CH_2_Cl_2_). HRMS ESI [M + Na]^+^ calcd for C_17_H_22_N_2_NaO_6_^+^ 373.1370, found 373.1370. Enantiomeric excess was determined by HPLC with a Chiralcel OD-H column (98:2 n-hexanes:isopropanol, 1.0 mL/min, 210 nm), major (R)-enantiomer t_r_ = 16.46 min, minor (S)-enantiomer t_r_ = 13.50 min.

## 4. Conclusions

In summary, we developed a general synthetic route toward chiral matsutakeol and its analogs via the asymmetric catalytic alkynylation. A practical and efficient access was provided to prepare the (*R*)-matsutakeol (99% ee, total yield up to 50.2%, in three steps) and its highly enantioselective analogs, by utilizing the (*S*,*S*)-ProPhenol as chiral catalyst. In addition, the method may allow for gram-scale synthesis of (*R*)-matsutakeol and its analogs by using (*S*)-BINOL as chiral catalyst, thus facilitating their potential applications. Besides, this strategy has been proven to be practical for obtaining flavored allyl alcohols with high enantioselectivity in food analysis and screening insect attractants. Biological evaluation of target molecules is in progress and will be reported in due course.

## Figures and Tables

**Table 1:**
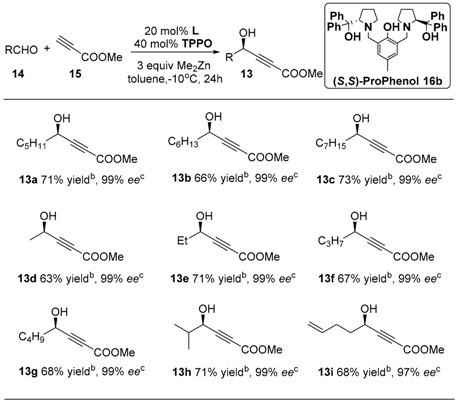
Synthesis of chiral alkynol units **13a**–**e** of C17 polyacetylenes via the asymmetric addition of methyl propiolate to aliphatic aldehydes ^a^.

^a^ Unless otherwise noted, the reaction was carried out on a 1.0 mmol scale in toluene (1.0 mL); ^b^ Isolated yields; ^c^ The *ee* values were determined by chiral HPLC.

**Table 2:**
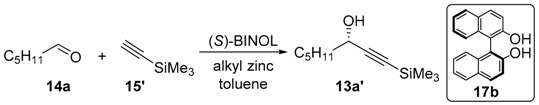
Screening of reaction conditions and ligands of asymmetric addition of **15’** with **14a**.

Entry	Ligand	Ligand/%	Temp/°C	Yield/% ^c^	*ee*/% ^d^
1 ^a^	**16b**	20	−10	70	75
2 ^a^	**16b**	40	−10	72	78
3 ^a^	**16b**	40	25	74	76
4 ^b^	**17b**	40	−10	--	--
5 ^b^	**17b**	20	25	52	70
6 ^b^	**17b**	40	25	71	80
7 ^b^	**17b**	60	25	67	81

^a^ The reaction was carried out on a 0.5 mmol scale in toluene (0.5 mL), the organozinc reagent was Me_2_Zn; ^b^ The reaction was carried out on a 0.5 mmol scale in DCM (6.0 mL), the organozinc reagent was Et_2_Zn, Et_2_Zn:Ti(O*^i^*Pr)_4_:aldehyde = 4:1:1; ^c^ Isolated yields; ^d^ The *ee* values were determined by chiral HPLC.

## Supplementary Materials

The following are available online: Figure S1–S43.
