# Supplementary material for: A General Asymmetric Synthesis of (R)-Matsutakeol and Flavored Analogs
Source: Molecules. 2017 Feb 27;22(3):364. doi: 10.3390/molecules22030364 (PMC6155351; doi:10.3390/molecules22030364)
Supplement: Supplementary file 1 [file molecules-22-00364-s001.pdf]

Supporting information for

A General Asymmetric Synthesis of (*R*)-Matsutakeol and Flavored Analogs

Jia Liu [1], Honglian Li [1], Chao Zheng [2], Shichao Lu [1], Xianru Guo [1], Xinming Yin [1], Risong Na [1,4], Bin Yu [3]* and Min Wang [4]*

Address:

[1]Collaborative Innovation Center of Henan Grain Crops, National Key Laboratory of Wheat and Maize Crop Science, College of Plant Protection, Henan Agricultural University, Wenhua Road NO. 95, Zhengzhou 450002, China.

[2] Key Lab of Tropical Medicinal Plant Chemistry of Hainan Province, School of Chemistry and Chemical Engineering, Hainan Normal University, Haikou 57115, China.

[3]School of Pharmaceutical Sciences, Zhengzhou University, Zhengzhou 450001, China.

[4]School of Sciences, China Agricultural University, Beijing 100193, China.

Email:

Risong Na - narisong@outlook.com, nrs@henau.edu.cn;*

Bin Yu - zzuyubin@hotmail.com*

** Corresponding author*

Contents

1. ^{1}H and ^{13}C NMR spectra S1- S22

2. HPLC chromatography of the chiral products S23- S43

2. ^{1}H and ^{13}C NMR spectras of the products

(Compound 13a). (*R*)-methyl-4-hydroxynon-2-ynoate: ^{1}H NMR

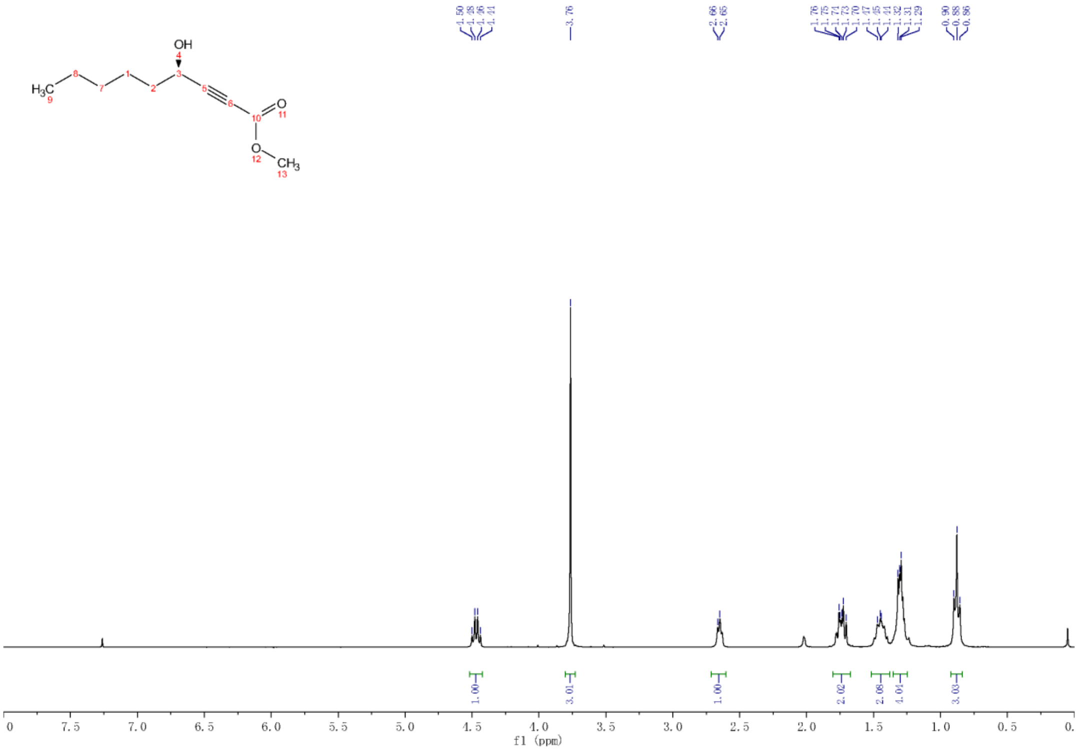

(Compound 13a). (*R*)-methyl-4-hydroxynon-2-ynoate: ^{13}C NMR

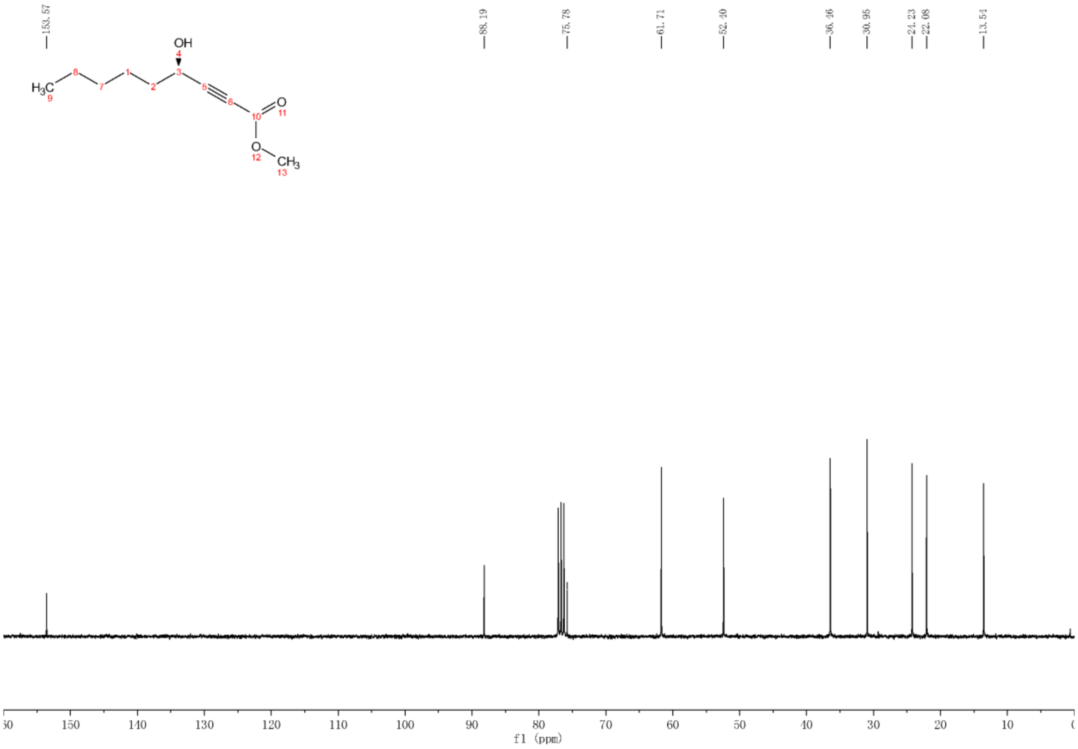

(Compound 13b). (*R*)-methyl-4-hydroxydec-2-ynoate: ^{1}H NMR

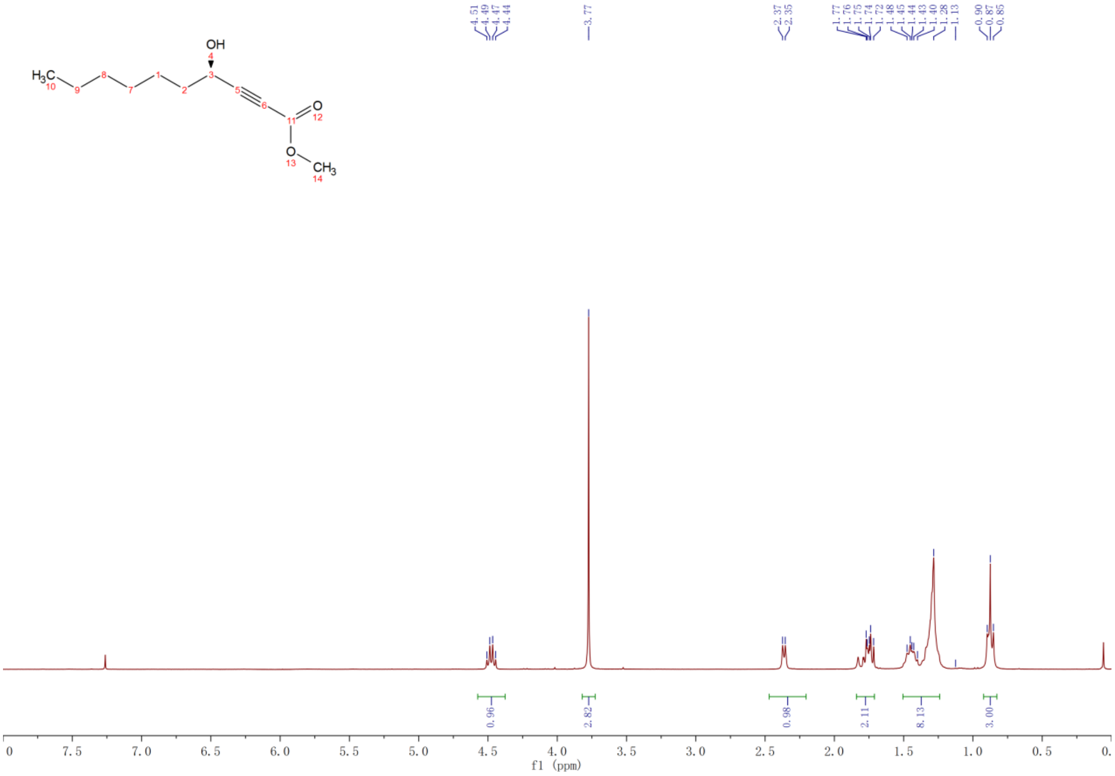

(Compound 13b). (*R*)-methyl-4-hydroxydec-2-ynoate: ^{13}C NMR

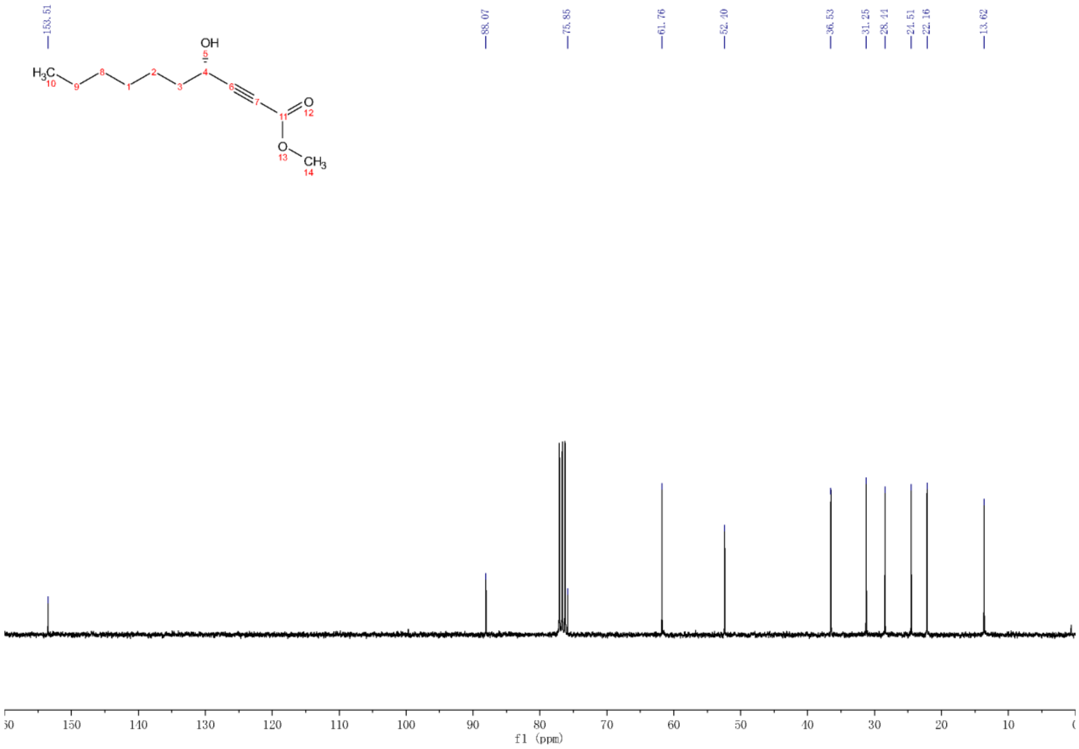

(Compound 13c). (*R*)-methyl-4-hydroxyundec-2-ynoate: ^{1}H NMR

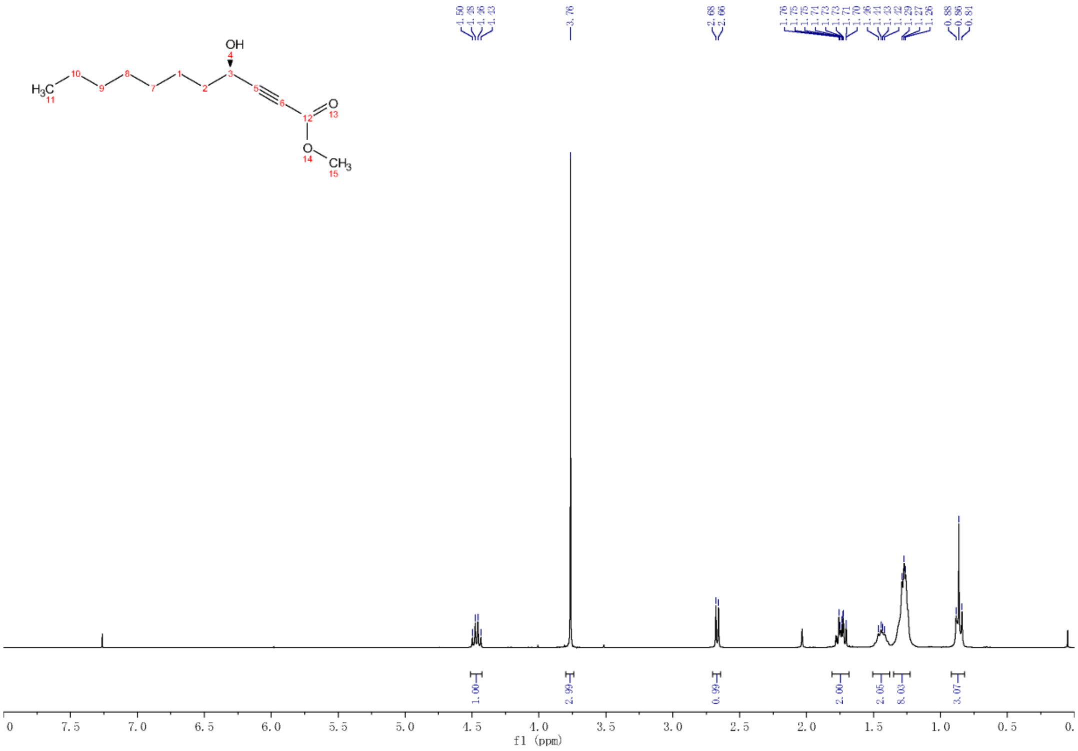

(Compound 13c). (*R*)-methyl-4-hydroxyundec-2-ynoate : ^{13}C NMR:

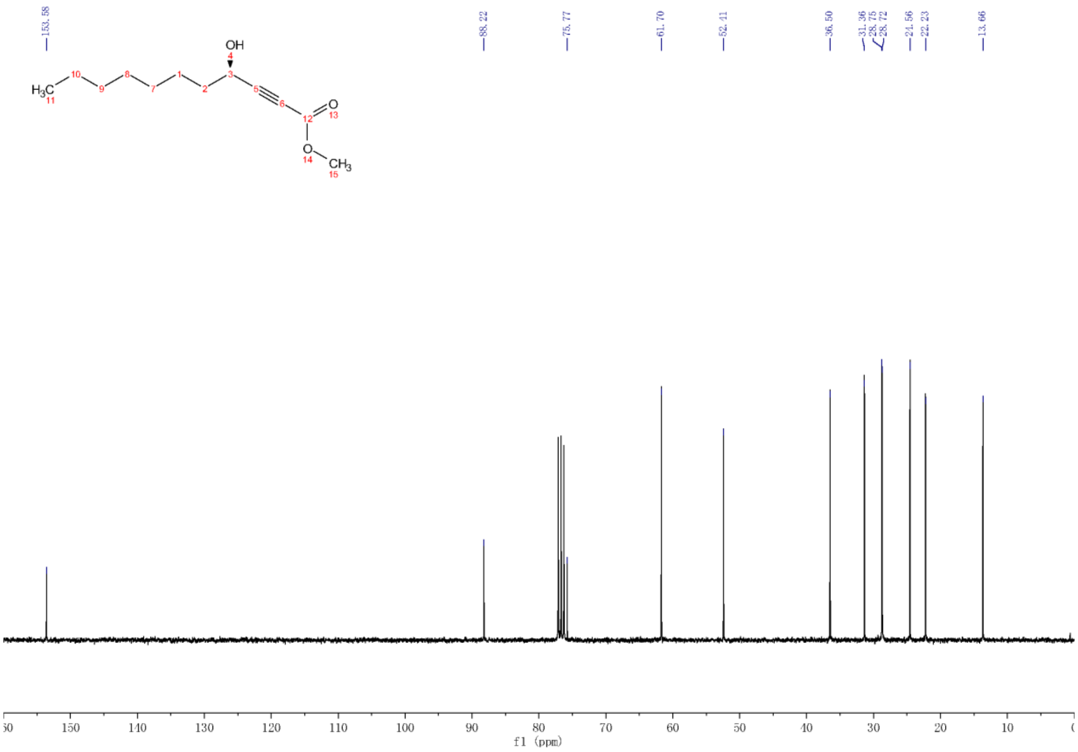

(Compound 13d). (*R*)-methyl-4-hydroxypent-2-ynoate: ^{1}H NMR

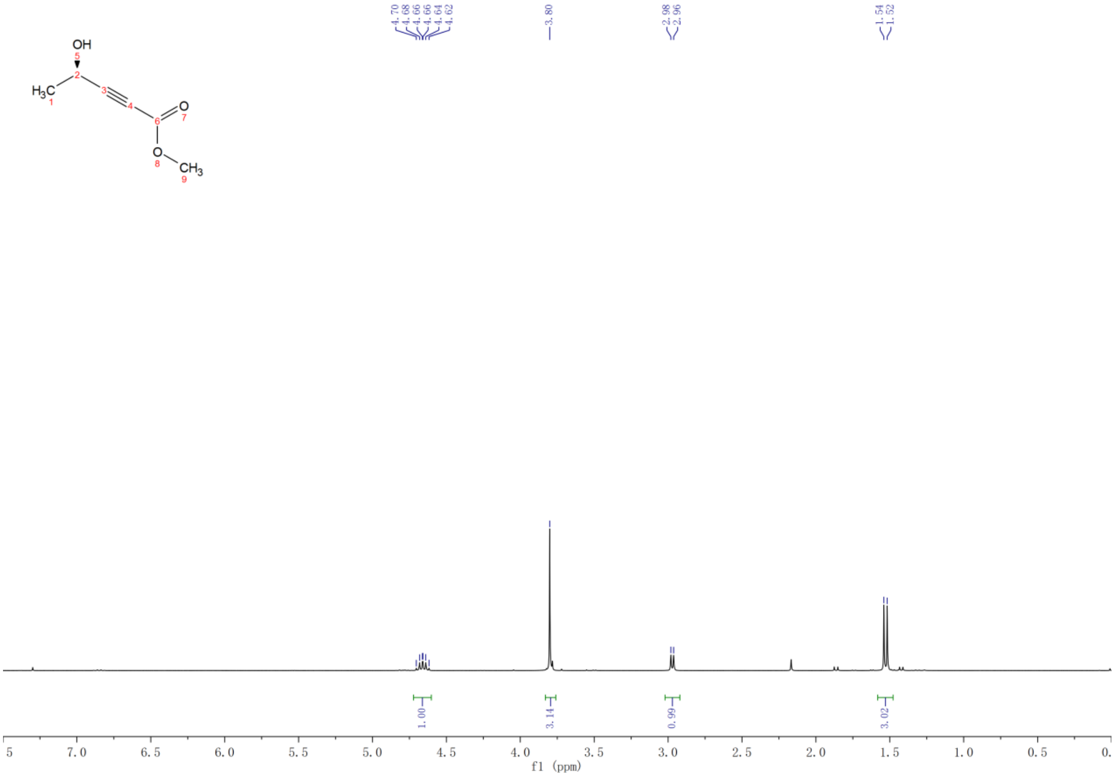

(Compound 13d). (*R*)-methyl-4-hydroxypent-2-ynoate: ^{13}C NMR

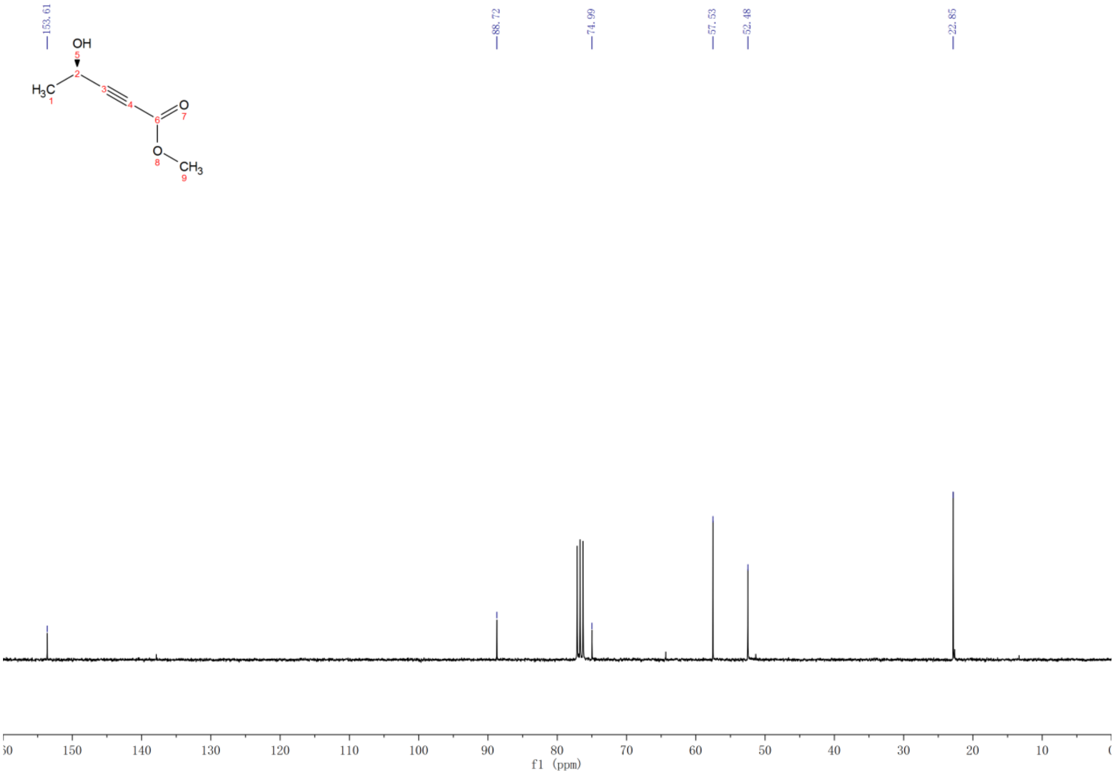

(Compound 13e). (*R*)-methyl-4-hydroxyhex-2-ynoate: ^{1}H NMR

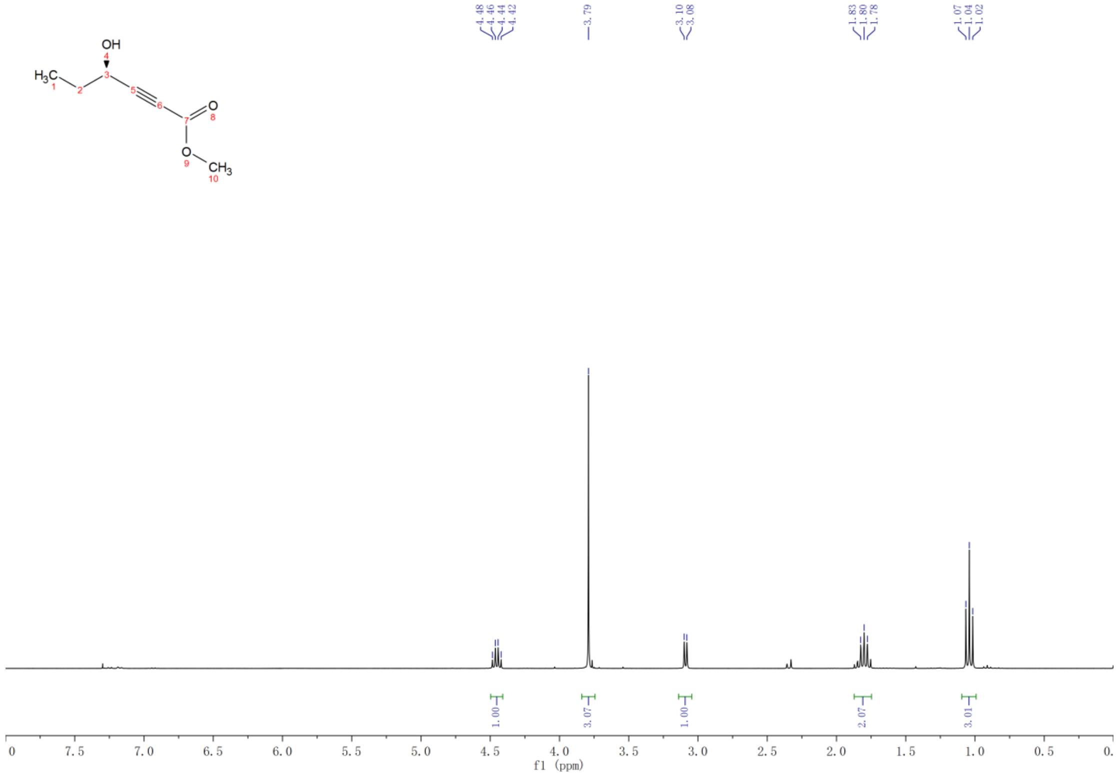

(Compound 13e). (*R*)-methyl-4-hydroxyhex-2-ynoate: ^{13}C NMR

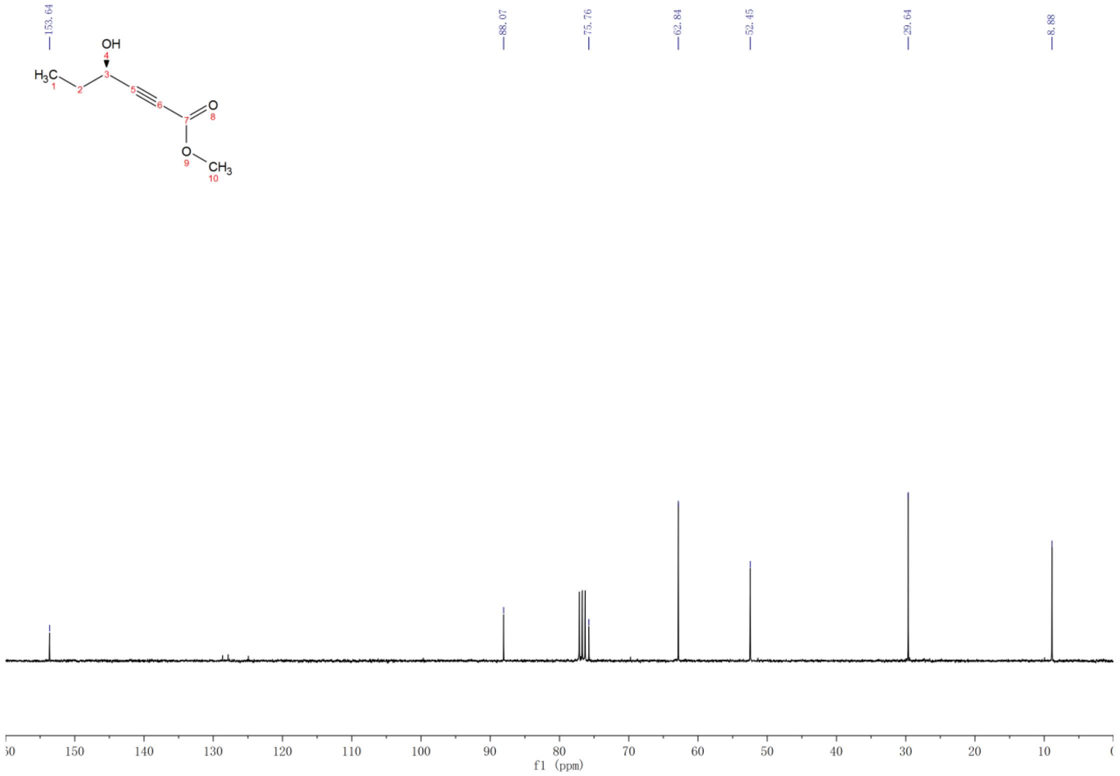

(Compound 13f). (*R*)-methyl-4-hydroxyhept-2-ynoate: ^{1}H NMR

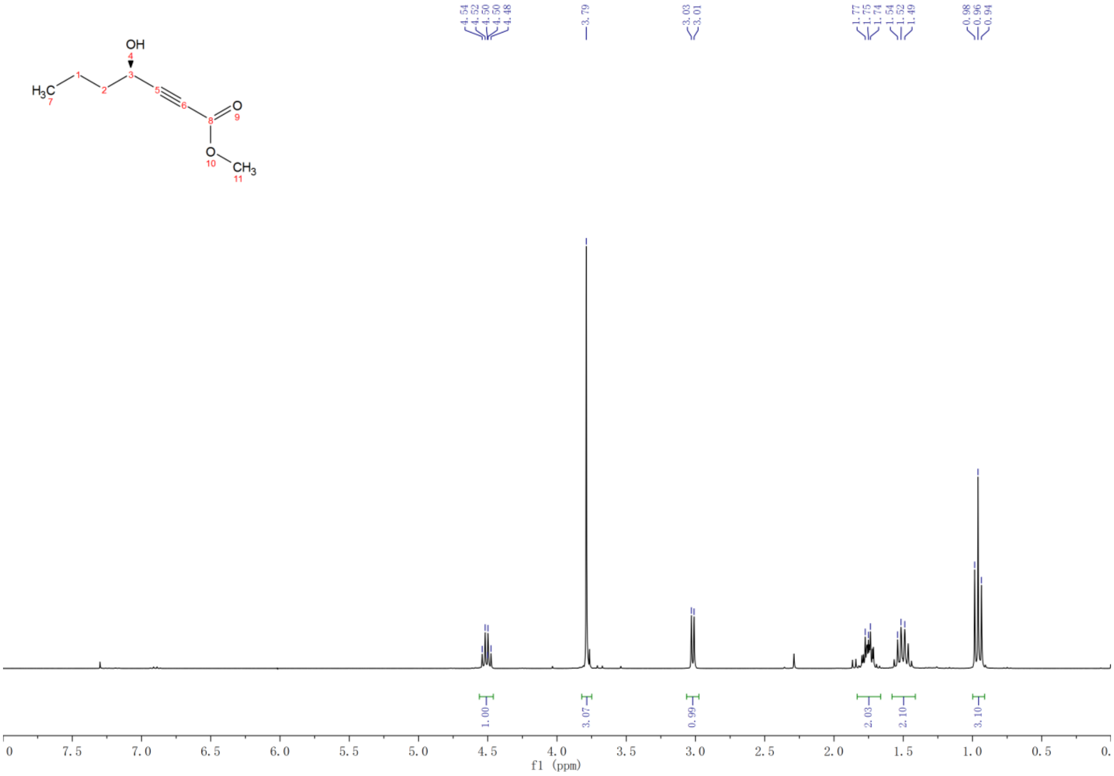

(Compound 13f). (*R*)-methyl-4-hydroxyhept-2-ynoate: ^{13}C NMR

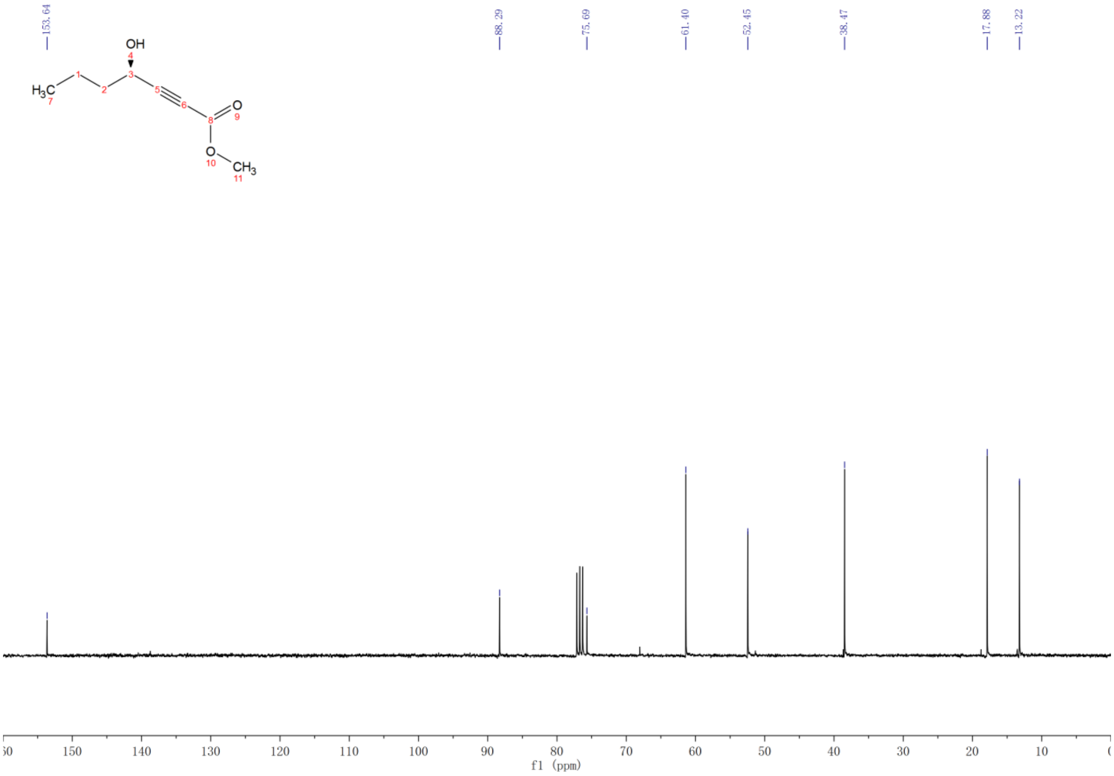

(Compound 13g). (*R*)-methyl-4-hydroxyoct-2-ynoate: ^{1}H NMR

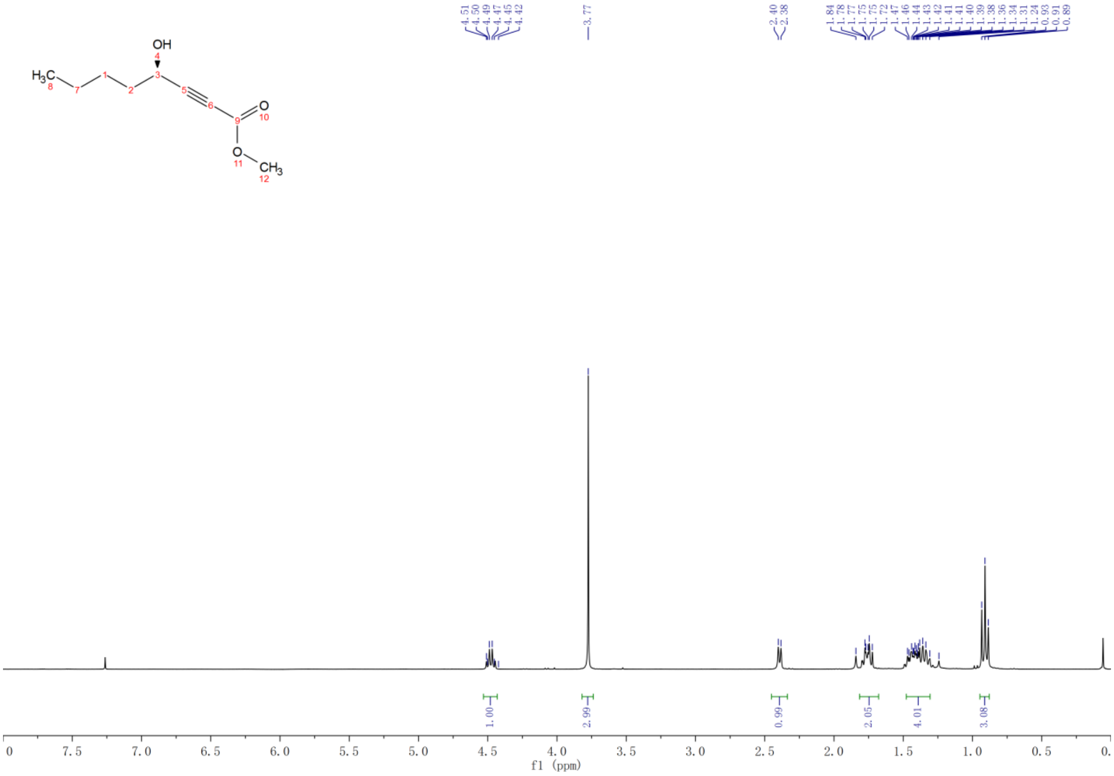

(Compound 13g). (*R*)-methyl-4-hydroxyoct-2-ynoate: ^{13}C NMR

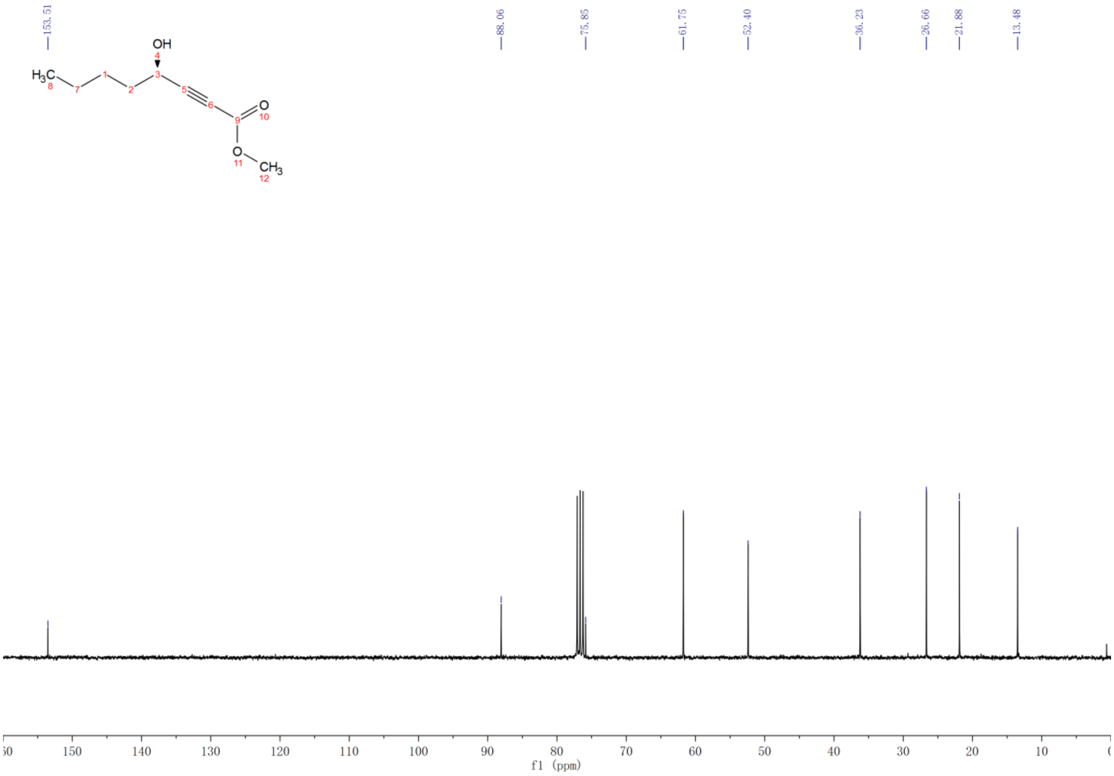

(Compound 13h). (*R*)-methyl-4-hydroxy-5-methylhex-2-ynoate: ^{1}H NMR

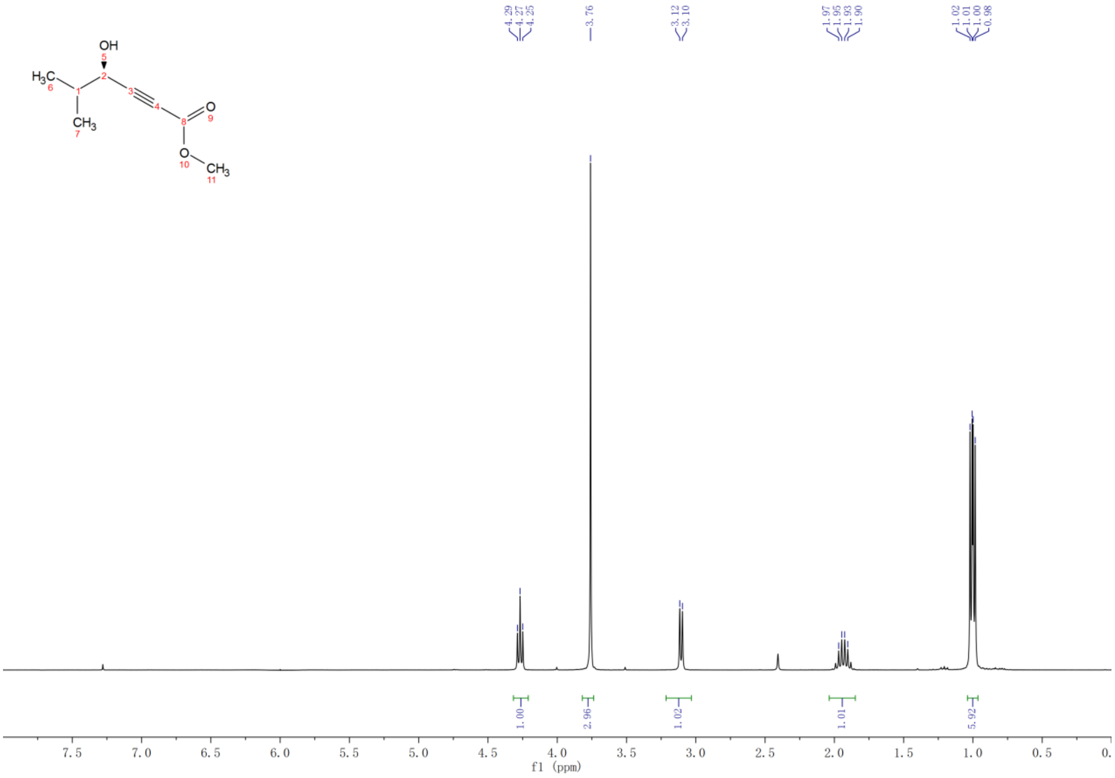

(Compound 13h). (*R*)-methyl-4-hydroxy-5-methylhex-2-ynoate: ^{13}C NMR

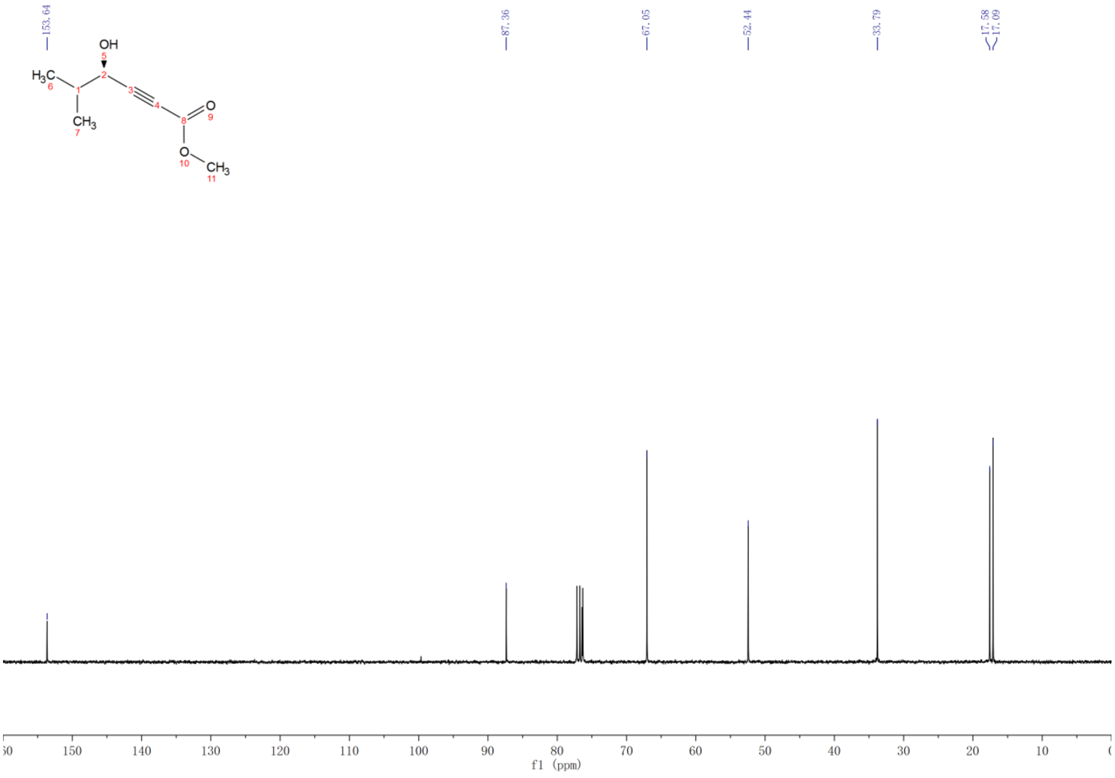

(Compound 13i). (*R*)-methyl-4-hydroxyoct-7-en-2-ynoate: ^{1}H NMR

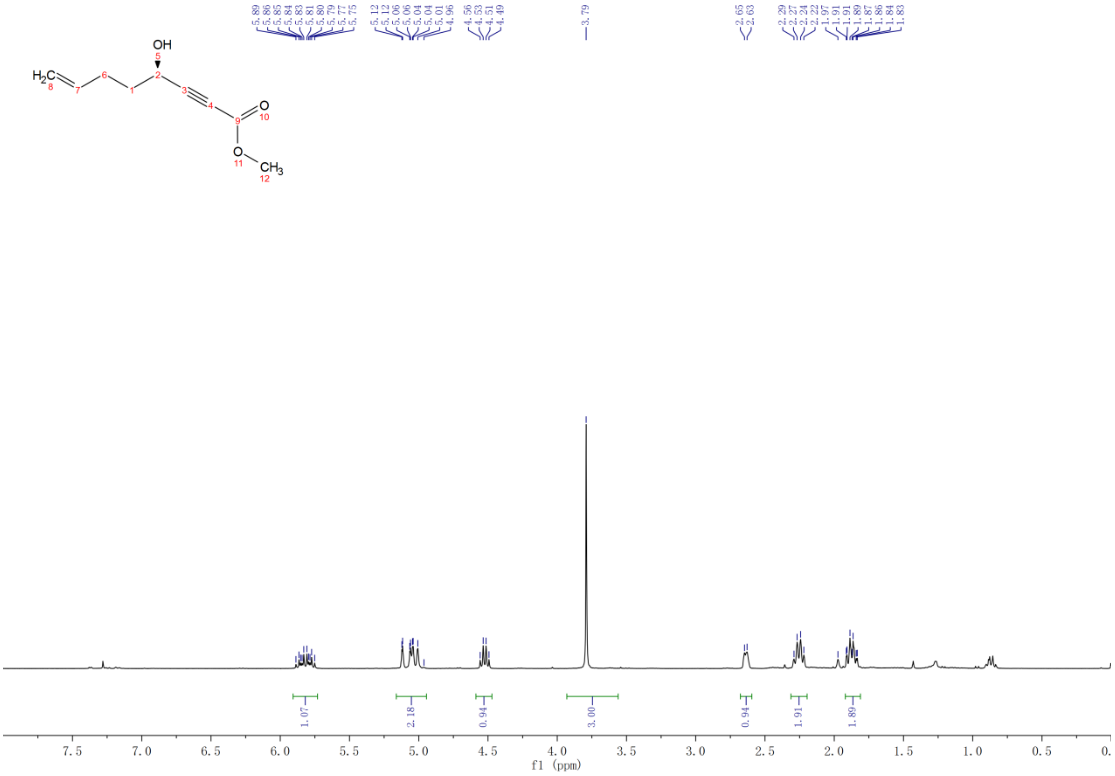

(Compound 13i). (*R*)-methyl-4-hydroxyoct-7-en-2-ynoate: ^{13}C NMR

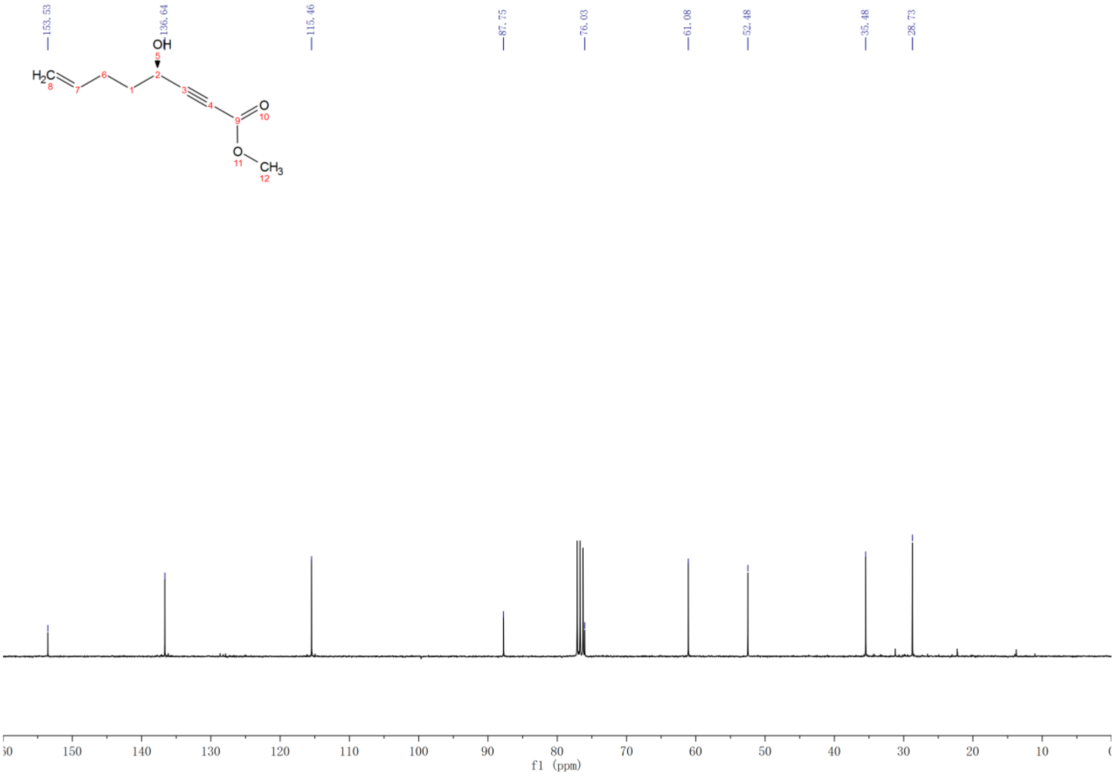

(Compound 13a'). (*R*)-1-(trimethylsilyl)oct-1-yn-3-ol: ^{1}H NMR

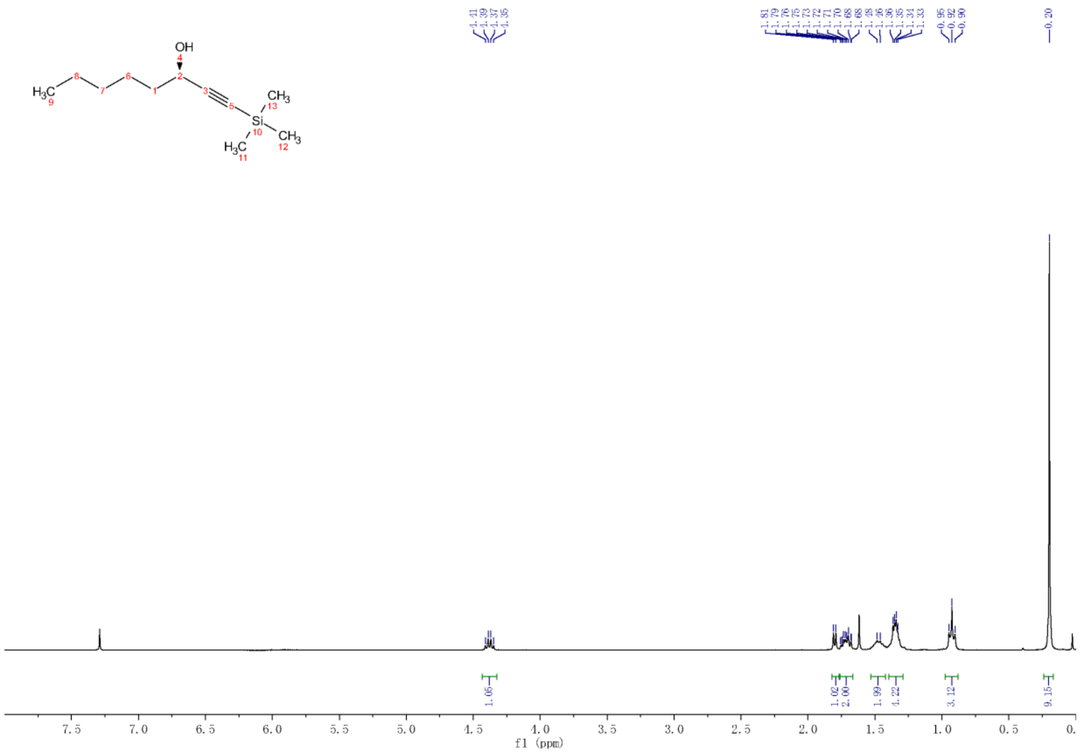

(Compound 13a'). (*R*)-1-(trimethylsilyl)oct-1-yn-3-ol: ^{13}C NMR

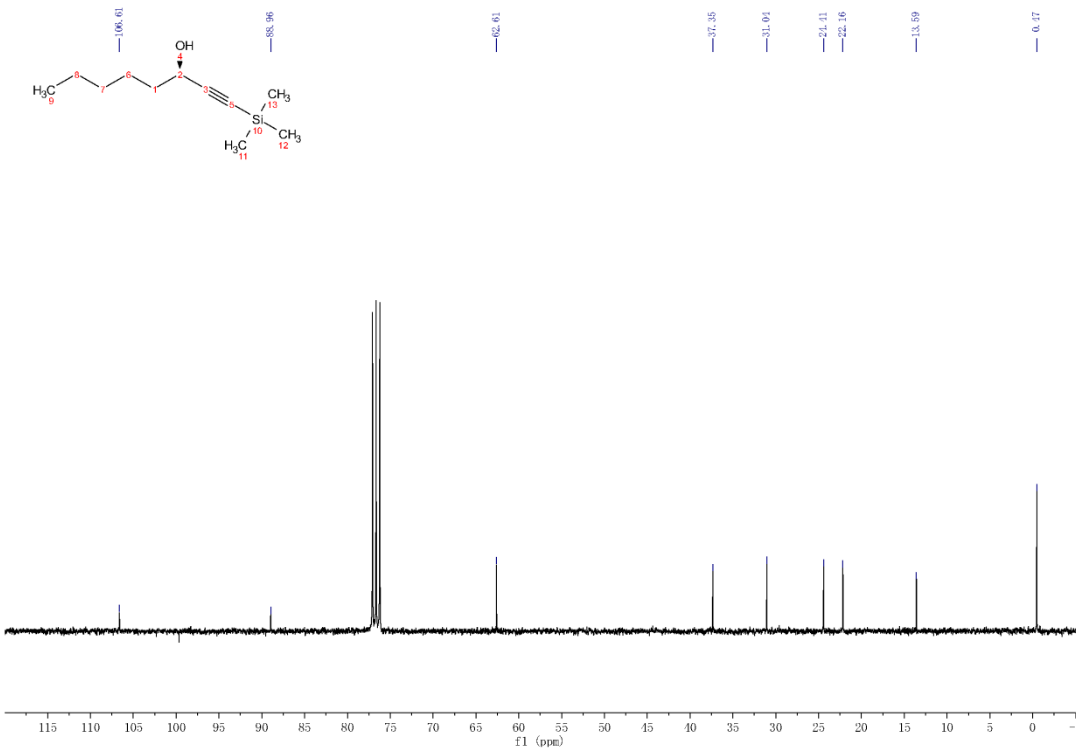

(Compound 13b’). (*R*)-1-(trimethylsilyl)non-1-yn-3-ol: ^{1}H NMR

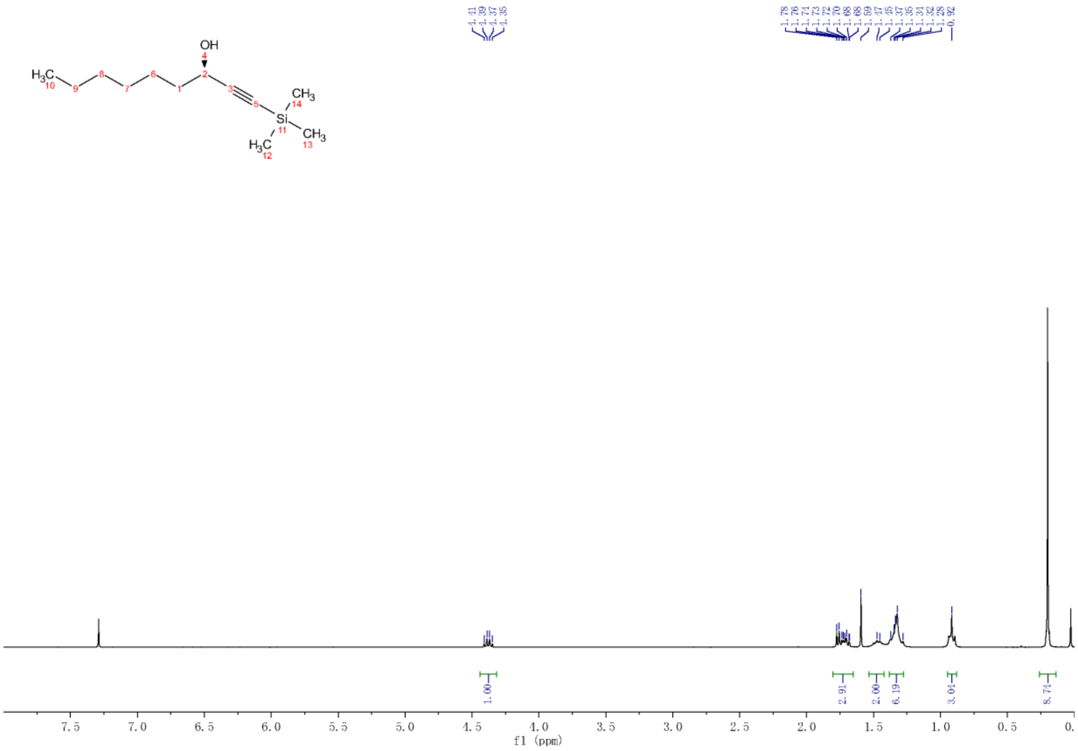

(Compound 13b’). (*R*)-1-(trimethylsilyl)non-1-yn-3-ol: ^{13}C NMR

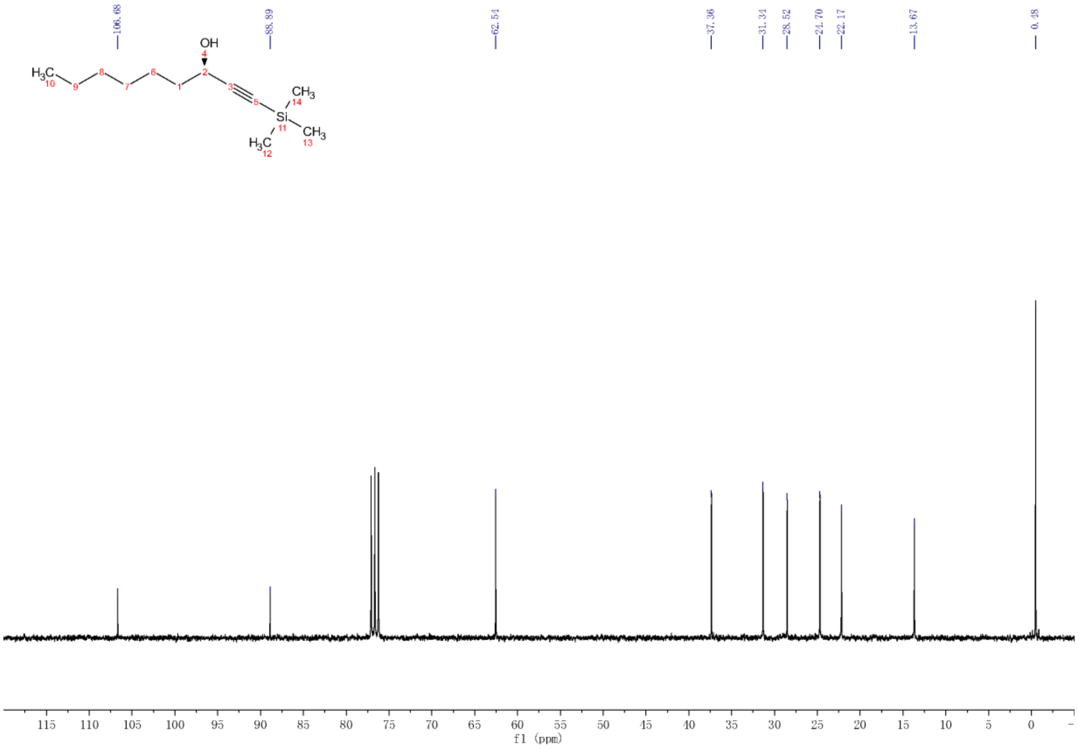

(Compound 13c'). (*R*)-1-(trimethylsilyl)dec-1-yn-3-ol: ^{1}H NMR

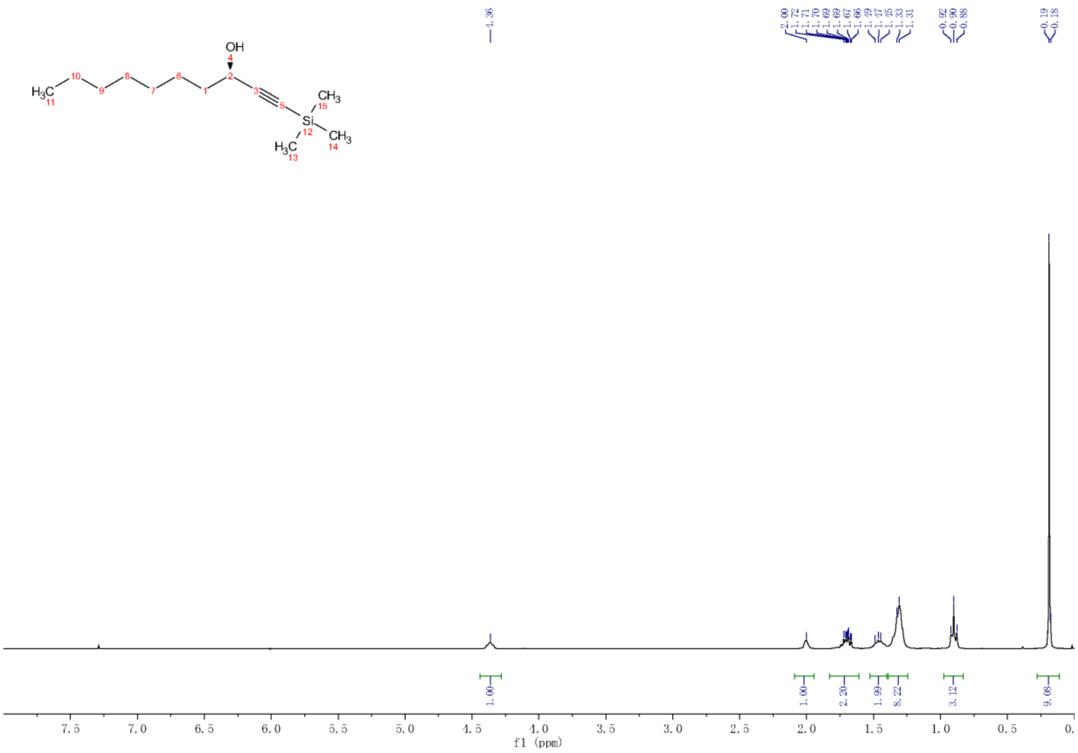

(Compound 13c'). (*R*)-1-(trimethylsilyl)dec-1-yn-3-ol: ^{13}C NMR

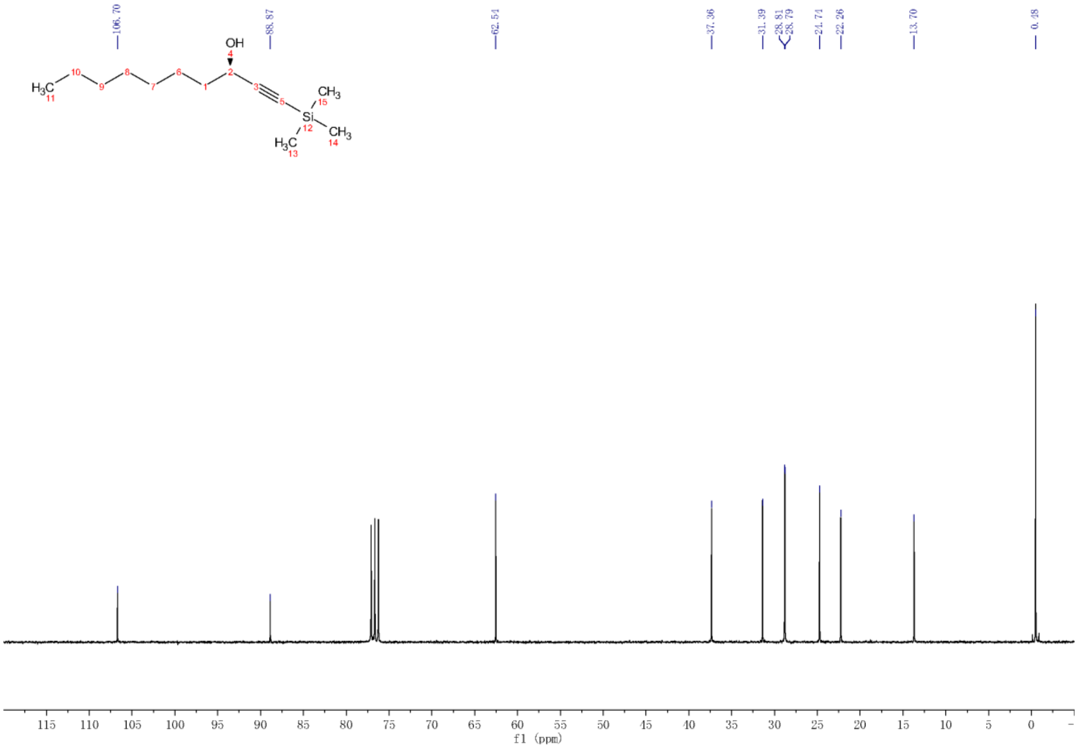

(Compound 12a). (*R*)-oct-1-yn-3-ol: ^{1}H NMR

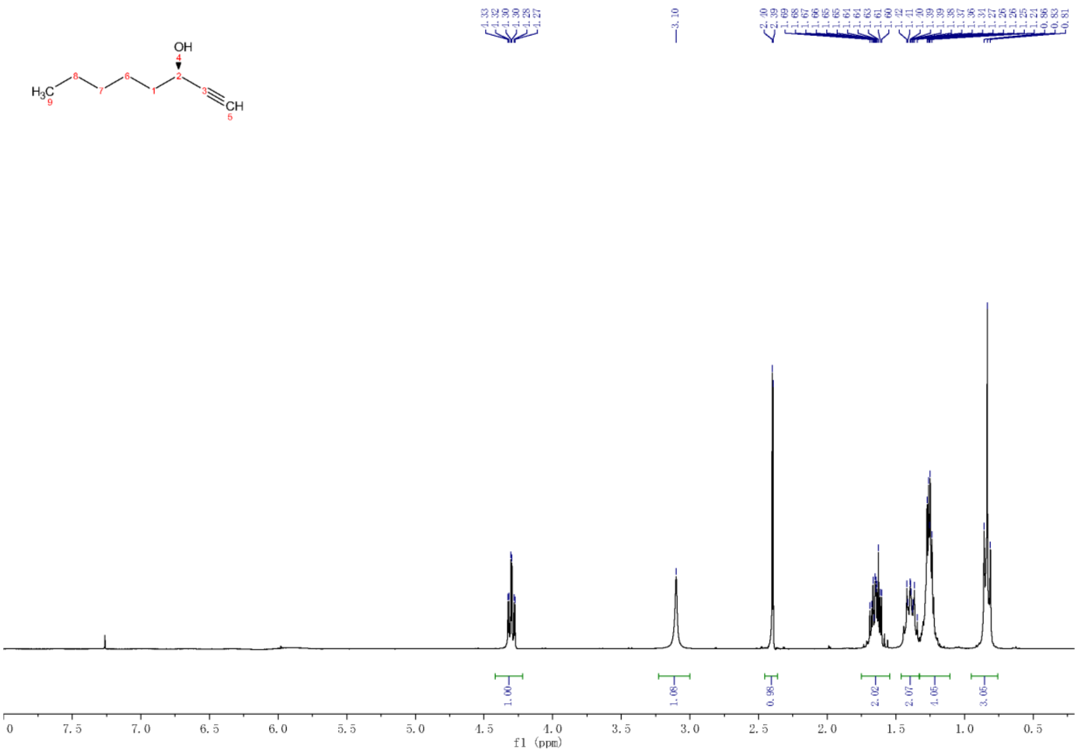

(Compound 12a). (*R*)-oct-1-yn-3-ol: ^{13}C NMR

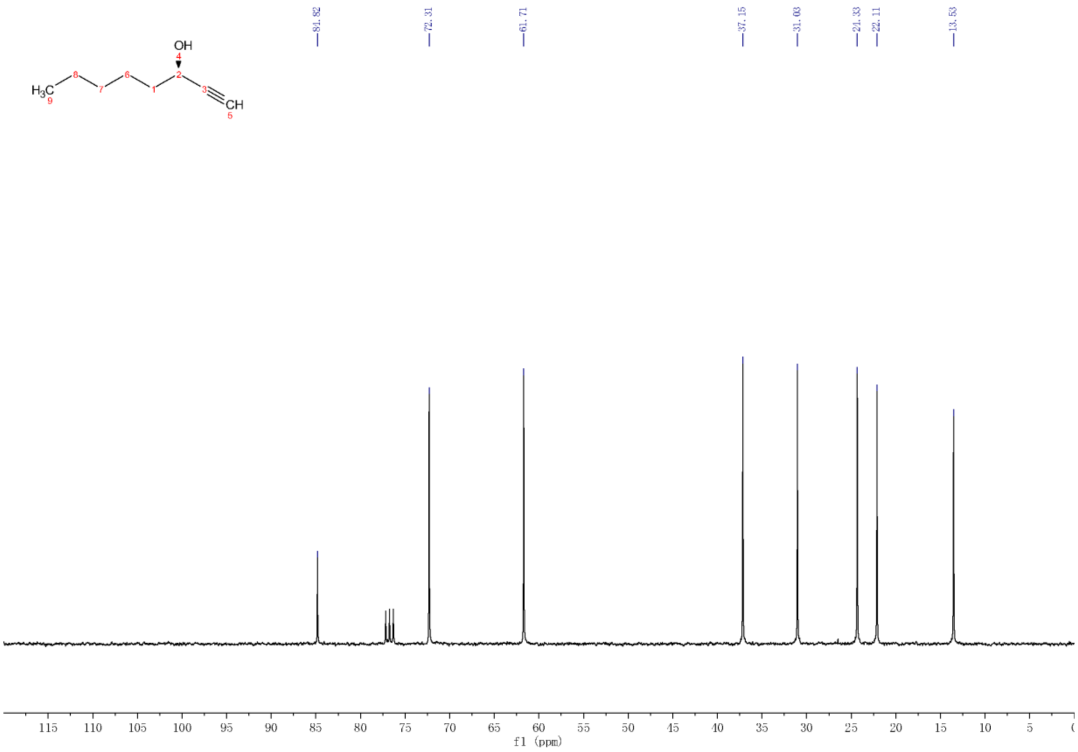

(Compound 12b). (*R*)-1-(trimethylsilyl)oct-1-yn-3-ol: ^{1}H NMR

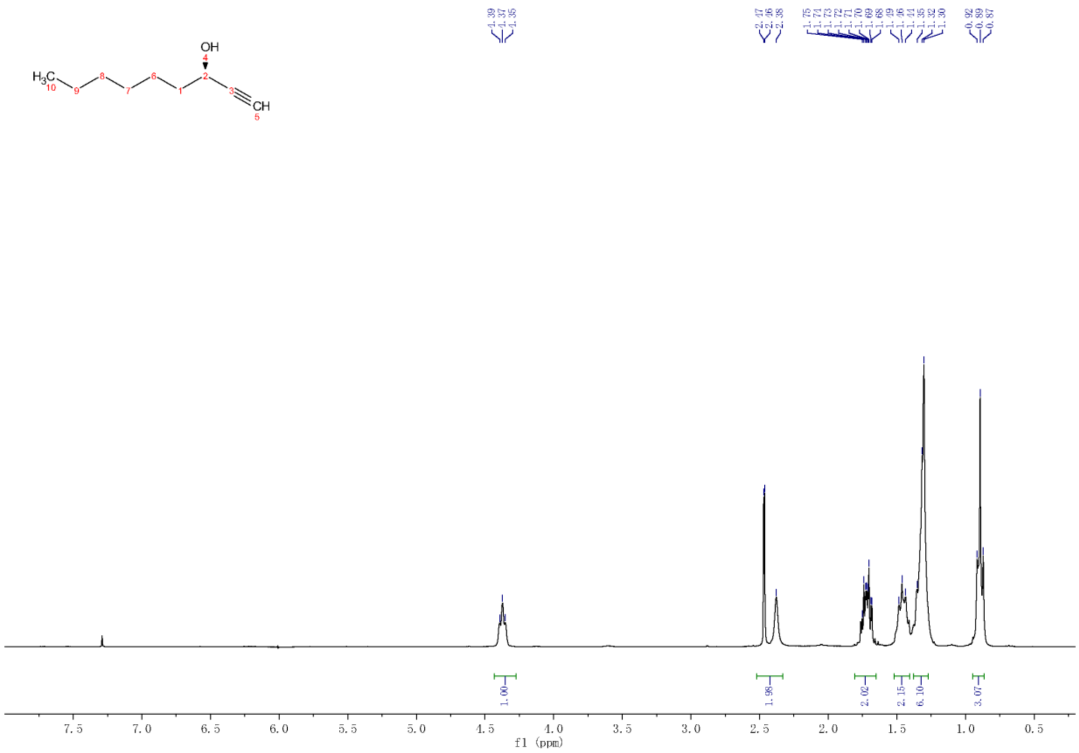

(Compound 12b). (*R*)-1-(trimethylsilyl)oct-1-yn-3-ol: ^{13}C NMR

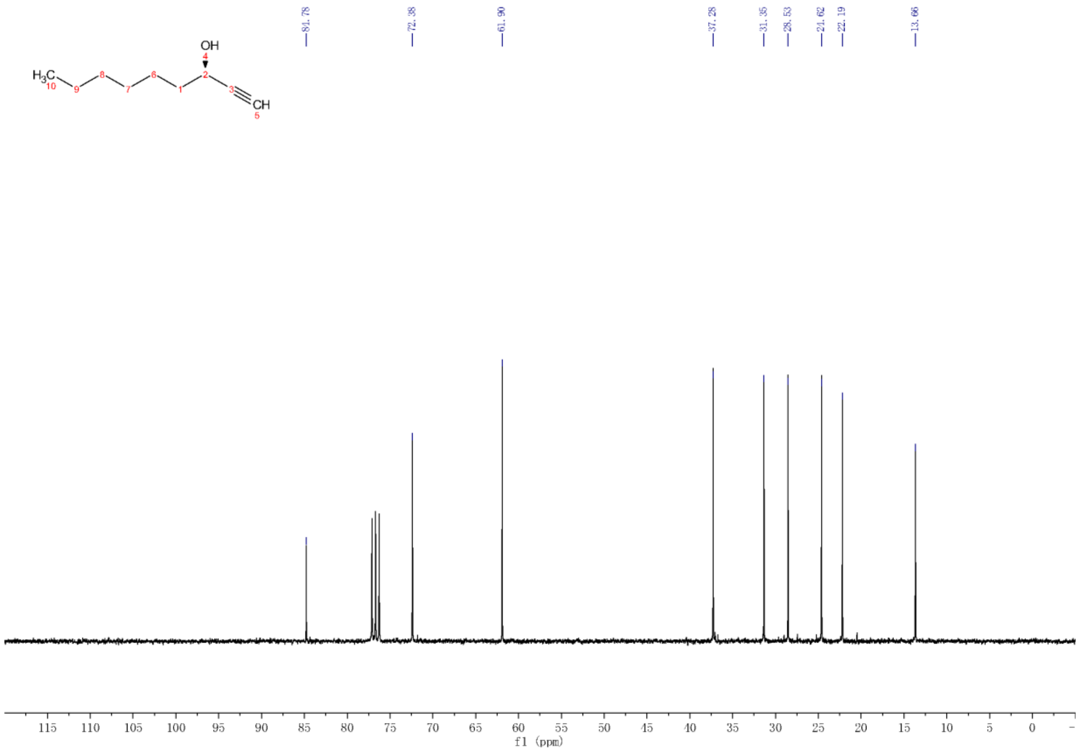

(Compound 12c). (*R*)-dec-1-yn-3-ol: ^{1}H NMR

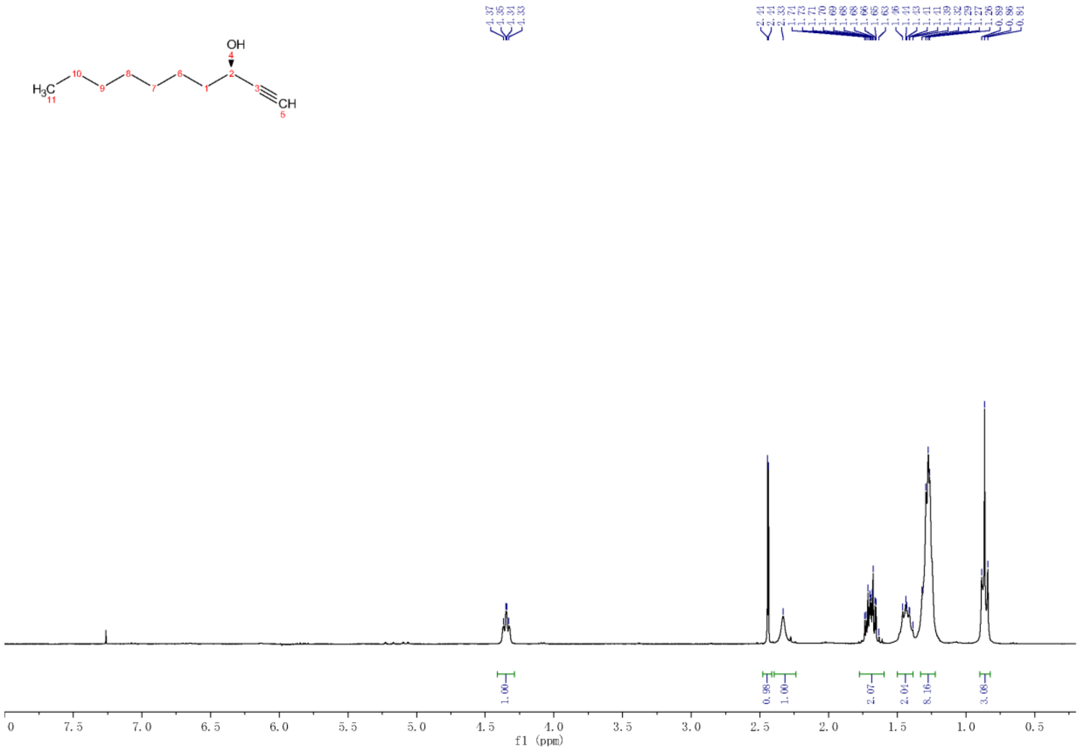

(Compound 12c). (*R*)-dec-1-yn-3-ol: ^{13}C NMR

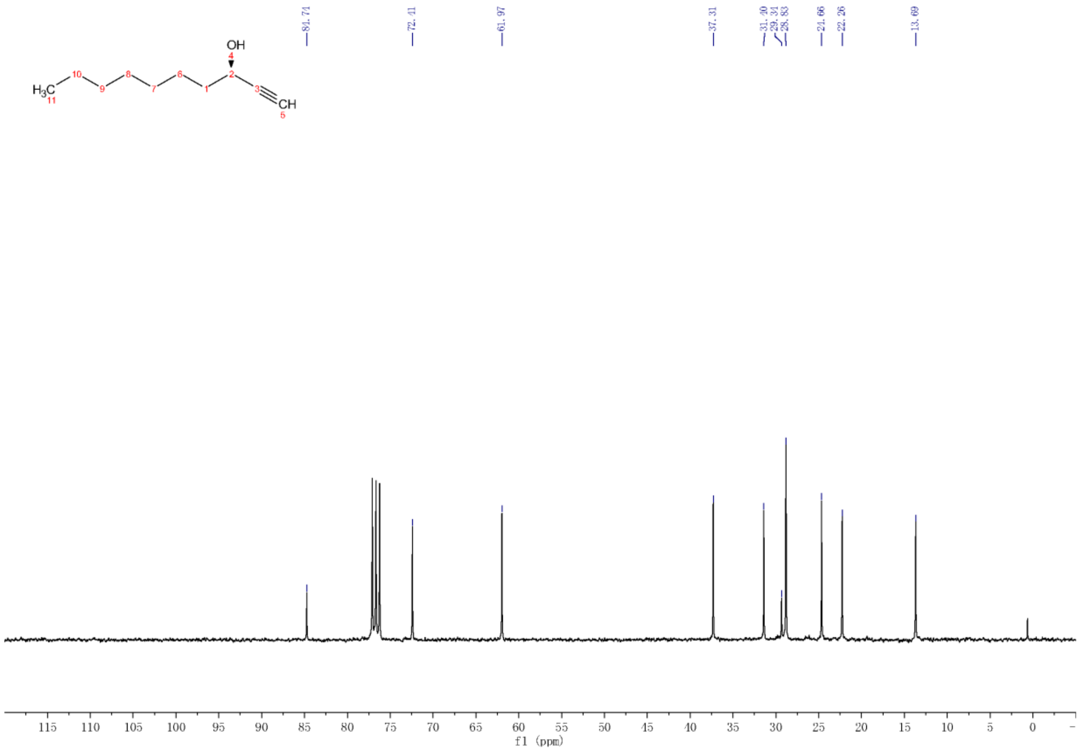

(Compound 6a). (*R*)-oct-1-en-3-ol: ^{1}H NMR

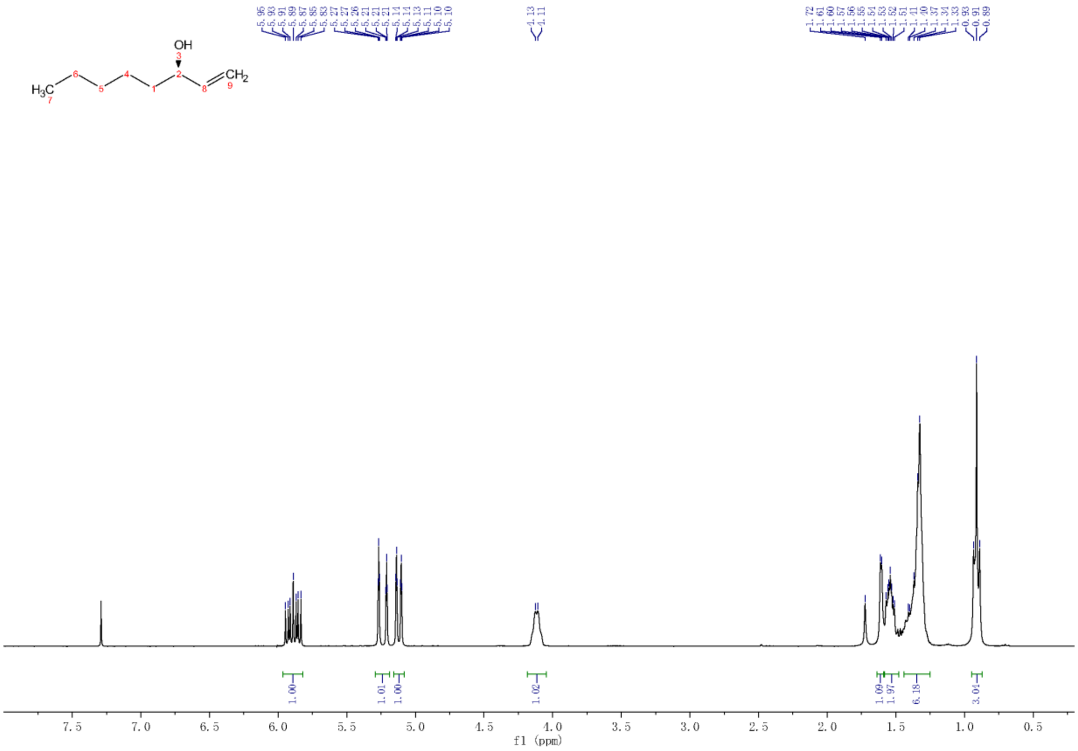

(Compound 6a). (*R*)-oct-1-en-3-ol: ^{13}C NMR

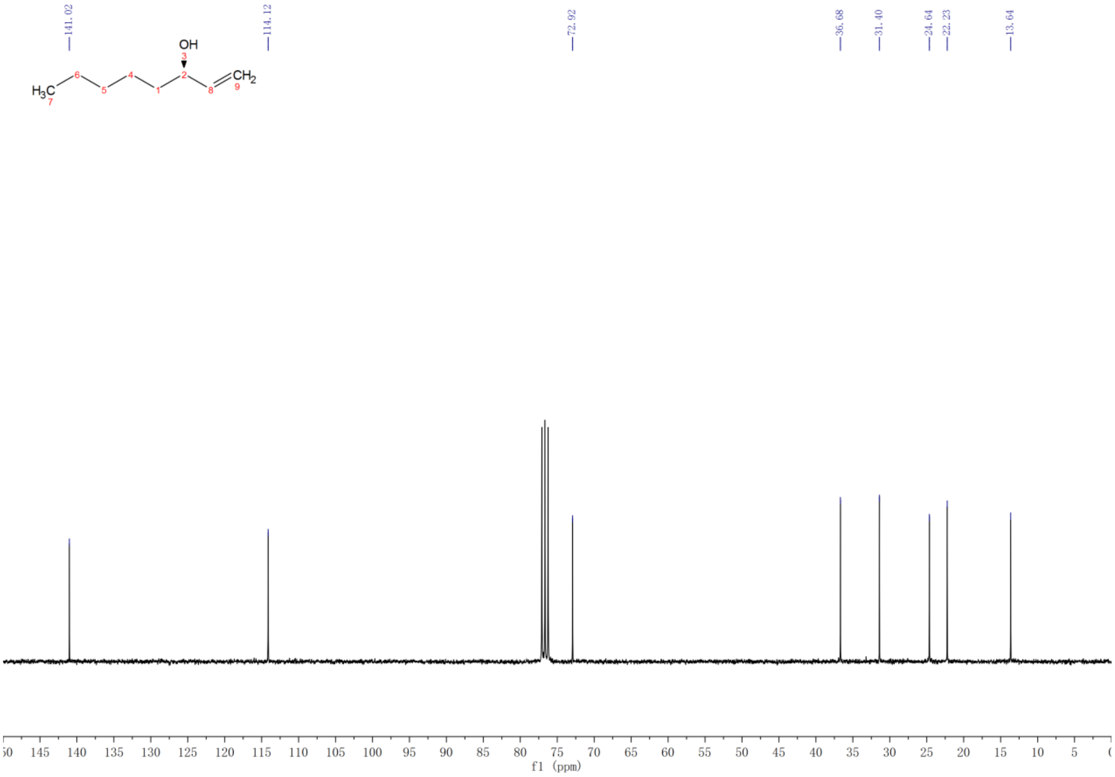

(Compound 6b). (*R*)-non-1-en-3-ol: ^{1}H NMR

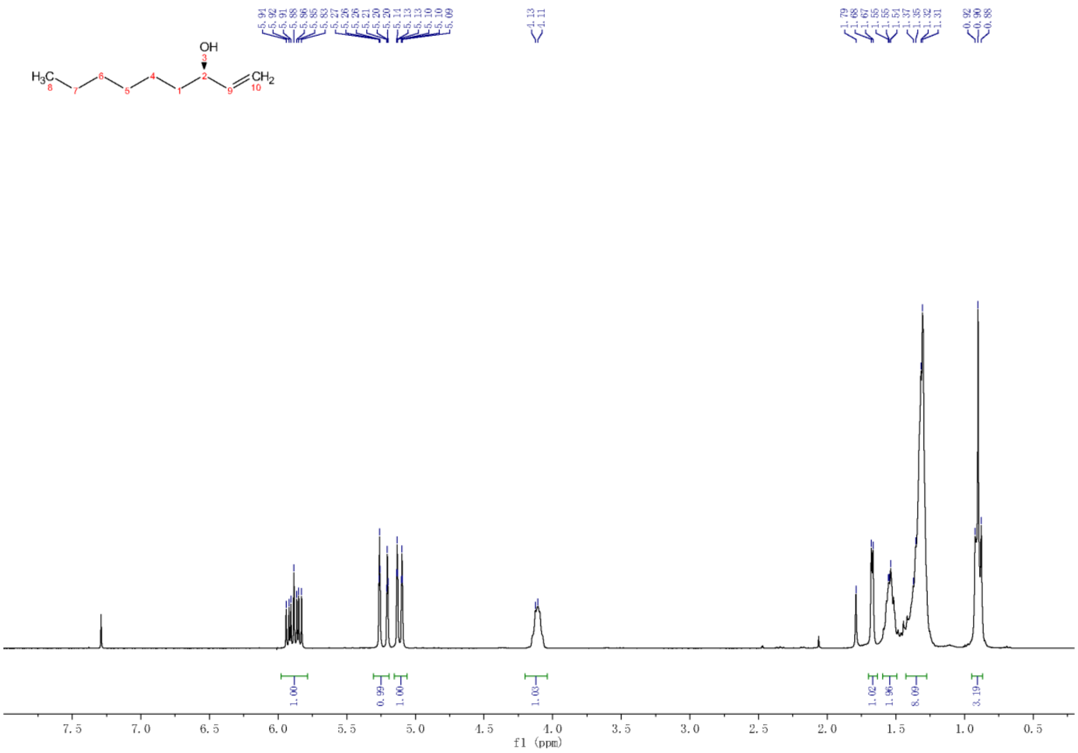

(Compound 6b). (*R*)-non-1-en-3-ol: ^{13}C NMR

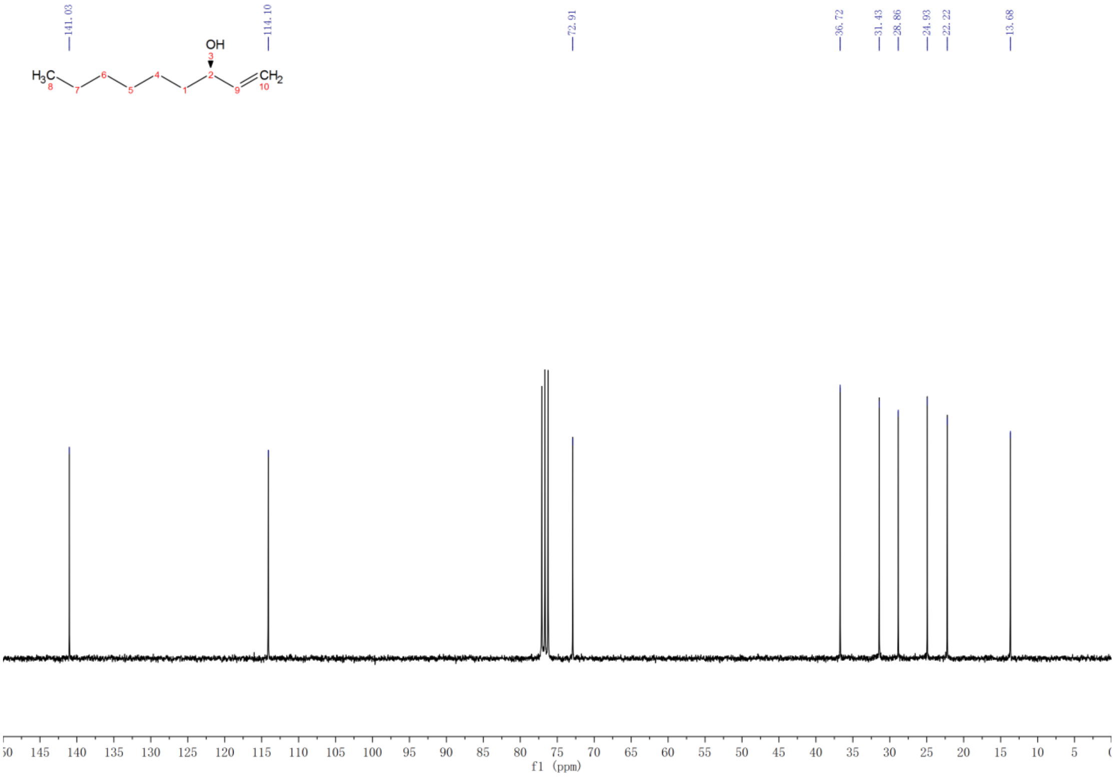

(Compound 6c). (*R*)-dec-1-en-3-ol: ^{1}H NMR

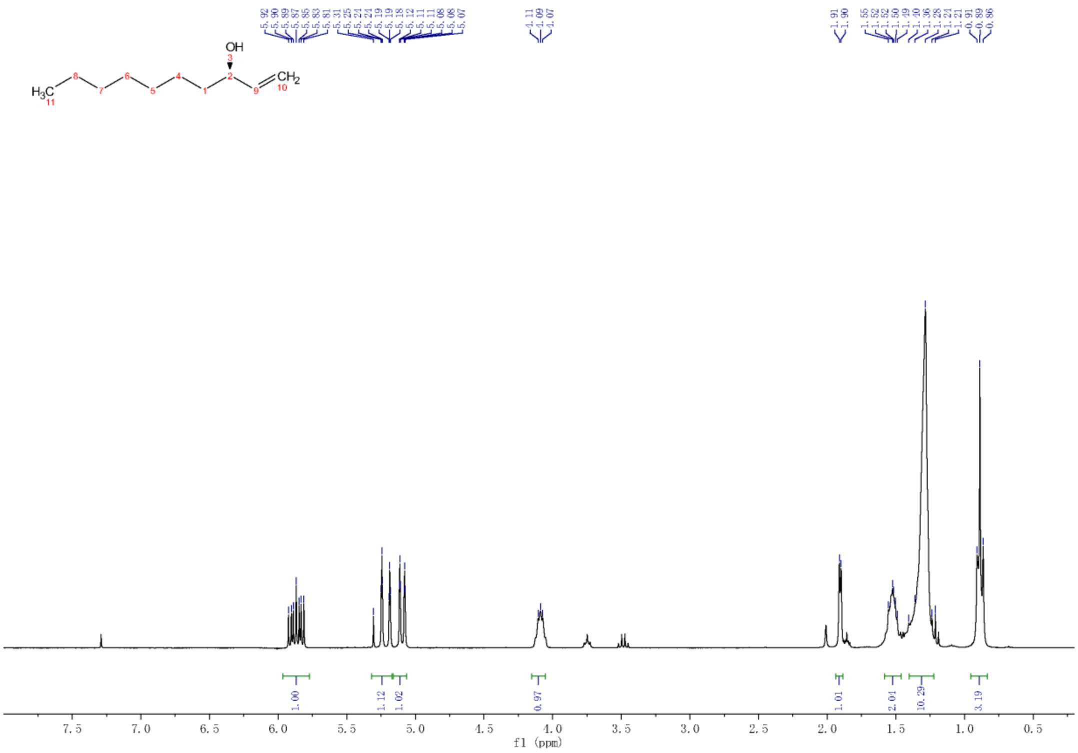

(Compound 6c). (*R*)-dec-1-en-3-ol: ^{13}C NMR

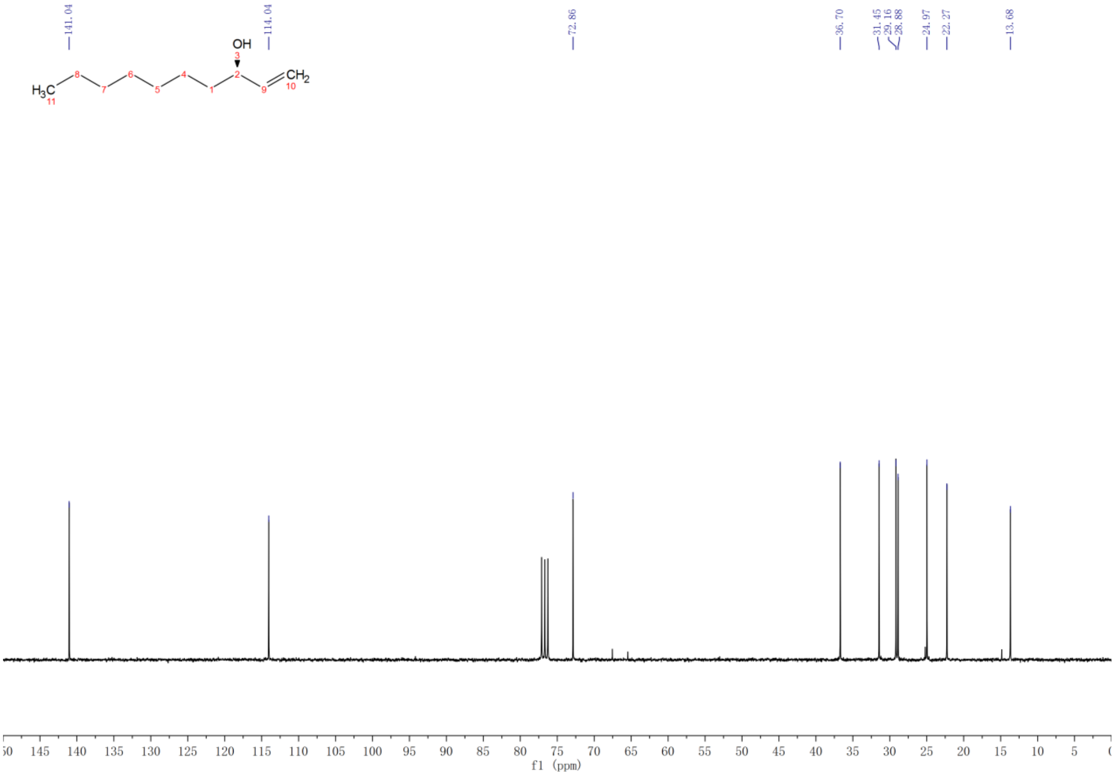

(Compound 21a). (*R*)-oct-1-en-3-yl 3,5-dinitrobenzoate: ^{1}H NMR

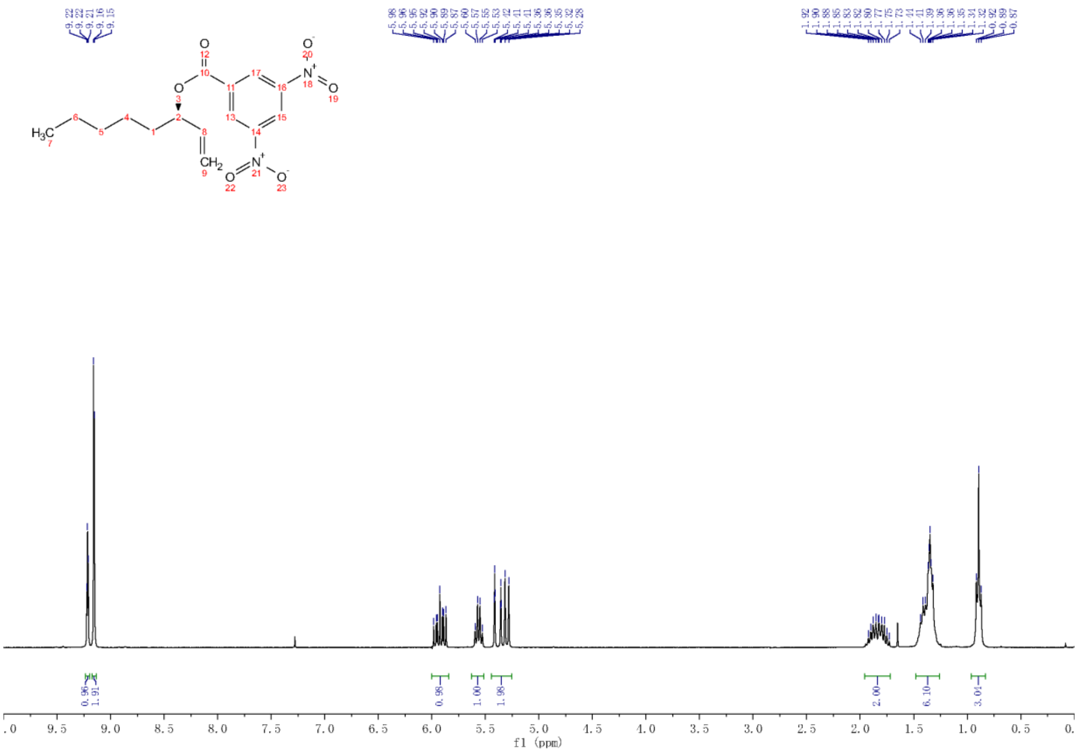

(Compound 21a). (*R*)-oct-1-en-3-yl 3,5-dinitrobenzoate: ^{13}C NMR

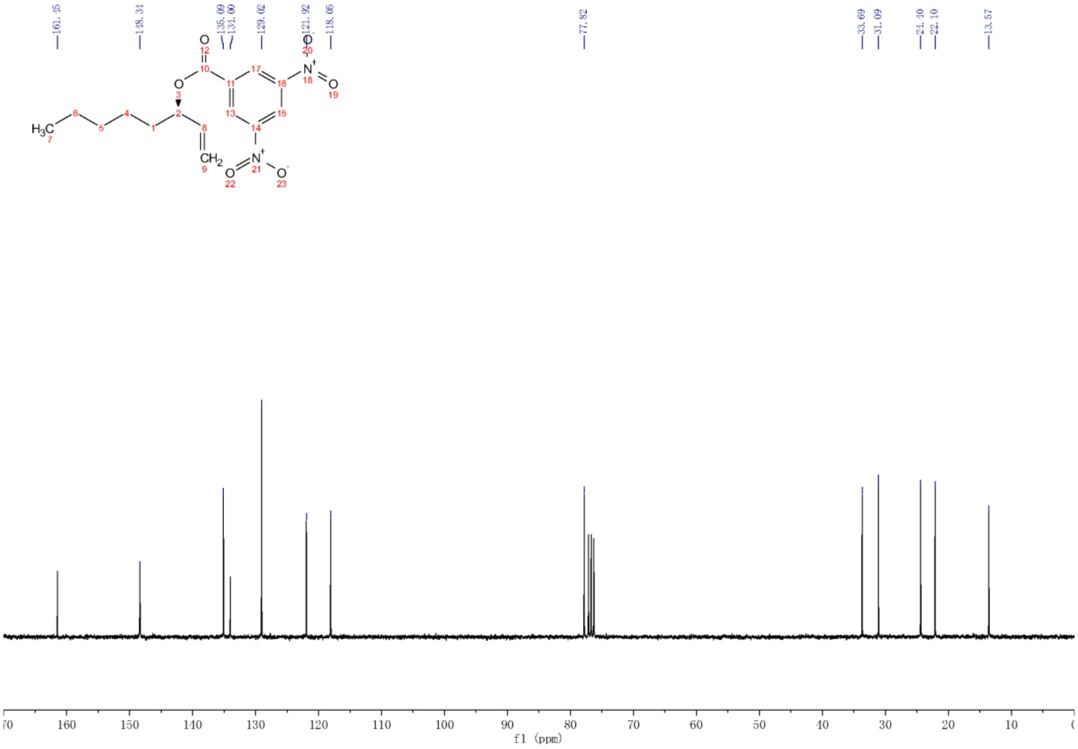

(Compound 21b). (*R*)-non-1-en-3-yl 3,5-dinitrobenzoate: ^{1}H NMR

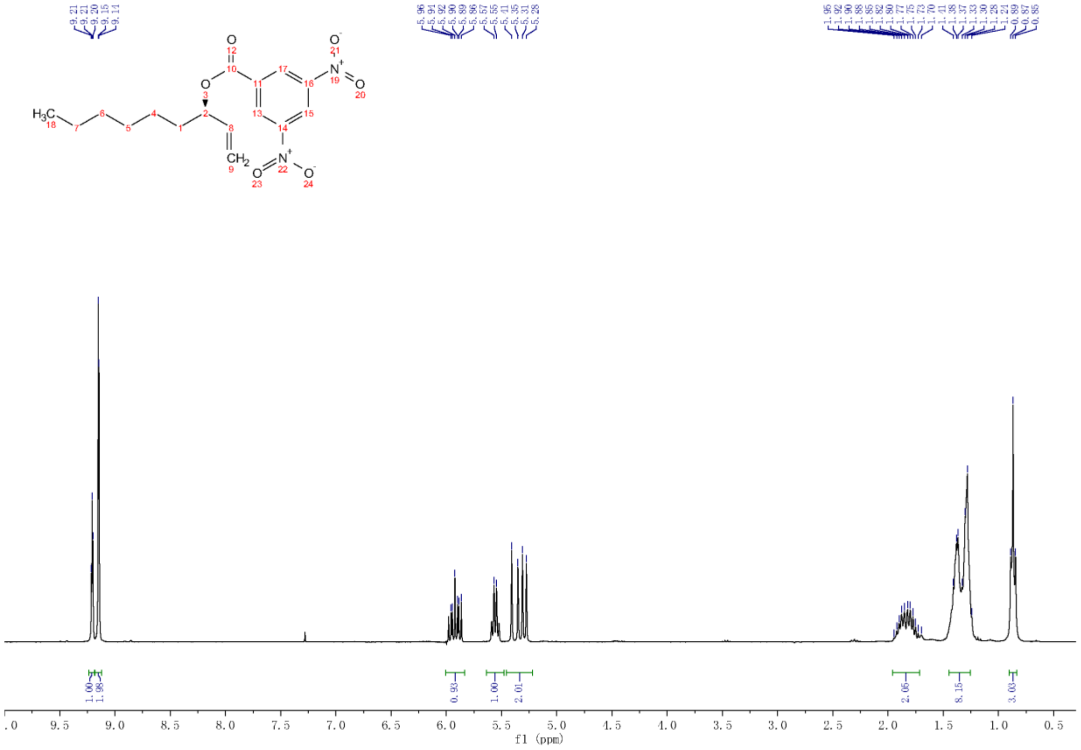

(Compound 21b). (*R*)-non-1-en-3-yl 3,5-dinitrobenzoate: ^{13}C NMR

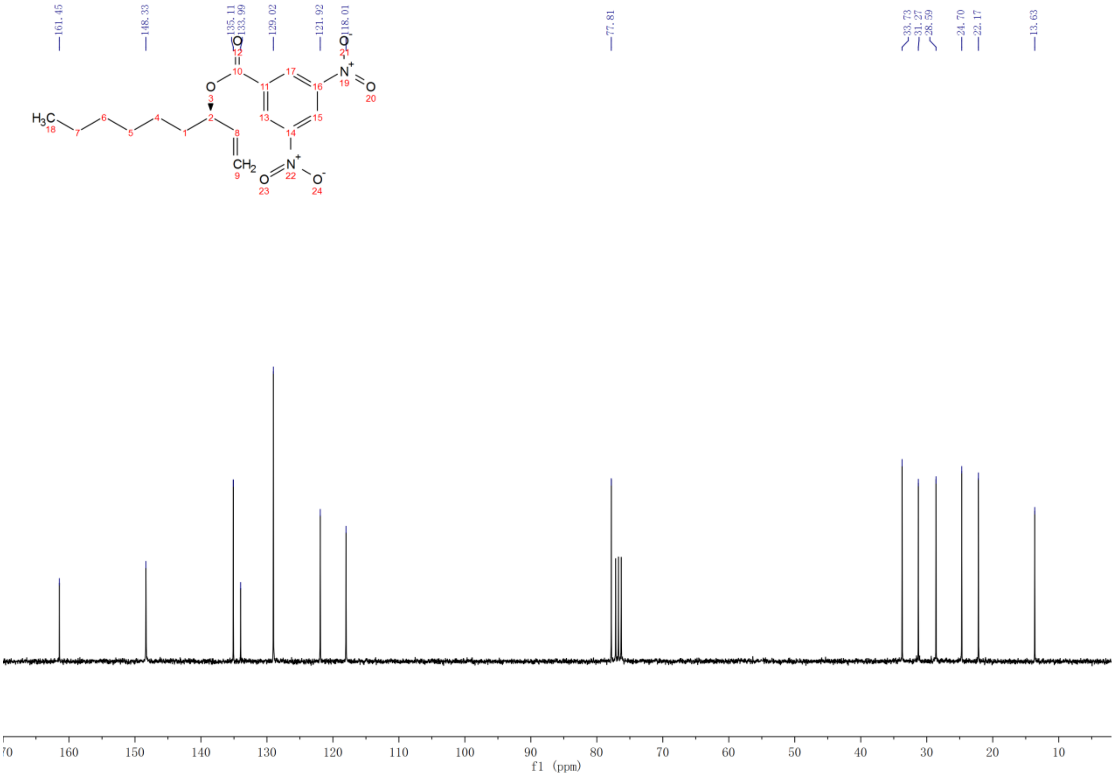

(Compound 21c). (*R*)-dec-1-en-3-yl 3,5-dinitrobenzoate: ^{1}H NMR

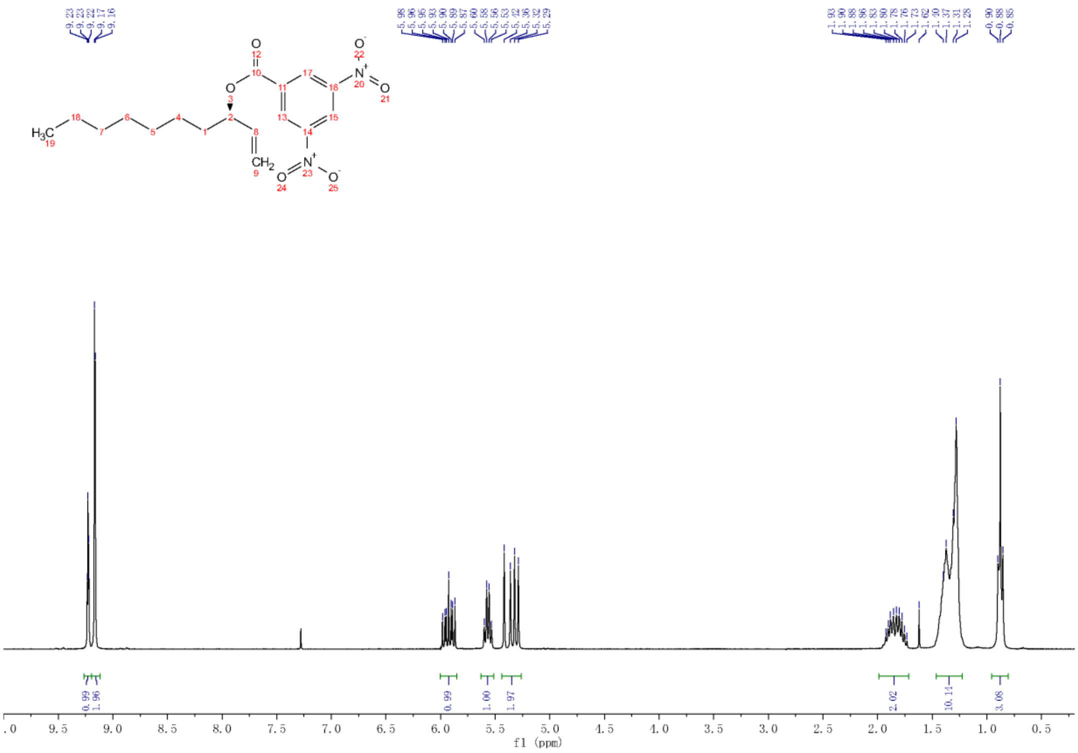

(Compound 21c). (*R*)-dec-1-en-3-yl 3,5-dinitrobenzoate: ^{13}C NMR

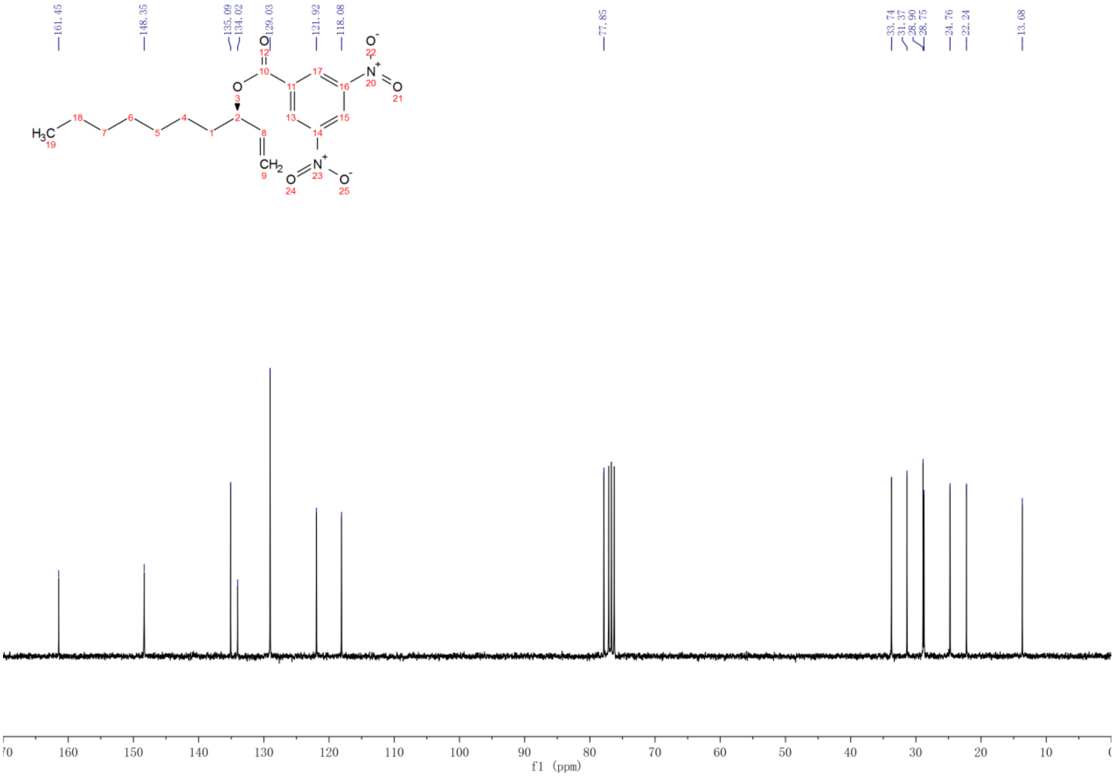

3. HPLC chromatography of the products

Method 1:

(Compound 13a). (*R*)-methyl-4-hydroxynon-2-ynoate:

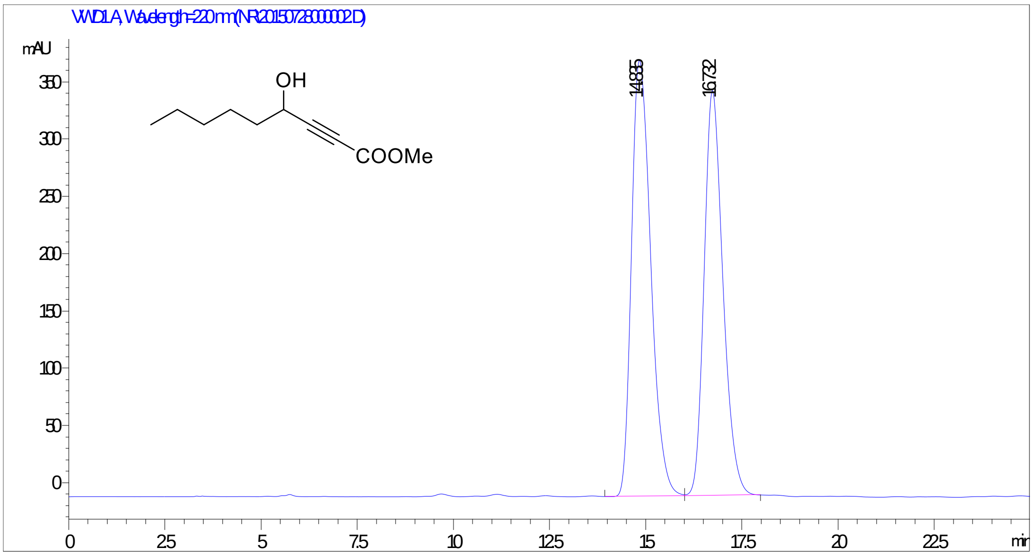

Peak #	RetTime [min]	Type	Width [min]	Area mAU *s	Height [mAU]	Area %
1	14.835	VV	0.5544	1.32896e4	380.58087	52.4920
2	16.732	VB	0.5220	1.20278e4	353.64169	47.5080
Totals :				2.53174e4	734.22256	

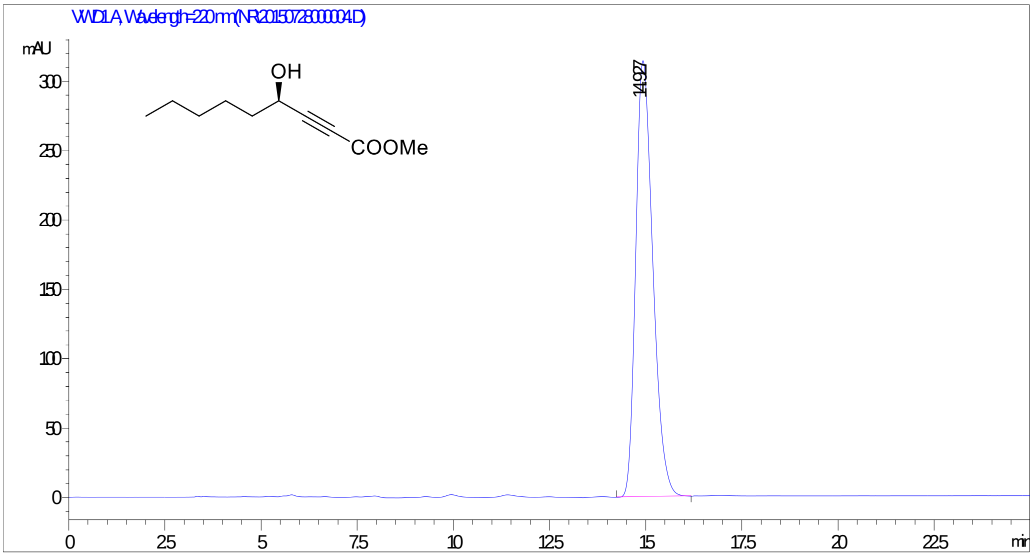

Peak #	RetTime [min]	Type	Width [min]	Area mAU *s	Height [mAU]	Area %
1	14.927	VV	0.4732	9737.32324	314.64587	100.0000
Totals :				9737.32324	314.64587	

(Compound 13b). (*R*)-methyl-4-hydroxydec-2-ynoate:

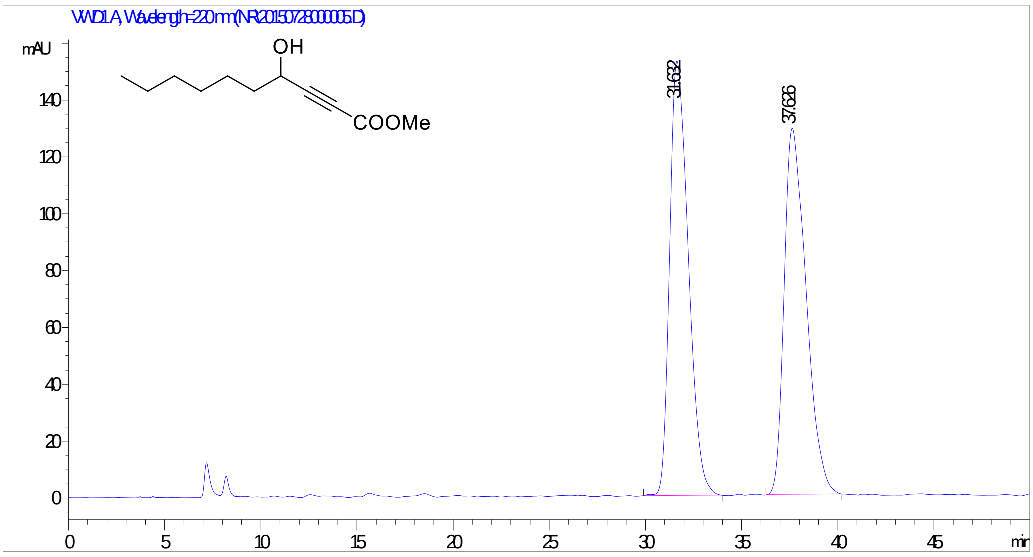

Peak #	RetTime [min]	Type	Width [min]	Area mAU *s	Height [mAU]	Area %
1	31.632	BB	1.0613	1.03554e4	152.83684	50.2757
2	37.626	BB	1.1315	1.02418e4	128.81169	49.7243
Totals :				2.05972e4	281.64853	

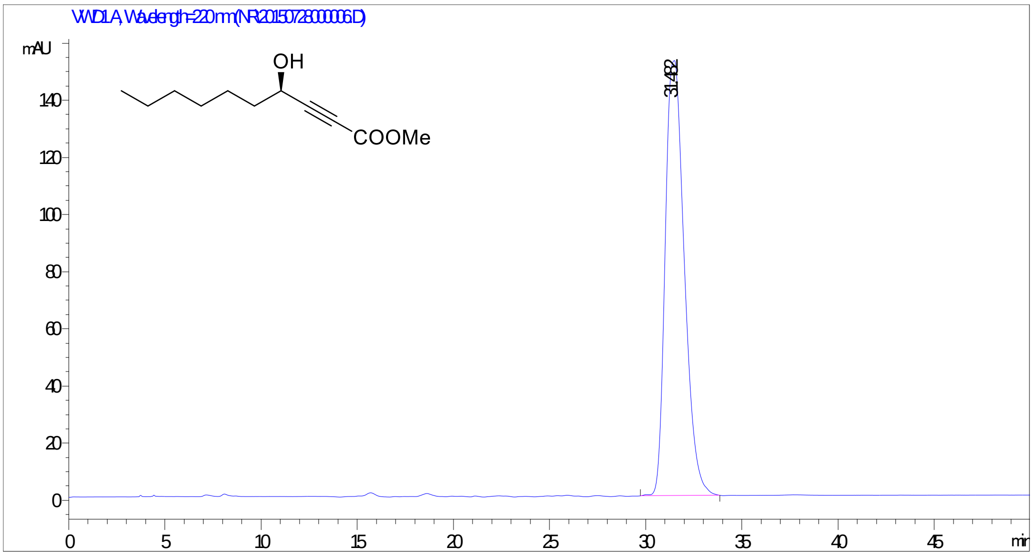

Peak #	RetTime [min]	Type	Width [min]	Area mAU *s	Height [mAU]	Area %
1	31.482	BB	1.0601	1.05157e4	152.34047	100.0000
Totals :				1.05157e4	152.34047	

(Compound 13c). (*R*)-methyl-4-hydroxyundec-2-ynoate:

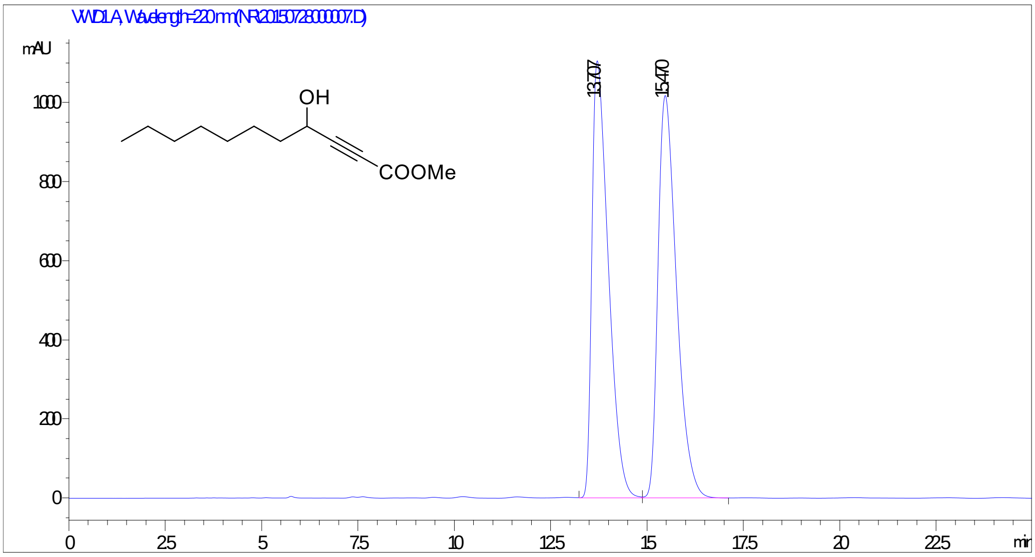

Peak #	RetTime [min]	Type	Width [min]	Area mAU *s	Height [mAU]	Area %
1	13.707	VV	0.4592	3.27592e4	1105.79456	48.9939
2	15.470	VB	0.5189	3.41046e4	1018.19250	51.0061

Totals : 6.68638e4 2123.98706

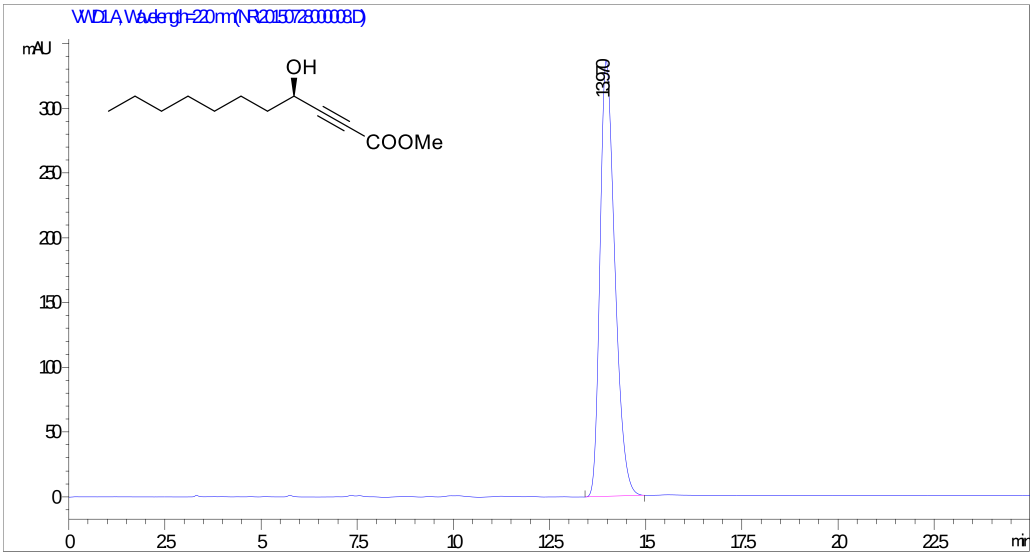

Peak #	RetTime [min]	Type	Width [min]	Area mAU *s	Height [mAU]	Area %
1	13.970	BB	0.4139	9042.82324	336.57712	100.0000

Totals : 9042.82324 336.57712

(Compound 13d). (*R*)-methyl-4-hydroxypent-2-ynoate:

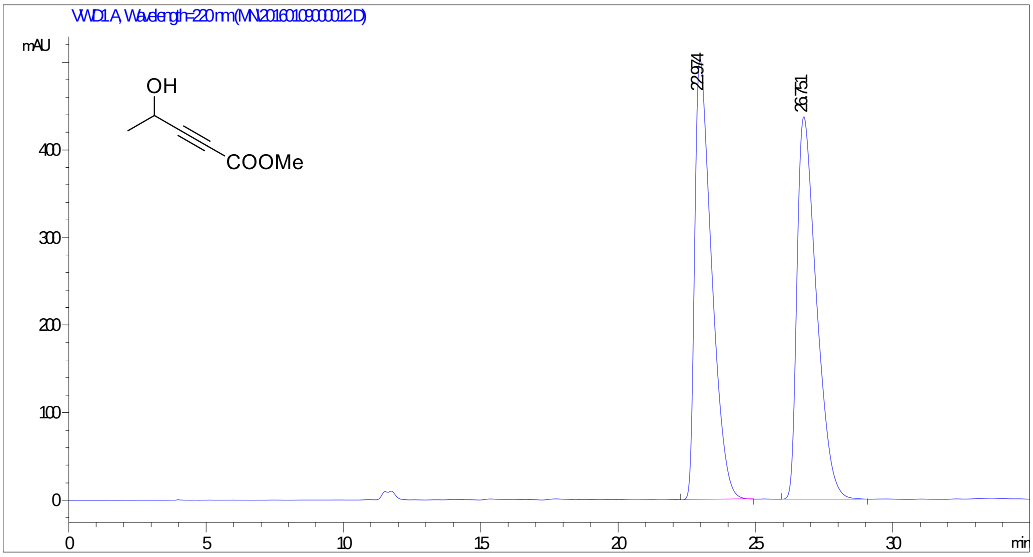

Peak #	RetTime [min]	Type	Width [min]	Area mAU *s	Height [mAU]	Area %
1	22.974	VB	0.6582	2.15579e4	503.53146	50.1114
2	26.751	BB	0.7498	2.14621e4	436.54272	49.8886

Totals : 4.30200e4 940.07419

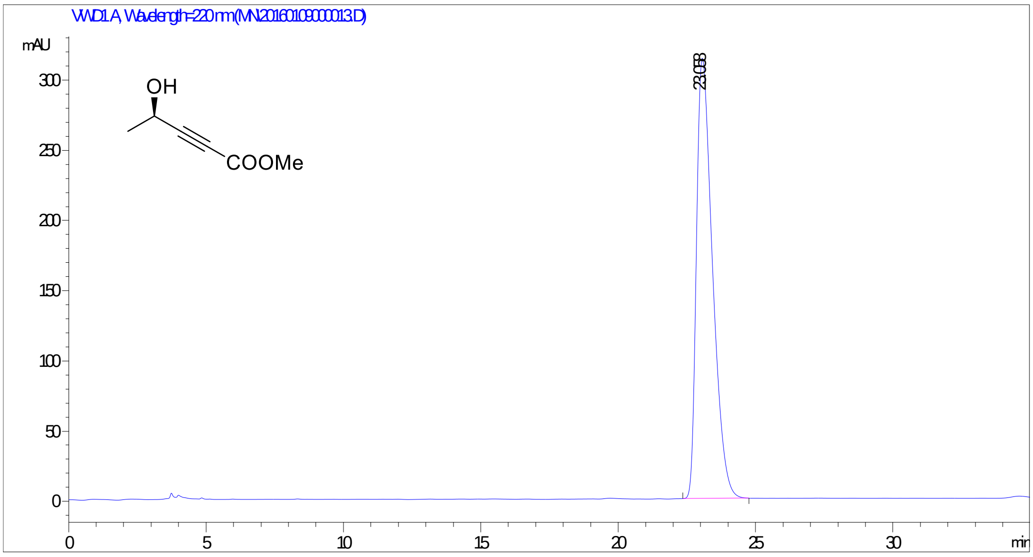

Peak #	RetTime [min]	Type	Width [min]	Area mAU *s	Height [mAU]	Area %
1	23.058	BB	0.6189	1.27253e4	313.64841	100.0000

Totals : 1.27253e4 313.64841

(Compound 13e). (*R*)-methyl-4-hydroxyhex-2-ynoate:

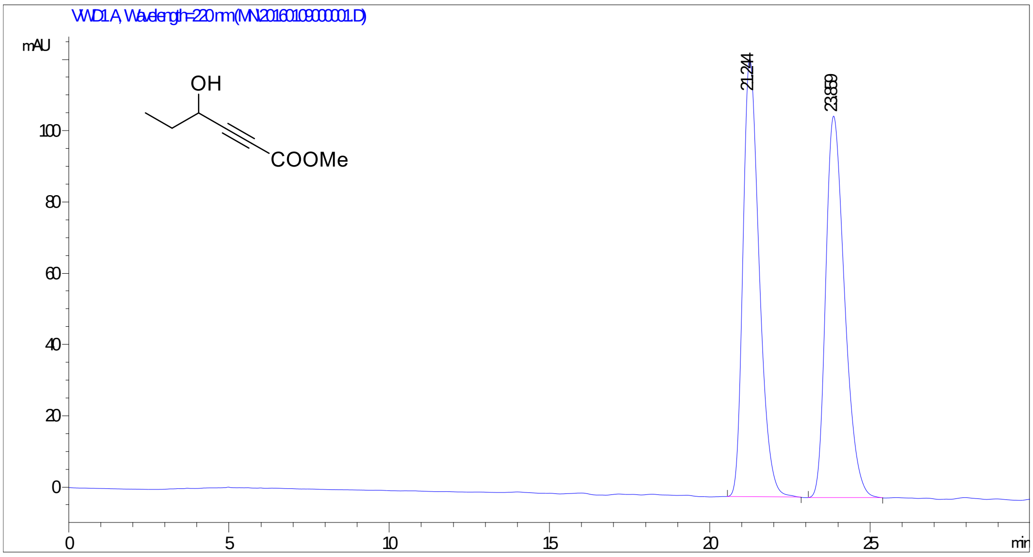

Peak #	RetTime [min]	Type	Width [min]	Area mAU *s	Height [mAU]	Area %
1	21.244	BB	0.5459	4343.53711	123.02674	49.7221
2	23.859	BB	0.6342	4392.09082	107.10086	50.2779

Totals : 8735.62793 230.12760

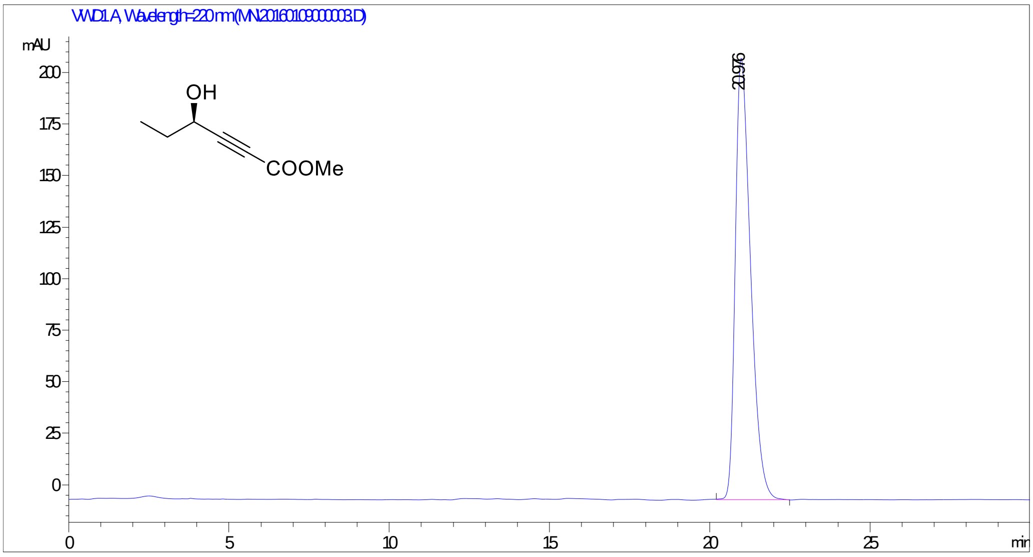

Peak #	RetTime [min]	Type	Width [min]	Area mAU *s	Height [mAU]	Area %
1	20.976	BB	0.5121	7107.12207	214.29602	100.0000

Totals : 7107.12207 214.29602

(Compound 13f). (*R*)-methyl-4-hydroxyhept-2-ynoate:

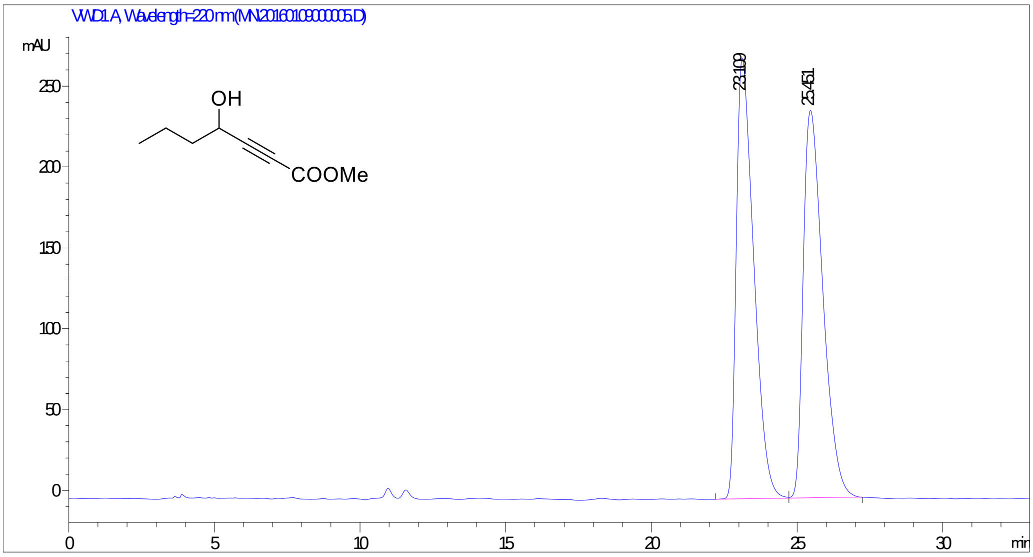

Peak #	RetTime [min]	Type	Width [min]	Area mAU *s	Height [mAU]	Area %
1	23.109	BV	0.6452	1.13632e4	272.47534	50.1153
2	25.451	VB	0.7338	1.13109e4	239.77907	49.8847

Totals : 2.26740e4 512.25441

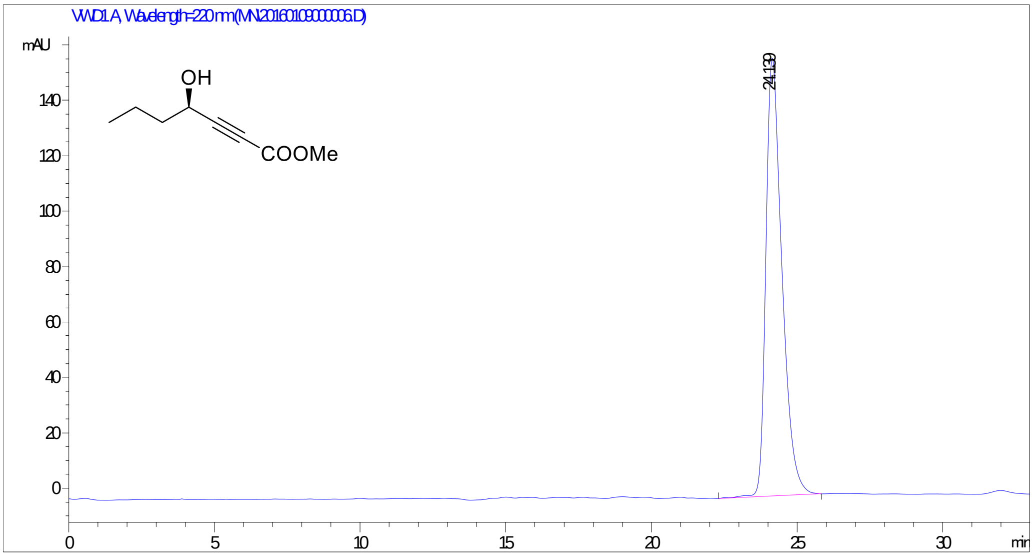

Peak #	RetTime [min]	Type	Width [min]	Area mAU *s	Height [mAU]	Area %
1	24.139	BB	0.5759	6065.17236	158.02617	100.0000

Totals : 6065.17236 158.02617

(Compound 13g). (*R*)-methyl-4-hydroxyoct-2-ynoate:

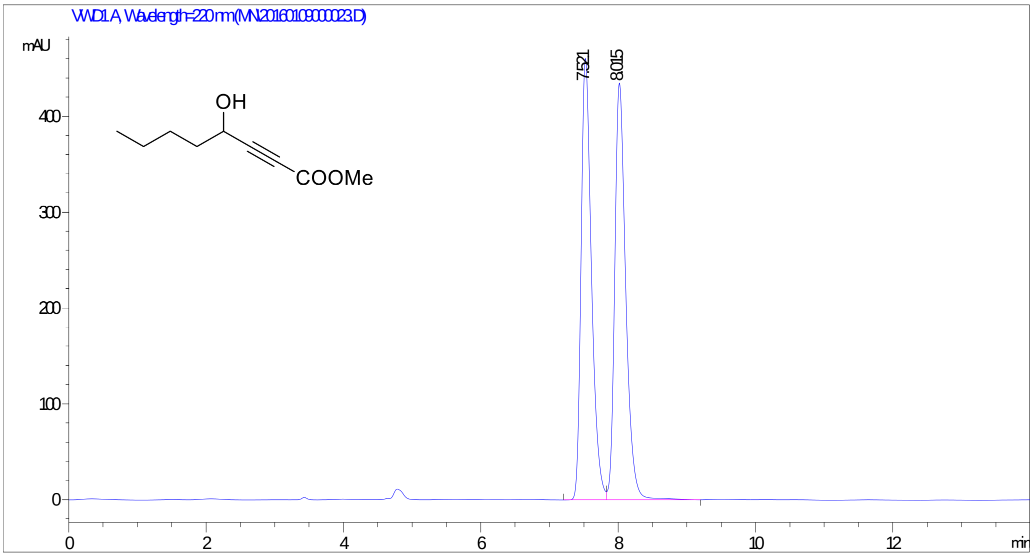

Peak #	RetTime [min]	Type	Width [min]	Area mAU *s	Height [mAU]	Area %
1	7.521	VV	0.1603	4837.69287	460.80420	49.9058
2	8.015	VB	0.1695	4855.96533	435.10864	50.0942

Totals : 9693.65820 895.91284

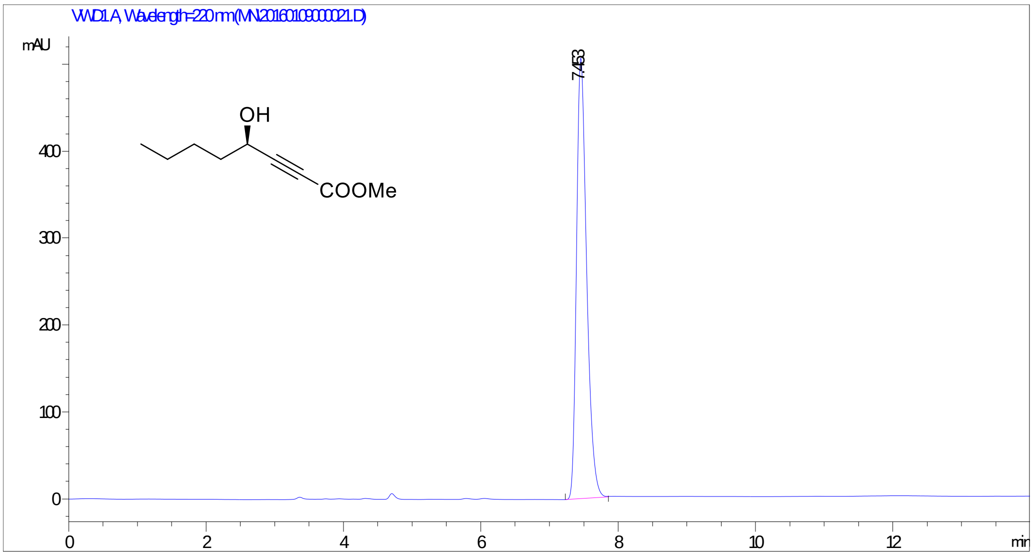

Peak #	RetTime [min]	Type	Width [min]	Area mAU *s	Height [mAU]	Area %
1	7.453	BB	0.1575	5200.53711	507.08368	100.0000

Totals : 5200.53711 507.08368

(Compound 13h). (*R*)-methyl-4-hydroxy-5-methylhex-2-ynoate:

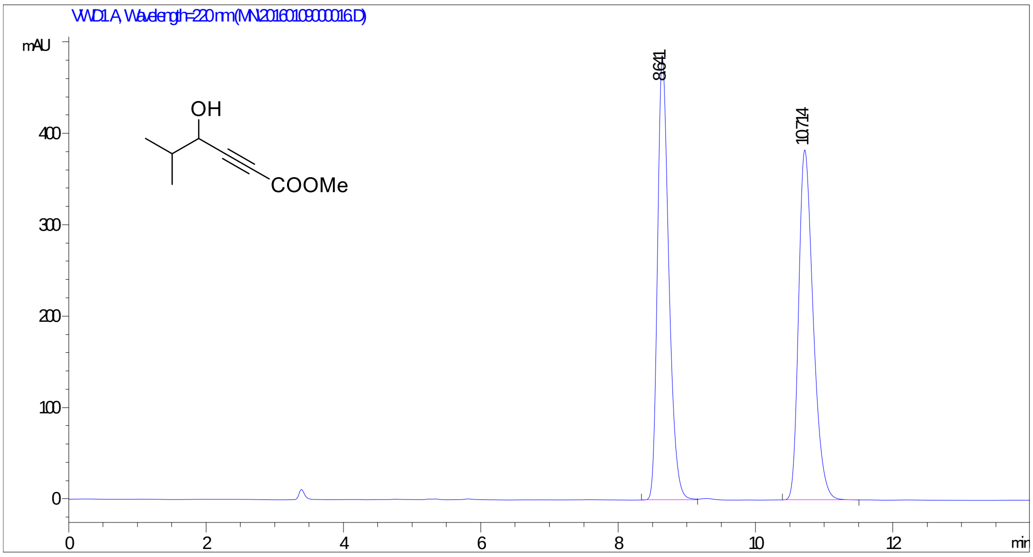

Peak #	RetTime [min]	Type	Width [min]	Area mAU *s	Height [mAU]	Area %
1	8.641	BV	0.1752	5569.80859	483.24014	49.7012
2	10.714	BB	0.2256	5636.77148	383.03427	50.2988

Totals : 1.12066e4 866.27441

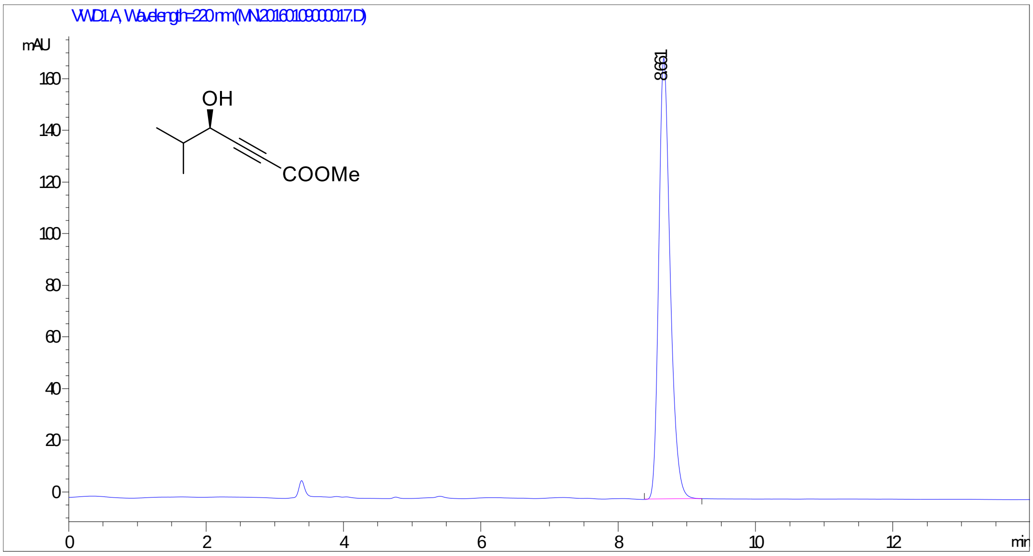

Peak #	RetTime [min]	Type	Width [min]	Area mAU *s	Height [mAU]	Area %
1	8.661	VB	0.1756	1974.89514	170.82173	100.0000

Totals : 1974.89514 170.82173

(Compound 13i). (*R*)-methyl-4-hydroxyoct-7-en-2-ynoate:

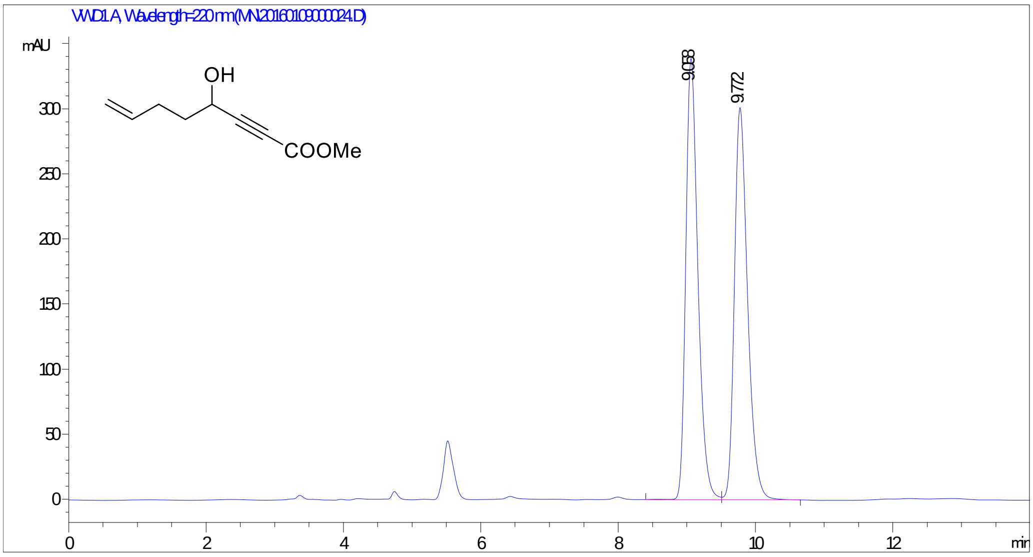

Peak #	RetTime [min]	Type	Width [min]	Area mAU *s	Height [mAU]	Area %
1	9.058	BV	0.1872	4174.93066	339.26898	50.8357
2	9.772	VB	0.2043	4037.67041	301.49152	49.1643

Totals : 8212.60107 640.76050

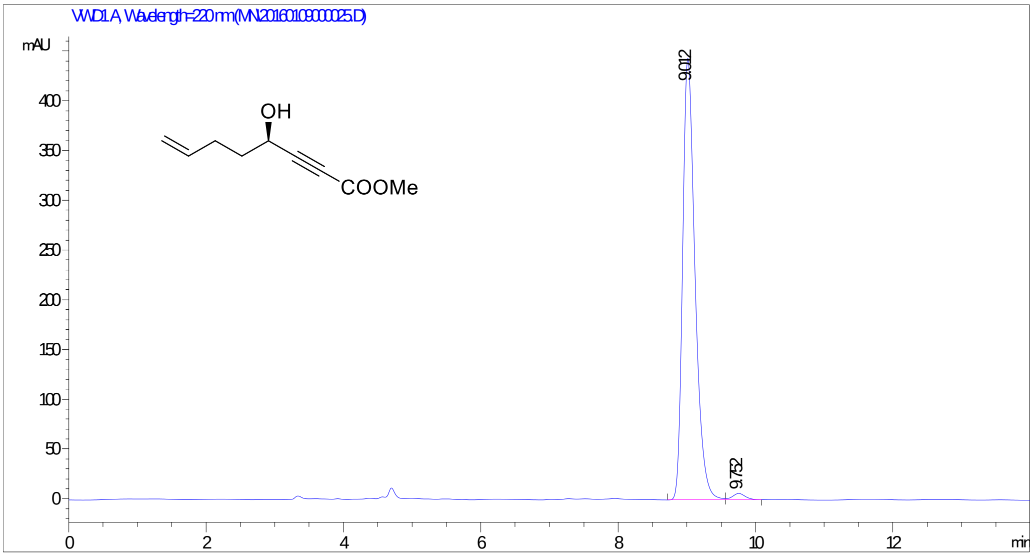

Peak #	RetTime [min]	Type	Width [min]	Area mAU *s	Height [mAU]	Area %
1	9.012	VV	0.1916	5572.63525	444.02283	98.3702
2	9.752	VV	0.2088	92.32597	6.64117	1.6298

Totals : 5664.96122 450.66400

(Compound 21a). (*R*)-oct-1-en-3-yl 3,5-dinitrobenzoate:

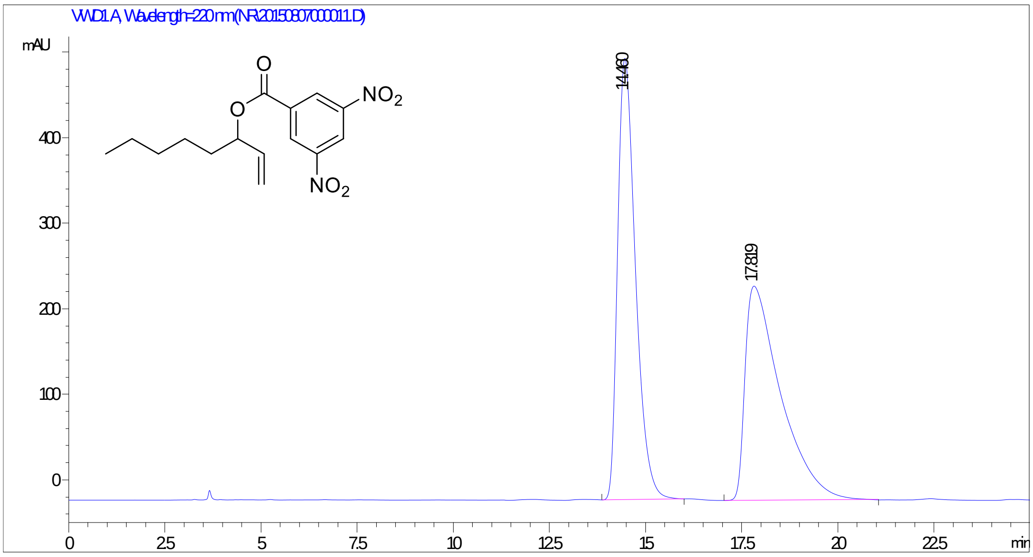

Peak #	RetTime [min]	Type	Width [min]	Area mAU *s	Height [mAU]	Area %
1	14.460	VB	0.4985	1.66321e4	515.80469	50.6955
2	17.819	BB	0.9457	1.61757e4	250.44746	49.3045

Totals : 3.28078e4 766.25215

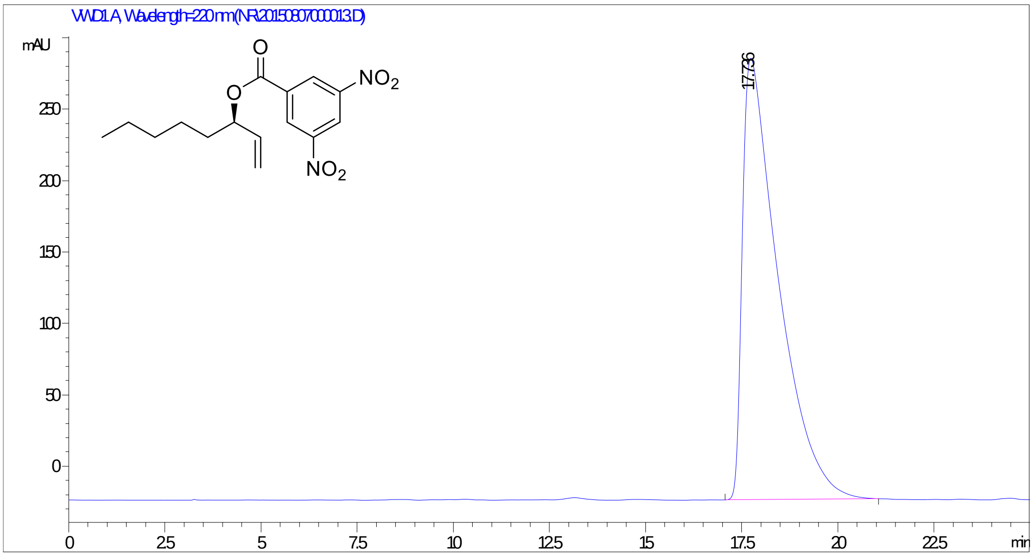

Peak #	RetTime [min]	Type	Width [min]	Area mAU *s	Height [mAU]	Area %
1	17.736	BB	0.9368	2.00684e4	309.00565	100.0000

Totals : 2.00684e4 309.00565

(Compound 21b). (*R*)-non-1-en-3-yl 3,5-dinitrobenzoate:

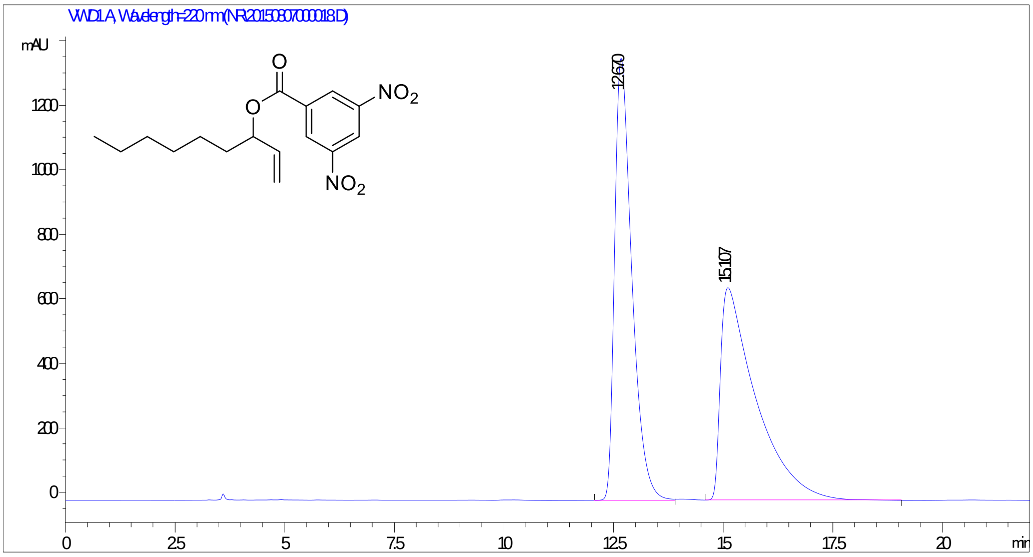

Peak #	RetTime [min]	Type	Width [min]	Area mAU *s	Height [mAU]	Area %
1	12.670	BV	0.4165	3.74635e4	1370.17786	50.1213
2	15.107	VB	0.7908	3.72822e4	659.28857	49.8787

Totals : 7.47457e4 2029.46643

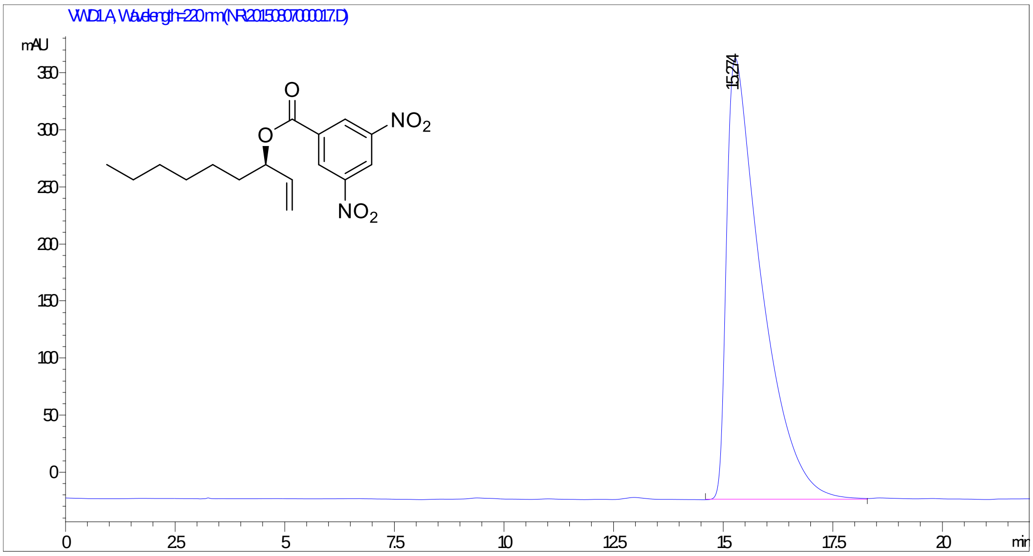

Peak #	RetTime [min]	Type	Width [min]	Area mAU *s	Height [mAU]	Area %
1	15.274	BB	0.7970	2.14520e4	386.83075	100.0000

Totals : 2.14520e4 386.83075

(Compound 21c). (*R*)-dec-1-en-3-yl 3,5-dinitrobenzoate:

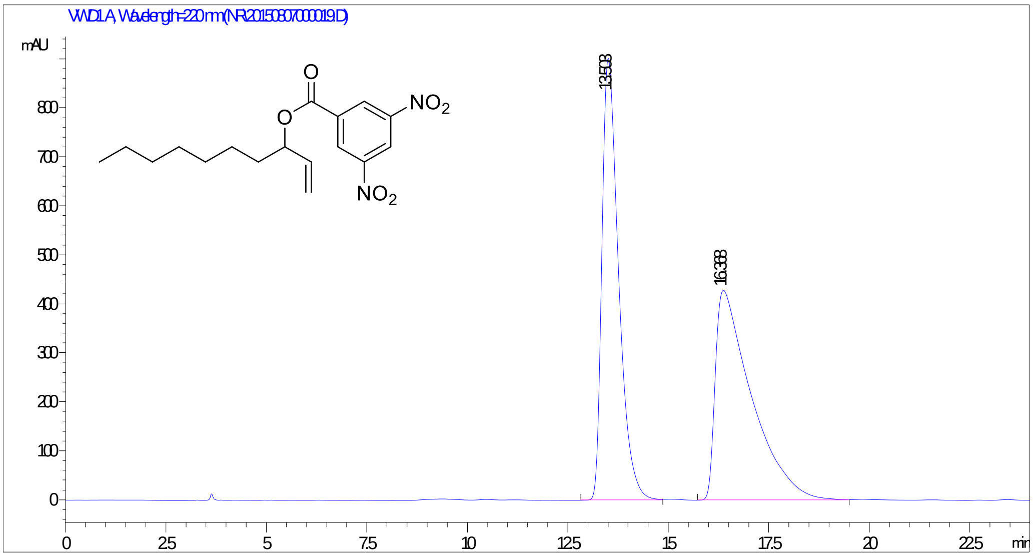

Peak #	RetTime [min]	Type	Width [min]	Area mAU *s	Height [mAU]	Area %
1	13.503	BB	0.4416	2.60297e4	901.88123	49.1598
2	16.368	VB	0.8872	2.69195e4	428.55377	50.8402

Totals : 5.29492e4 1330.43500

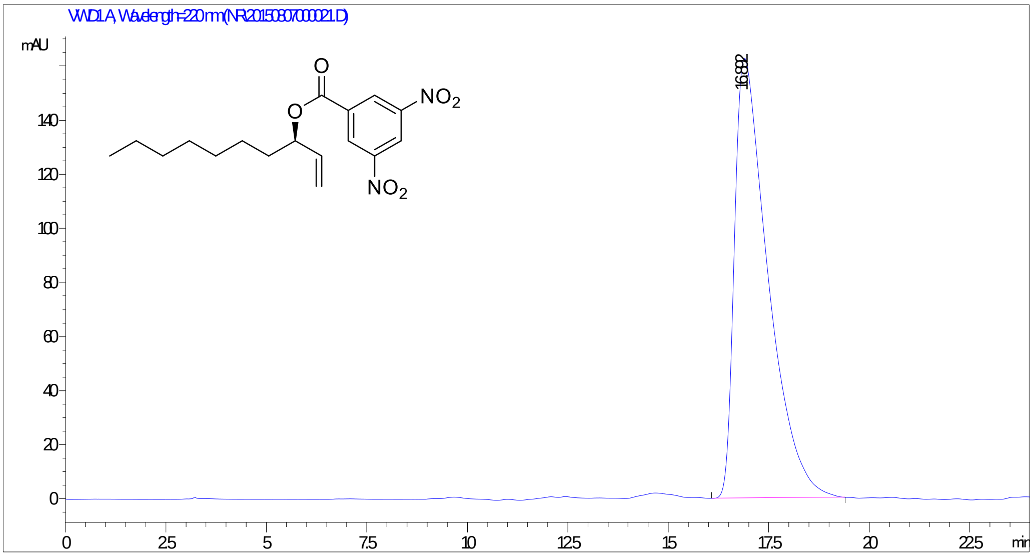

Peak #	RetTime [min]	Type	Width [min]	Area mAU *s	Height [mAU]	Area %
1	16.892	VB	0.8564	9441.58789	162.63931	100.0000

Totals : 9441.58789 162.63931

Method 2: (scheme 4)

(Compound 13a'). (*R*)-1-(trimethylsilyl)oct-1-yn-3-ol:

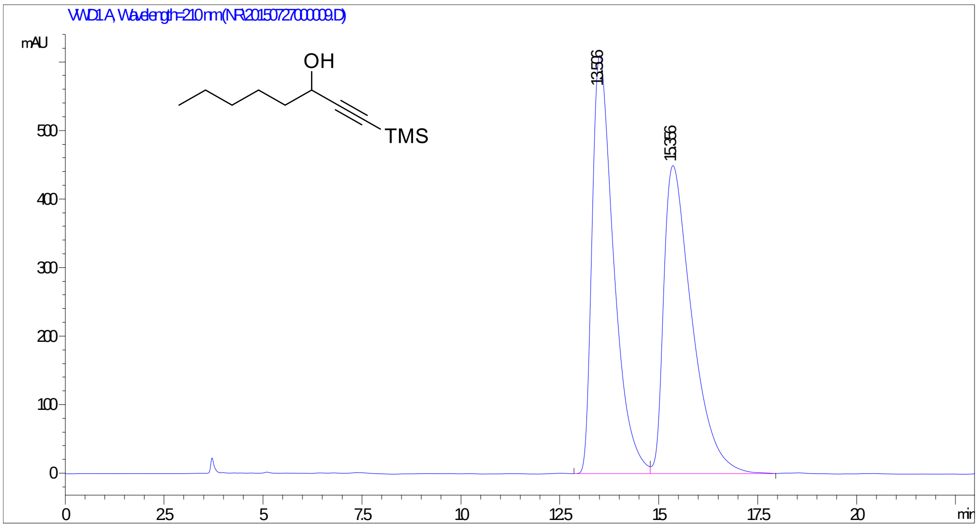

Peak #	RetTime [min]	Type	Width [min]	Area mAU *s	Height [mAU]	Area %
1	13.506	VV	0.5828	2.33410e4	610.66656	50.9495
2	15.356	VV	0.7428	2.24710e4	449.96042	49.0505

Totals : 4.58119e4 1060.62698

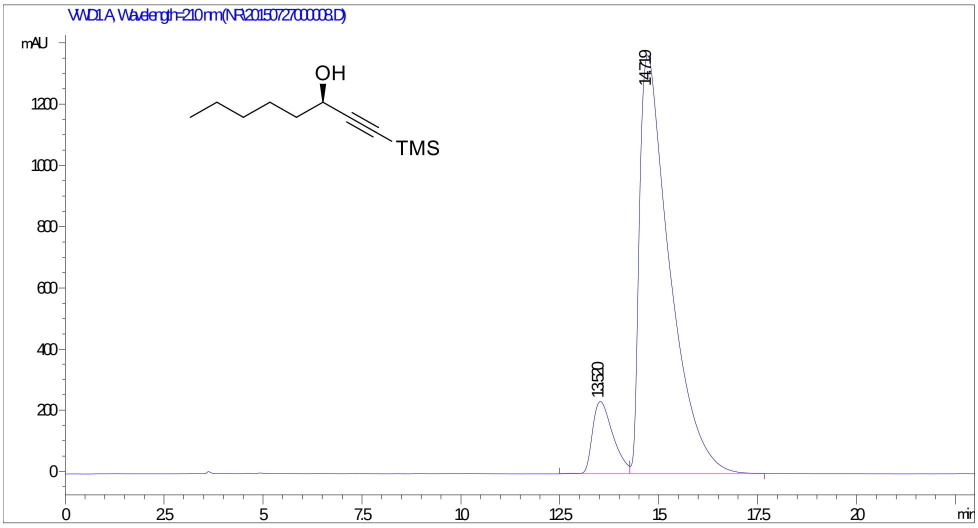

Peak #	RetTime [min]	Type	Width [min]	Area mAU *s	Height [mAU]	Area %
1	13.521	MF	0.5809	8095.20117	232.25613	9.9193
2	147.19	FM	0.8960	7.35153e4	1367.53564	90.0807

Totals : 8.16105e4 1599.79178

(Compound 13b’). (*R*)-1-(trimethylsilyl)non-1-yn-3-ol:

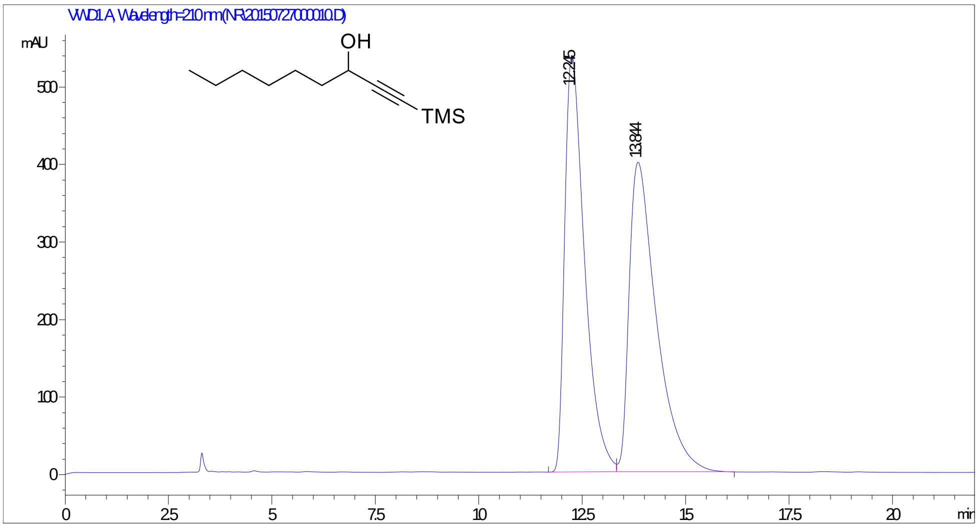

Peak #	RetTime [min]	Type	Width [min]	Area mAU *s	Height [mAU]	Area %
1	12.245	BV	0.5100	1.79936e4	537.42029	49.4476
2	13.844	VB	0.6798	1.83957e4	399.60403	50.5524

Totals : 3.63893e4 937.02432

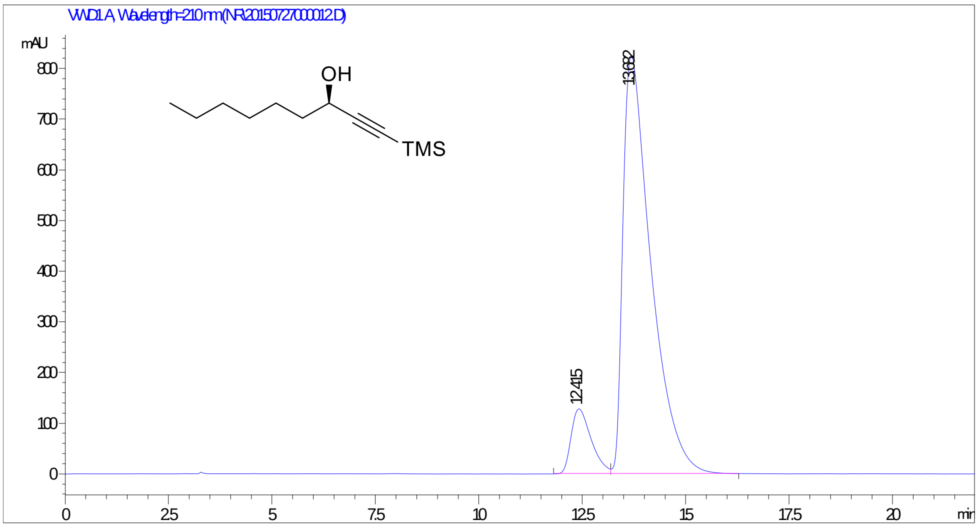

Peak #	RetTime [min]	Type	Width [min]	Area mAU *s	Height [mAU]	Area %
1	12.415	MM	0.5093	3700.27271	121.09660	8.8883
2	13.682	MM	0.7745	3.79305e4	816.28894	91.1117

Totals : 4.16308e4 937.38554

(Compound 13c’). (*R*)-1-(trimethylsilyl)dec-1-yn-3-ol:

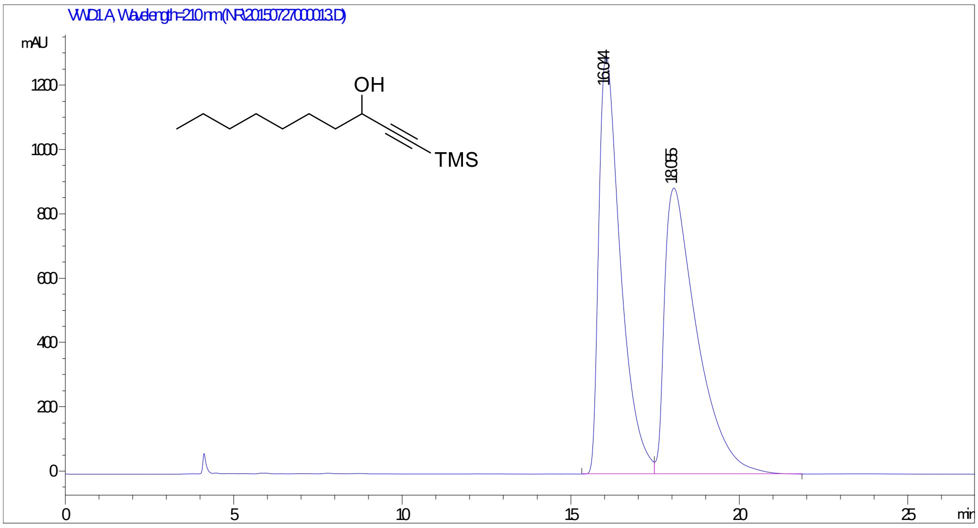

Peak #	RetTime [min]	Type	Width [min]	Area mAU *s	Height [mAU]	Area %
1	16.044	BV	0.6759	5.85091e4	1301.45789	49.5367
2	18.055	VB	0.9801	5.96035e4	889.09644	50.4633

Totals : 1.18113e5 2190.55432

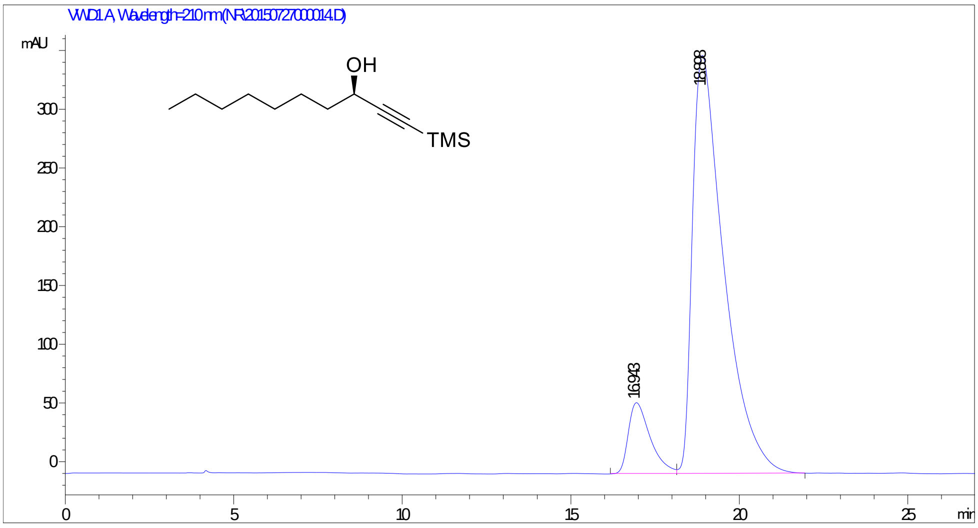

Peak #	RetTime [min]	Type	Width [min]	Area mAU *s	Height [mAU]	Area %
1	16.943	BV	0.6903	2750.04028	60.51877	10.6305
2	18.898	VB	0.9475	2.31192e4	355.75580	89.3695

Totals : 2.58693e4 416.27457

(Compound 21a). (*R*)-oct-1-en-3-yl 3,5-dinitrobenzoate: Before recrystallization

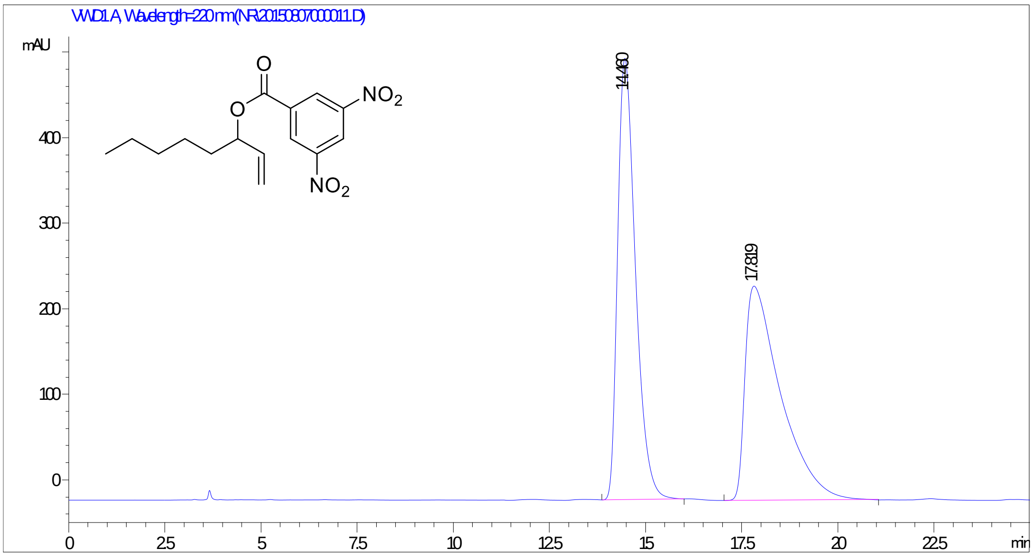

Peak #	RetTime [min]	Type	Width [min]	Area mAU *s	Height [mAU]	Area %
1	14.460	VB	0.4985	1.66321e4	515.80469	50.6955
2	17.819	BB	0.9457	1.61757e4	250.44746	49.3045

Totals : 3.28078e4 766.25215

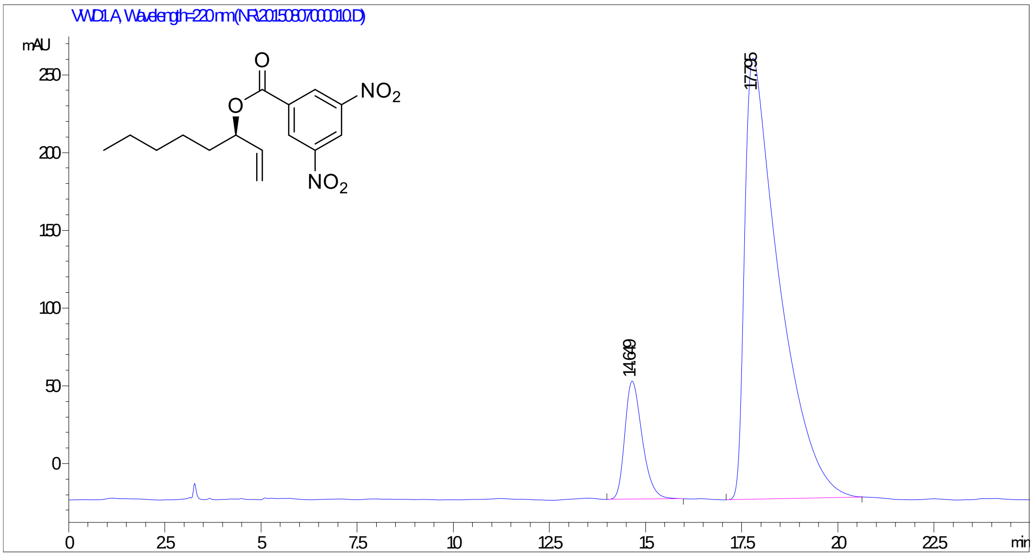

Peak #	RetTime [min]	Type	Width [min]	Area mAU *s	Height [mAU]	Area %
1	14.649	VB	0.4720	1979.46326	69.90018	10.0256
2	17.795	BB	0.9012	1.77646e4	283.57391	89.9744

Totals : 1.97441e4 353.47410

(Compound 21a). (*R*)-oct-1-en-3-yl 3,5-dinitrobenzoate: After recrystallization

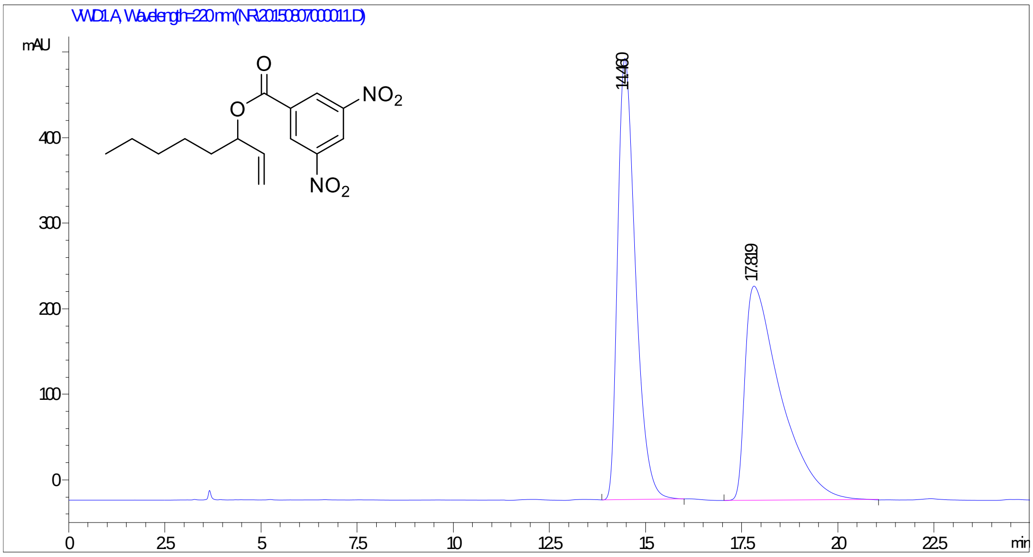

Peak #	RetTime [min]	Type	Width [min]	Area mAU *s	Height [mAU]	Area %
1	14.460	VB	0.4985	1.66321e4	515.80469	50.6955
2	17.819	BB	0.9457	1.61757e4	250.44746	49.3045

Totals : 3.28078e4 766.25215

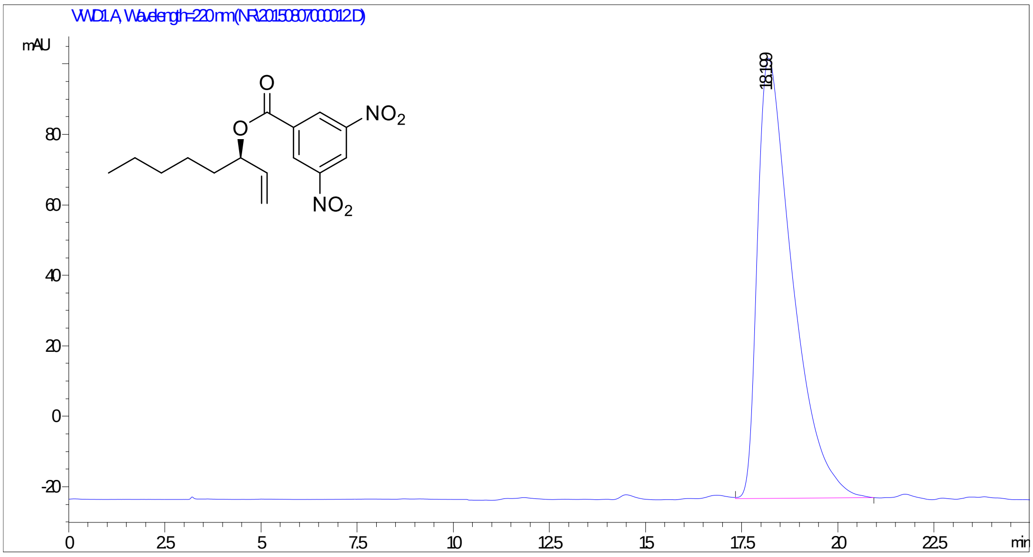

Peak #	RetTime [min]	Type	Width [min]	Area mAU *s	Height [mAU]	Area %
1	18.199	VB	0.9431	7937.69531	125.02853	100.0000

Totals : 7937.69531 125.02853

(Compound 21b). (*R*)-non-1-en-3-yl 3,5-dinitrobenzoate: Before recrystallization

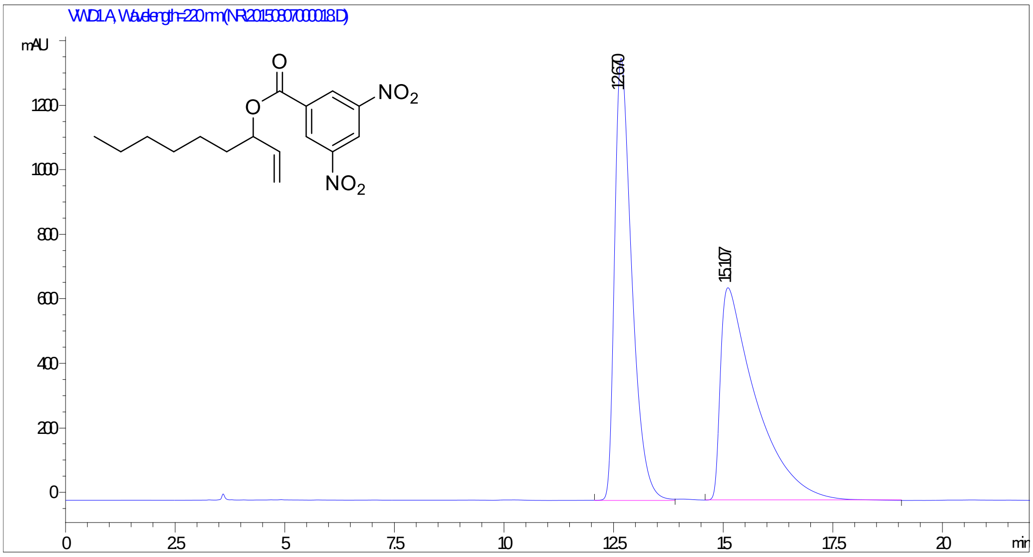

Peak #	RetTime [min]	Type	Width [min]	Area mAU *s	Height [mAU]	Area %
1	12.670	BV	0.4165	3.74635e4	1370.17786	50.1213
2	15.107	VB	0.7908	3.72822e4	659.28857	49.8787

Totals : 7.47457e4 2029.46643

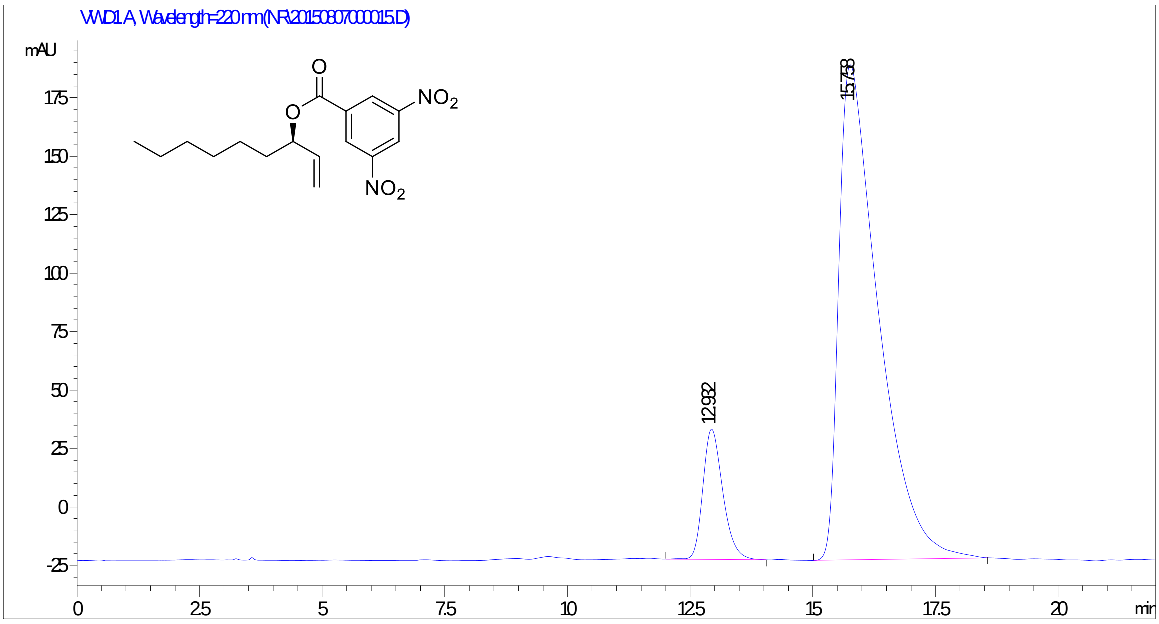

Peak #	RetTime [min]	Type	Width [min]	Area mAU *s	Height [mAU]	Area %
1	12.932	BV	0.4012	1179.03784	48.98094	9.0315
2	15.758	BB	0.8232	1.18757e4	211.74864	90.9685

Totals : 1.30547e4 260.72958

(Compound 21b). (*R*)-non-1-en-3-yl 3,5-dinitrobenzoate: After recrystallization

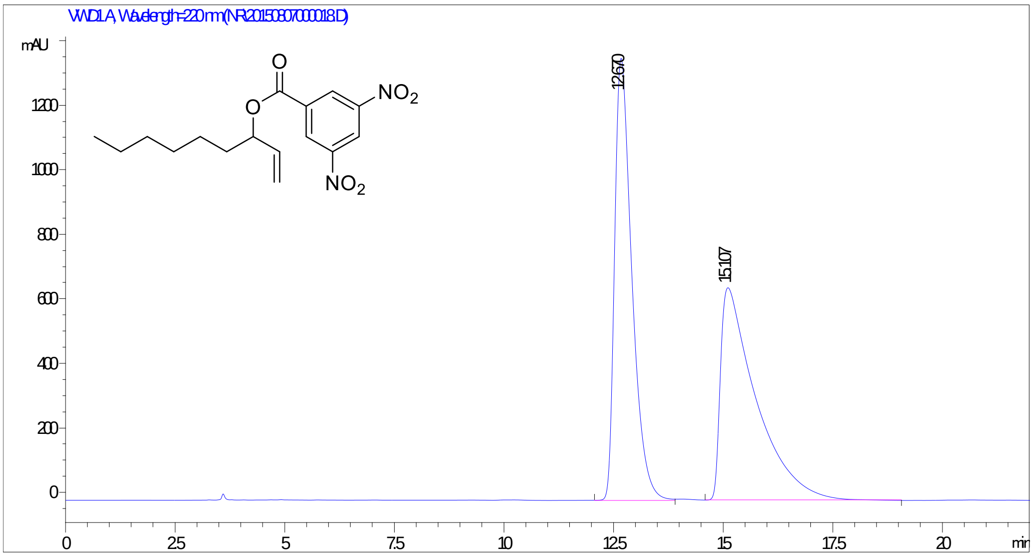

Peak #	RetTime [min]	Type	Width [min]	Area mAU *s	Height [mAU]	Area %
1	12.670	BV	0.4165	3.74635e4	1370.17786	50.1213
2	15.107	VB	0.7908	3.72822e4	659.28857	49.8787
Totals :				7.47457e4	2029.46643	

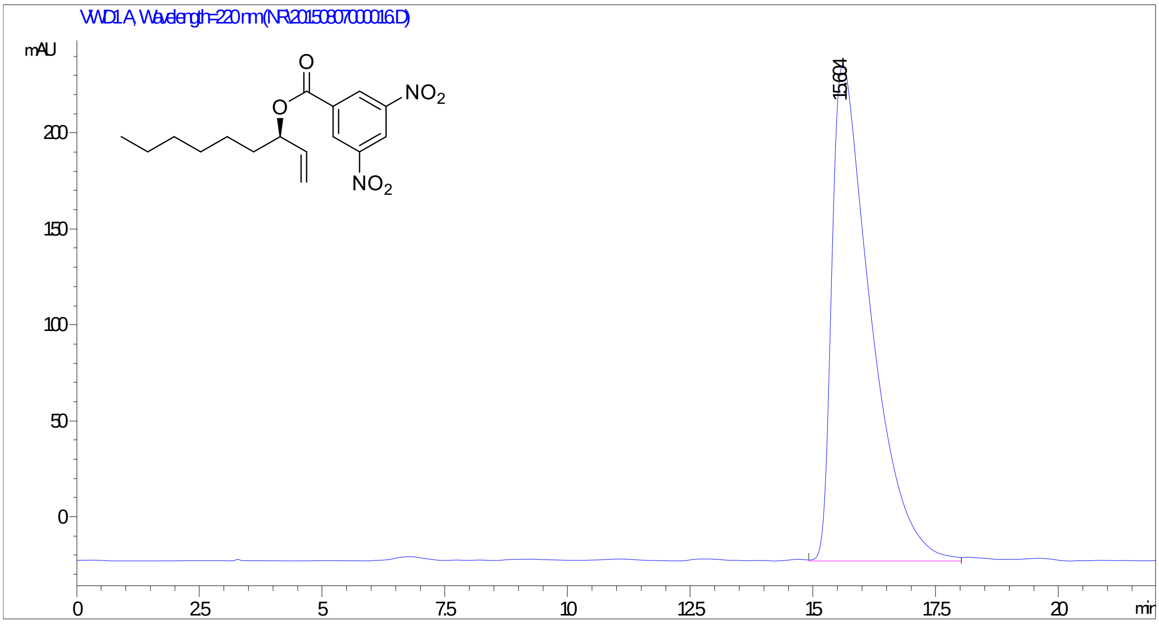

Peak #	RetTime [min]	Type	Width [min]	Area mAU *s	Height [mAU]	Area %
1	15.604	VB	0.7989	1.42491e4	258.48590	100.0000
Totals :				1.42491e4	258.48590	

(Compound 21c). (*R*)-dec-1-en-3-yl 3,5-dinitrobenzoate: Before recrystallization

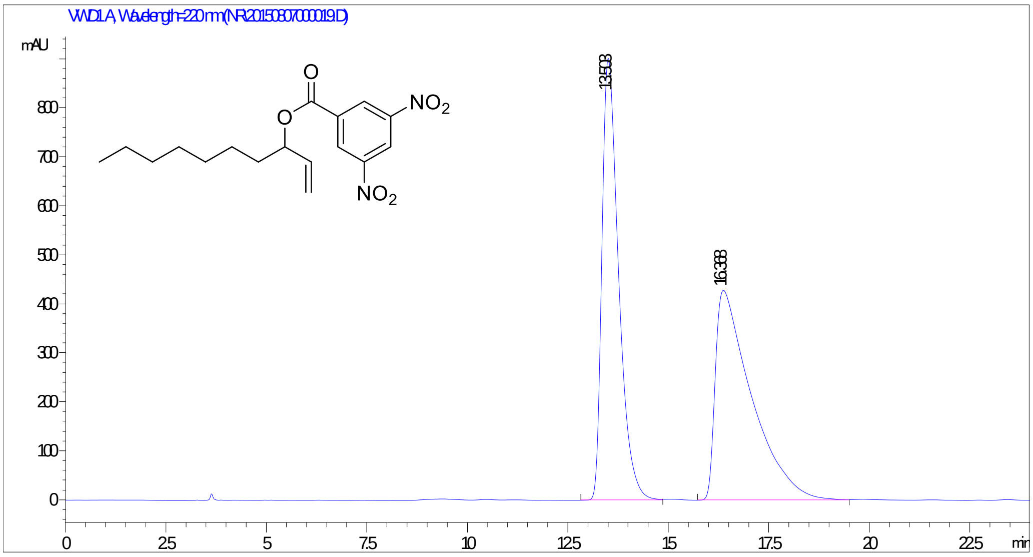

Peak #	RetTime [min]	Type	Width [min]	Area mAU *s	Height [mAU]	Area %
1	13.503	BB	0.4416	2.60297e4	901.88123	49.1598
2	16.368	VB	0.8872	2.69195e4	428.55377	50.8402

Totals : 5.29492e4 1330.43500

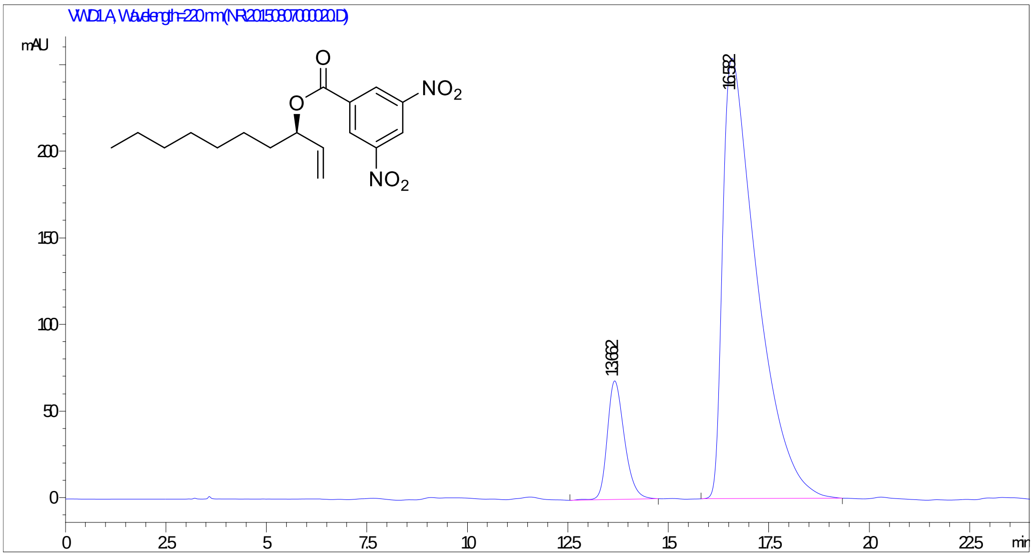

Peak #	RetTime [min]	Type	Width [min]	Area mAU *s	Height [mAU]	Area %
1	13.662	VB	0.4573	1798.28528	65.53643	10.6498
2	16.582	BB	0.8597	1.50874e4	254.25864	89.3502

Totals : 1.68857e4 319.79507

(Compound 21c). (*R*)-dec-1-en-3-yl 3,5-dinitrobenzoate: After recrystallization

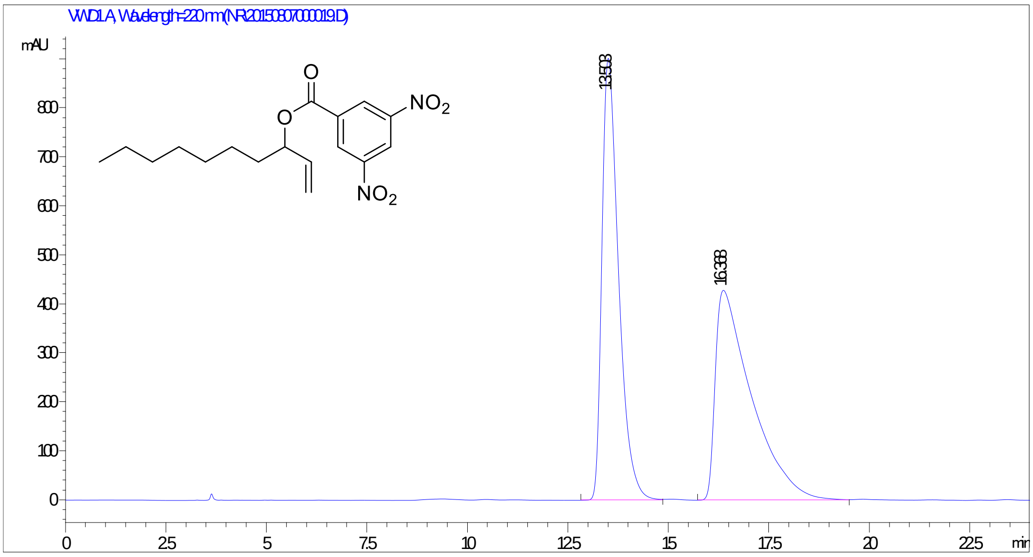

Peak #	RetTime [min]	Type	Width [min]	Area mAU *s	Height [mAU]	Area %
1	13.503	BB	0.4416	2.60297e4	901.88123	49.1598
2	16.368	VB	0.8872	2.69195e4	428.55377	50.8402

Totals : 5.29492e4 1330.43500

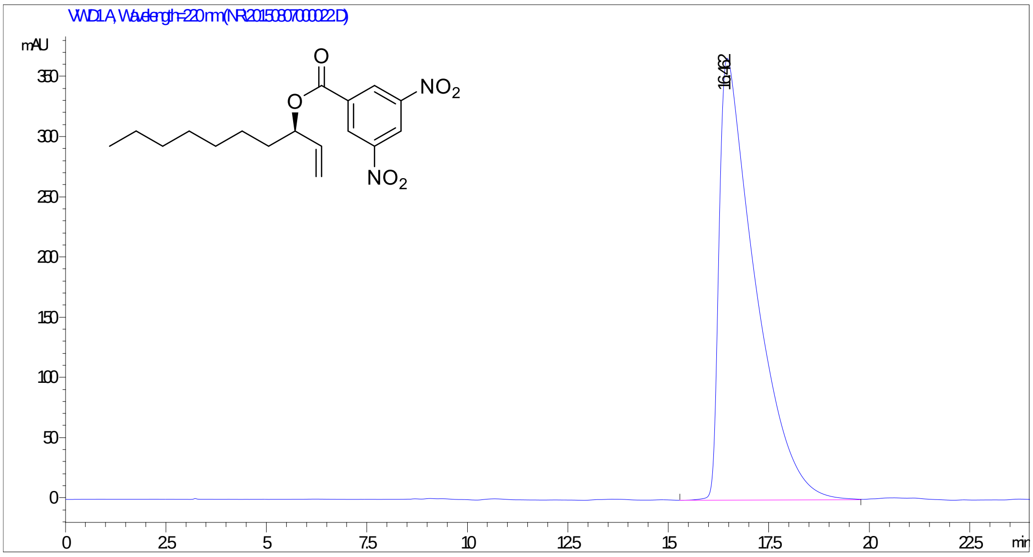

Peak #	RetTime [min]	Type	Width [min]	Area mAU *s	Height [mAU]	Area %
1	16.462	VB	0.8862	2.28911e4	367.10037	100.0000

Totals : 2.28911e4 367.10037
